# Supplementary figures and images for: Seroprevalence of Dengue, Chikungunya and Zika at the epicenter of the congenital microcephaly epidemic in Northeast Brazil: A population-based survey
Source: PLoS Negl Trop Dis. 2023 Jul 3;17(7):e0011270. doi: 10.1371/journal.pntd.0011270 (PMC10348596; doi:10.1371/journal.pntd.0011270)

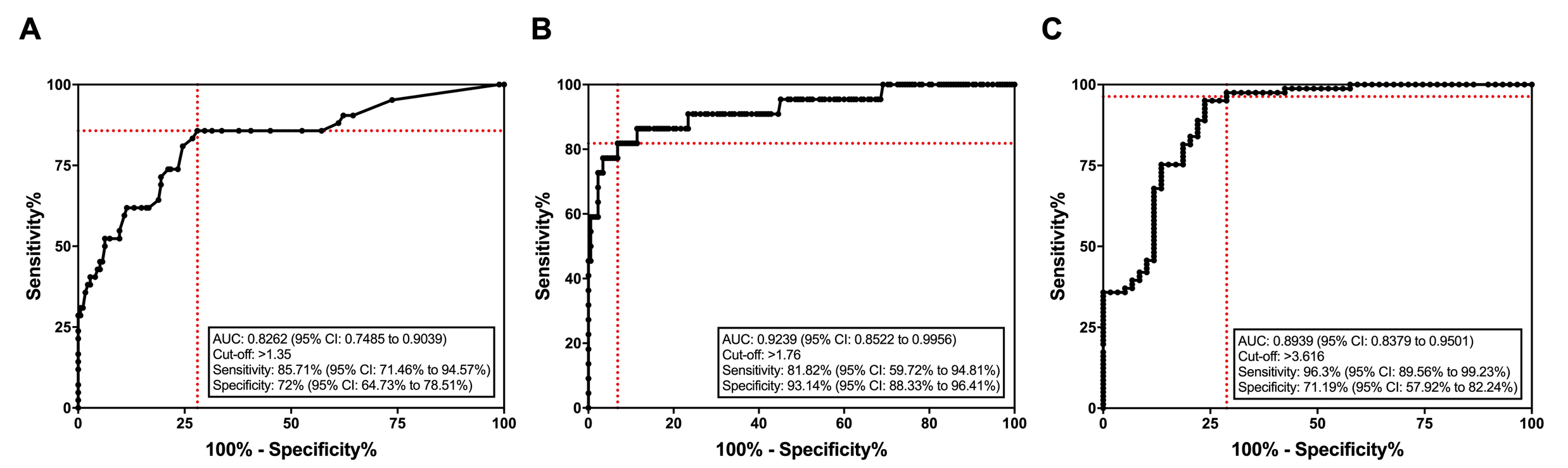

Supplement: S1 Fig — The paired results for sensitivity and specificity were plotted as points in a ROC space and the trade-off between these measures for different discrimination cut-offs are graphically represented. The red dotted line represents the cut-off value. (TIF) [file pntd.0011270.s001.tif]
