# Supplementary material for: Seroprevalence of Dengue, Chikungunya and Zika at the epicenter of the congenital microcephaly epidemic in Northeast Brazil: A population-based survey
Source: PLoS Negl Trop Dis. 2023 Jul 3;17(7):e0011270. doi: 10.1371/journal.pntd.0011270 (PMC10348596; doi:10.1371/journal.pntd.0011270)
Supplement: S1 Table — (DOCX) [file pntd.0011270.s002.docx]

**S1 Table. List of exposure variables.**

| Household level | Categorization |
| --- | --- |
| type of dwelling | house or apartment |
| number of residents per bedroom |  |
| Sewerage | Public network, other waste destination |
| Water supply | Public network, well, other waste sources |
| Frequency of water supply | Regular, irregular |
| Garbage collection | Household garbage collection, other destination |
| **Characteristics of the head of the family** |  |
| **Sex** | Male, Female |
| Schooling | University, High School, Fundamental |
| Monthly income (in minimum wage) | ≤2, 2-4, >4-20 |
| Self-report skin color | Mixed, Black, White, others (Asiatic, indigen, not informed, ignored) |
| Individual level |  |
| Age group | 5-14, 15-24, 25-34, 35-44, 45-54, 55-65 |
| Sex | Male, female |
| Self-report race/skin color | Mixed, Black, White, others (Asiatic, indigen, not informed, ignored) |
| Schooling (≥13 years) | University, High School, Fundamental |
| Monthly income (in minimum wage) | ≤2, 2-4, >4-20 |
| Commuting to study and/or work outside the neighborhood of residence | Yes, No, Neither study nor work, not informed |
| Repellent use | No, daily use, at least three days a week |
| Previous dengue infection | No, Yes |
| YFV vaccination | No, Yes |
| DENV vaccination | No, Yes |
