# Supplementary material for: Seroprevalence of Dengue, Chikungunya and Zika at the epicenter of the congenital microcephaly epidemic in Northeast Brazil: A population-based survey
Source: PLoS Negl Trop Dis. 2023 Jul 3;17(7):e0011270. doi: 10.1371/journal.pntd.0011270 (PMC10348596; doi:10.1371/journal.pntd.0011270)
Supplement: S2 Table — Recife, Brazil, 2018–2019. (DOCX) [file pntd.0011270.s003.docx]

**S2 Table. Crude analysis of the association between household characteristics and ZIKV infection. Recife, Brazil, 2018-2019.**

| **Characteristics** | **Socioeconomic strata** | | | | | | | | |
| --- | --- | --- | --- | --- | --- | --- | --- | --- | --- |
|  | **High** | | | **Intermediate** | | | **Low** | | |
|  | **Total** | **Positive** | **OR (95%CI)** | **Total** | **Positive** | **OR (95%CI)** | **Total** | **Positive** | **OR (95%CI)** |
|  |  | **n (%)** |  |  | **n (%)** |  |  | **n (%)** |  |
| **Number of residents in the household** |  |  |  |  |  |  |  |  |  |
| Up 2 | 103 | 41 (39.5) | 1.00 | 150 | 78 (52.4) | 1.00 | 115 | 63 (55.2) | 1.00 |
| >2-4 | 213 | 79 (37.0) | 0.90 (0.59-1.38) | 358 | 203 (56.7) | 1.19 (0.78-1.81) | 437 | 248 (56.7) | 1.06 (0.67-1.69) |
| >4 | 100 | 38 (38.3) | 0.95 (0.59-1.53) | 218 | 118 (53.9) | 1.06 (0.70-1.62) | 376 | 205 (54.5) | 0.97 (0.61-1.55) |
| **Type of household** |  |  |  |  |  |  |  |  |  |
| Apartment | 256 | 71 (27.7) | 1.00 | 124 | 43 (34.5) | 1.00 | 30 | 12 (40.0) | 1.00 |
| House | 159 | 87 (54.6) | 3.14 (2.02-4.90) | 598 | 353 (59.0) | 2.73 (1.90-3.93) | 898 | 504 (56.1) | 1.92 (1.10-3.36) |
| **Waste destination** |  |  |  |  |  |  |  |  |  |
| Public network | 290 | 116 (40.0) | 1.00 | 434 | 239 (55.0) | 1.00 | 341 | 198 (57.9) | 1.00 |
| Other destination | 84 | 33 (39.2) | 0.97 (0.60-1.57) | 269 | 148 (55.0) | 0.99 (0.67-1.48) | 575 | 314 (54.6) | 0.87 (0.67-1.14) |
| **Water supply** |  |  |  |  |  |  |  |  |  |
| Public network | 278 | 113 (40.8) | 1.00 | 662 | 371 (56.0) | 1.00 | 831 | 460 (55.3) | 1.00 |
| Well/other sources | 135 | 44 (32.7) | 0.70 (0.43-1.16) | 60 | 25 (41.8) | 0.56 (0.32-0.99) | 90 | 53 (58.7) | 1.15 (0.72-1.83) |
| **Irregularity of water supply** |  |  |  |  |  |  |  |  |  |
| No | 364 | 129 (35.5) | 1.00 | 595 | 319 (53.6) | 1.00 | 532 | 307 (57.7) | 1.00 |
| Yes | 51 | 29 (55.9) | 2.31 (1.12-4.74) | 127 | 77 (60.6) | 1.33 (0.85-2.07) | 394 | 208 (52.9) | 0.82 (0.61-1.12) |
| **Garbage destination** |  |  |  |  |  |  |  |  |  |
| Home collection | 412 | 156 (37.9) | 1.00 | 650 | 356 (54.8) | 1.00 | 850 | 471 (55.4) | 1.00 |
| Other destination | 3 | 2 (33.3) | 1.64 (0.39-6.90) | 71 | 39 (55.0) | 1.01 (0.56-1.80) | 78 | 46 (58.5) | 1.14 (0.69-1.88) |
| **Monthly income (in minimum wages)** |  |  |  |  |  |  |  |  |  |
| No income / Up to 2 | 125 | 64 (51.4) | 1.00 | 418 | 253 (60.6) | 1.00 | 751 | 430 (57.3) | 1.00 |
| >2-4 | 91 | 38 (41.9) | 0.68 (0.38-1.22) | 197 | 101 (51.1) | 0.68 (0.51-0.91) | 148 | 68 (46.0) | 0.64 (0.46-0.89) |
| >4 | 196 | 54 (27.4) | 0.36 (0.22-0.59) | 101 | 40 (39.5) | 0.43 (0.28-0.64) | 17 | 10 (57.1) | 1.00 (0.62-1.60) |
| **Characteristics of the head of the family** |  |  |  |  |  |  |  |  |  |
| **Schooling** |  |  |  |  |  |  |  |  |  |
| University | 253 | 75 (29.8) | 1.00 | 180 | 78 (43.1) | 1.00 | 42 | 23 (54.3) | 1.00 |
| High school | 85 | 44 (52.0) | 2.56 (1.24-5.28) | 256 | 150 (58.5) | 1.87 (1.25-2.79) | 346 | 184 (53.3) | 0.96 (0.36-2.56) |
| Fundamental/ illiterate | 75 | 37 (50.0) | 2.36 (1.44-3.85) | 283 | 168 (59.4) | 1.94 (1.31-2.88) | 525 | 297 (56.6) | 1.10 (0.44-2.74) |
| **Race/ Skin color** |  |  |  |  |  |  |  |  |  |
| White | 194 | 68 (35.3) | 1.00 | 243 | 131 (53.8) | 1.00 | 152 | 85 (55.9) | 1.00 |
| Mixed race | 163 | 68 (42.0) | 1.33 (0.84-2.11) | 361 | 192 (53.3) | 0.98 (0.70-1.38) | 585 | 328 (56.1) | 1.01 (0.68-1.51) |
| Black | 37 | 13 (34.9) | 0.28 (0.57-1.71) | 100 | 66 (66.1) | 1.67 (1.05-2.64) | 171 | 89 (51.7) | 0.85 (0.54-1.33) |
| Other/Ignored | 22 | 8 (36.0) | 1.03 (0.48-2.22) | 22 | 10 (44.0) | 0.67 (0.23-1.98) | 20 | 14 (70.6) | 1.89 (0.98-3.64) |
