# Supplementary material for: Seroprevalence of Dengue, Chikungunya and Zika at the epicenter of the congenital microcephaly epidemic in Northeast Brazil: A population-based survey
Source: PLoS Negl Trop Dis. 2023 Jul 3;17(7):e0011270. doi: 10.1371/journal.pntd.0011270 (PMC10348596; doi:10.1371/journal.pntd.0011270)
Supplement: S3 Table — Recife, Brazil, 2018–2019. (DOCX) [file pntd.0011270.s004.docx]

**S3 Table. Crude analysis of the association between individual characteristics and ZIKV infection. Recife, Brazil, 2018-2019.**

| **Characteristics** | **Socioeconomic strata** | | | | | | | | |
| --- | --- | --- | --- | --- | --- | --- | --- | --- | --- |
|  | **High** | | | **Intermediate** | | | **Low** | | |
|  | **Total** | **Positive** | **OR (IC95%)** | **Total** | **Positive** | **OR (IC95%)** | **Total** | **Positive** | **OR (IC95%)** |
|  |  | **n (%)** |  |  | **n (%)** |  |  | **n (%)** |  |
| **Gender** |  |  |  |  |  |  |  |  |  |
| Female | 222 | 84 (37.9) | 1.00 | 433 | 240 (55.3) | 1.00 | 557 | 309 (55.5) | 1.00 |
| Male | 194 | 74 (37.9) | 1.00 (0.72-1.39) | 293 | 159 (54.4) | 0.96 (0.77-1.20) | 371 | 207 (55.8) | 1.01 (0.79-1.31) |
| **Age group (years)** |  |  |  |  |  |  |  |  |  |
| 5 – 14 | 41 | 12 (29.8) | 1.00 | 89 | 21 (24.0) | 1.00 | 134 | 37 (27.7) | 1.00 |
| 15 – 24 | 66 | 21 (31.6) | 1.09 (0.44-2.69) | 127 | 61 (47.6) | 2.87 (1.54-5.37) | 164 | 86 (52.6) | 2.89 (1.53-5.49) |
| 25 – 34 | 70 | 24 (34.6) | 1.25 (0.52-2.99) | 115 | 66 (57.4) | 4.26 (2.17-8.36) | 138 | 73 (53.0) | 2.95 (1.56-5.58) |
| 35 – 44 | 87 | 36 (42.0) | 1.71 (0.78-3.75) | 106 | 66 (62.2) | 5.21 (2.81-9.66) | 182 | 110 (60.5) | 4.01 (2.36-6.78) |
| 45 – 54 | 61 | 27 (44.3) | 1.87 (0.78-4.50) | 157 | 102 (65.3) | 5.97 (3.18-11.20) | 170 | 110 (64.8) | 4.81 (2.49-9.27) |
| 55 – 65 | 92 | 37 (40.6) | 1.61 (0.67-3.85) | 132 | 83 (62.8) | 5.36 (2.70-10.64) | 140 | 99 (70.9) | 6.38 (3.84-10.59) |
| **Race/ Skin color** |  |  |  |  |  |  |  |  |  |
| White | 188 | 61 (32.3) | 1.00 | 231 | 125 (54.1) | 1.00 | 174 | 96 (55.2) | 1.00 |
| Mixed race | 173 | 70 (40.5) | 1.43 (0.91-2.25) | 368 | 196 (53.3) | 0.97 (0.67-1.40) | 571 | 314 (54.9) | 0.99 (0.59-1.68) |
| Black | 44 | 22 (50.0) | 2.10 (1.06-4.16) | 107 | 70 (65.8) | 1.64 (1.10-2.44) | 170 | 97 (57.0) | 1.08 (0.59-1.96) |
| Other/ Ignored | 11 | 5 (46.2) | 1.80 (0.49-6.57) | 20 | 8 (39.1) | 0.55 (0.24-1.24) | 13 | 10 (72.7) | 2.17 (0.67-7.04) |
| **Schooling** |  |  |  |  |  |  |  |  |  |
| University | 232 | 73 (31.3) | 1.00 | 189 | 88 (46.7) | 1.00 | 86 | 49 (56.9) | 1.00 |
| High school | 97 | 47 (48.2) | 2.04 (1.23-3.37) | 274 | (63.0) | 1.94 (1.33-2.82) | 405 | 224 (55.3) | 0.94 (0.58-1.51) |
| Fundamental/ illiterate | 84 | 37 (44.3) | 1.74 (0.99-3.08) | 247 | 135 (54.9) | 1.39 (0.92-2.09) | 411 | 238 (58.0) | 1.05 (0.63-1.75) |
| **Monthly income (in minimum wages)** |  |  |  |  |  |  |  |  |  |
| No income / Up 2 | 179 | 78 (43.5) | 1.00 | 432 | 270 (62.5) | 1.00 | 652 | 405 (62.1) | 1.00 |
| >2-4 | 69 | 30 (44.3) | 1.03 (0.62-1.72) | 118 | 73 (62.1) | 0.99 (0.66-1.48) | 81 | 53 (64.7) | 1.12 (0.68-1.83) |
| >4 | 108 | 30 (28.0) | 0.51 (0.30-0.85) | 47 | 14 (30.2) | 0.26 (0.16-0.43) | 12 | 6 (50.0) | 0.61 (0.20-1.85) |
| **Study and/or work in the same neighborhood of residence** |  |  |  |  |  |  |  |  |  |
| Yes | 112 | 47 (41.9) | 1.00 | 242 | 129 (53.5) | 1.00 | 311 | 176 (56.5) | 1.00 |
| No | 214 | 72 (33.6) | 0.70 (0.43-1.15) | 291 | 158 (54.1) | 1.03 (0.74-1.43) | 348 | 192 (55.0) | 0.94 (0.62-1.43) |
| Neither study nor work | 88 | 38 (43.1) | 1.05 (0.59-1.90) | 185 | 108 (58.2) | 1.21 (0.80-1.82) | 265 | 149 (56.1) | 0.98 (0.72-1.35) |
| **Repellent use** |  |  |  |  |  |  |  |  |  |
| Not use | 316 | 117 (37.0) | 1.00 | 576 | 319 (55.4) | 1.00 | 746 | 419 (56.2) | 1.00 |
| Use daily | 37 | 14 (37.2) | 1.01 (0.49-2.08) | 54 | 28 (50.8) | 0.83 (0.48-1.45) | 50 | 23 (45.2) | 0.64 (0.35-1.18) |
| At least three days a week | 63 | 27 (43.1) | 1.29 (0.76-2.19) | 96 | 53 (54.6) | 0.97 (0.68-1.38) | 132 | 74 (56.4) | 1.00 (0.70-1.46) |
| **Previous DENV infection** |  |  |  |  |  |  |  |  |  |
| No | 35 | 6 (17.5) | 1.00 | 90 | 47 (52.5) | 1.00 | 119 | 68 (57.6) | 1.00 |
| Yes | 381 | 152 (39.8) | 3.11 (1.51-6.42) | 636 | 352 (55.3) | 1.21 (0.77-1.64) | 810 | 448 (55.3) | 0.91 (0.65-1.29) |
