# Supplementary material for: Seroprevalence of Dengue, Chikungunya and Zika at the epicenter of the congenital microcephaly epidemic in Northeast Brazil: A population-based survey
Source: PLoS Negl Trop Dis. 2023 Jul 3;17(7):e0011270. doi: 10.1371/journal.pntd.0011270 (PMC10348596; doi:10.1371/journal.pntd.0011270)
Supplement: S4 Table — Recife, Brazil, 2018–2019. (DOCX) [file pntd.0011270.s005.docx]

**S4 Table. Final model of the association of household and individual characteristics with ZIKV infection. Recife, Brazil, 2018-2019.**

| **Characteristics** | **Socioeconomic strata** | | | | | | |
| --- | --- | --- | --- | --- | --- | --- | --- |
|  | **High (n=414)** | | **Intermediate (n=727)** | | **Low (n=930)** | | |
|  | **Adjusted OR (95%CI)** | **p-value** | **Adjusted OR (95%CI)** | **p-value** | **Adjusted OR (95%CI)** | | **p-value** |
| **Individual** |  |  |  |  |  | |  |
| **Age group (years)** |  |  |  |  |  | |  |
| 5 – 14 | 1.00 | - | 1.00 | - | 1.00 | | - |
| 15 – 24 | 1.64 (0.50-5.36) | 0.423 | 2.45 (1.03-5.84) | 0.066 | 2.88 (1.50-5.51) | | **0.004** |
| 25 – 34 | 2.14 (0.74-6.22) | 0.173 | 3.95 (1.35-11.57) | **0.027** | 3.00 (1.57-5.73) | | **0.003** |
| 35 – 44 | 2.81 (0.95-8.28) | 0.073 | 4.74 (1.77-12.67) | **0.009** | 4.08 (2.38-6.99) | | **0.000** |
| 45 – 54 | 2.98 (0.96-9.26) | 0.070 | 5.33 (1.83-15.48) | **0.010** | 5.01 (2.58-9.74) | | **0.000** |
| 55 – 65 | 2.52 (0.87-7.31) | 0.103 | 5.07 (1.70-15.12) | **0.013** | 6.58 (3.93-11.00) | | **0.000** |
| **Schooling (age ≥13 years)** |  |  |  |  |  | |  |
| University | 1.00 | - | 1.00 | - |  | |  |
| Intermediate | 1.62 (0.97-2.68) | 0.075 | 1.36 (0.86-2.14) | 0.215 |  | |  |
| Fundamental/illiterate | 1.50 (0.73-3.08) | 0.278 | 1.20 (0.70-2.08) | 0.524 |  | |  |
| **Monthly income (in minimum wages)** |  |  |  |  |  | |  |
| No income/Up 2 |  |  | 1.00 | - |  | |  |
| >2-4 |  |  | 1.27 (0.80-2.00) | 0.329 |  | |  |
| >4 |  |  | 0.43 (0.20-0.89) | **0.042** |  | |  |
| **Previous DENV exposure** |  |  |  |  |  | |  |
| No | 1,00 | - |  |  |  |  | |
| Yes | 3.08 (1.42-6.68) | **0.009** |  |  |  |  | |
| **Related to the household** |  |  |  |  |  |  | |
| **Type of household** |  |  |  |  |  |  | |
| Apartment | 1.00 | - | 1.00 | - | 1.00 | - | |
| House | 2.81 (1.76-4.48) | **0.000** | 2.17 (1.32-3.57) | **0.010** | 2.42 (1.28-4.57) | **0.012** | |
| **Monthly income (in minimum wages)** |  |  |  |  |  |  | |
| No income/ Up 2 |  |  | 1.00 | - |  |  | |
| >2-4 |  |  | 0.69 (0.47-1.02) | 0.088 |  |  | |
| >4 |  |  | 0.76 (0.41-1.41) | 0.399 |  |  | |
