# Supplementary material for: Seroprevalence of Dengue, Chikungunya and Zika at the epicenter of the congenital microcephaly epidemic in Northeast Brazil: A population-based survey
Source: PLoS Negl Trop Dis. 2023 Jul 3;17(7):e0011270. doi: 10.1371/journal.pntd.0011270 (PMC10348596; doi:10.1371/journal.pntd.0011270)
Supplement: S5 Table — Recife, Brazil, 2018–2019. (DOCX) [file pntd.0011270.s006.docx]

**S5 Table. Crude analysis of the association between household characteristics and CHIKV infection. Recife, Brazil, 2018-2019.**

| **Characteristics** | **Socioeconomic strata** | | | | | | | | |
| --- | --- | --- | --- | --- | --- | --- | --- | --- | --- |
|  | **High** | | | **Intermediate** | | | **Low** | | |
|  | **Total** | **Positive** | **OR (IC95%)** | **Total** | **Positive** | **OR (IC95%)** | **Total** | **Positive** | **OR (IC95%)** |
|  |  | **n (%)** |  |  | **n (%)** |  |  | **n (%)** |  |
| **Number of residents in the household** |  |  |  |  |  |  |  |  |  |
| Up 2 | 103 | 23 (22.7) | 1.00 | 150 | 56 (37.5) | 1.00 | 115 | 48 (41.7) | 1.00 |
| >2-4 | 213 | 49 (22.8) | 1.01 (0.65 - 1.55) | 358 | 140 (39.1) | 1.07 (0.70-1.64) | 437 | 156 (35.6) | 0.77 (0.72-1.41) |
| >4 | 100 | 29 (29.6) | 1.43 (0.74 - 2.79) | 218 | 101 (46.1) | 1.43 (0.88-2.32) | 376 | 168 (44.6) | 1.13 (0.57-2.21) |
| **Type of household** |  |  |  |  |  |  |  |  |  |
| Apartment | 256 | 39 (15.2) | 1.00 | 124 | 23 (18.7) | 1.00 | 30 | 0 (0.0) | 1.00 |
| House | 159 | 62 (39.3) | 3.62 (1.92 - 6.83) | 598 | 272 (45.5) | 3.62 (2.12 - 6.20) | 898 | 371 (41.3) | - |
| **Waste destination** |  |  |  |  |  |  |  |  |  |
| Public network | 290 | 68 (23.6) | 1.00 | 434 | 184 (42.3) | 1.00 | 341 | 147 (43.2) | 1.00 |
| Other destination | 84 | 28 (33.0) | 1.60 (0.86 - 2.96) | 269 | 104 (38.7) | 0.86 (0.63-1.19) | 575 | 222 (38.5) | 0.83 (0.54-1.26) |
| **Water supply** |  |  |  |  |  |  |  |  |  |
| Public network | 278 | 71 (25.5) | 1.00 | 662 | 278 (42.0) | 1.00 | 831 | 328 (39.5) | 1.00 |
| Well/other sources | 135 | 30 (22.4) | 0.84 (0.42 - 1.70) | 60 | 17 (28.4) | 0.55 (0.27-1.12) | 90 | 37 (41.3) | 1.08 (0.65-1.78) |
| **Irregularity of water supply** |  |  |  |  |  |  |  |  |  |
| No | 364 | 79 (21.7) | 1.00 | 595 | 234 (39.4) | 1.00 | 532 | 204 (38.3) | 1.00 |
| Yes | 51 | 23 (44.1) | 2.85 (1.21-6.73) | 127 | 61 (47.9) | 1.42 (0.86-2.33) | 394 | 168 (42.6) | 1.19 (0.84-1.71) |
| **Garbage destination** |  |  |  |  |  |  |  |  |  |
| Home collection | 412 | 101 (24.4) | 1.00 | 650 | 270 (41.5) | 1.00 | 850 | 327 (38.5) | 1.00 |
| Other destination | 3 | 1 (33.3) | 1.03 (0.20-5.21) | 71 | 25 (35.0) | 0.76 (0.46-1.24) | 78 | 44 (56.9) | 2.12 (1.12-4.01) |
| **Monthly income (in minimum wages)** |  |  |  |  |  |  |  |  |  |
| No income / Up to 2 | 125 | 49 (38.9) |  | 418 | 184 (43.9) | 1.00 | 751 | 325 (43.2) | 1.00 |
| >2-4 | 91 | 19 (21.0) | 0.42 (0.20-0.87) | 197 | 79 (40.3) | 0.86 (0.59 - 1.26) | 148 | 43 (29.0) | 0.54 (0.39-0.73) |
| >4 | 196 | 33 (16.8) | 0.32 (0.17-0.59) | 101 | 30 (29.8) | 0.54 (0.34 - 0.88) | 17 | 1 (7.1) | 0.10 (0.02-0.48) |
| **Characteristics of the head of the family** |  |  |  |  |  |  |  |  |  |
| **Schooling** |  |  |  |  |  |  |  |  |  |
| University | 253 | 37 (14.7) | 1.00 | 180 | 51 (28.2) | 1.00 | 42 | 10 (22.9) | 1.00 |
| High school | 85 | 31 (36.7) | 3.36 (1.96 - 5.76) | 256 | 118 (46.3) | 2.20 (1.46 - 3.31) | 346 | 147 (42.6) | 2.50 (0.64-9.73) |
| Fundamental/illiterate | 75 | 33 (44.2) | 4.58 (2.24 - 9.40) | 283 | 124 (43.7) | 1.98 (1.28 - 3.05) | 525 | 208 (39.7) | 2.22 (0.53-9.26) |
| **Race/ Skin color** |  |  |  |  |  |  |  |  |  |
| White | 194 | 42 (21.9) | 1.00 | 243 | 105 (43.2) | 1.00 | 152 | 56 (37.0) | 1.00 |
| Mixed race | 163 | 41 (25.0) | 1.19 (0.76-1.87) | 361 | 147 (40.7) | 0.90 (0.62-1.32) | 585 | 224 (38.3) | 1.06 (0.66-1.68) |
| Black | 37 | 10 (25.6) | 1.23 (0.59-2.57) | 100 | 37 (36.6) | 0.76 (0.46-1.25) | 171 | 85 (49.7) | 1.68 (0.97-2.90) |
| Other/ Ignored | 22 | 9 (40.0) | 2.38 (0.82-6.94) | 22 | 8 (36.0) | 0.74 (0.31-1.77) | 20 | 6 (29.4) | 0.71 (0.23-2.15) |
