# Supplementary material for: Seroprevalence of Dengue, Chikungunya and Zika at the epicenter of the congenital microcephaly epidemic in Northeast Brazil: A population-based survey
Source: PLoS Negl Trop Dis. 2023 Jul 3;17(7):e0011270. doi: 10.1371/journal.pntd.0011270 (PMC10348596; doi:10.1371/journal.pntd.0011270)
Supplement: S6 Table — Recife, Brazil, 2018–2019. (DOCX) [file pntd.0011270.s007.docx]

**S6 Table. Crude analysis of the association between individual characteristics and CHIKV infection. Recife, Brazil, 2018-2019.**

| **Characteristics** | **Socioeconomic strata** | | | | | | | | |
| --- | --- | --- | --- | --- | --- | --- | --- | --- | --- |
|  | **High** | | | **Intermediate** | | | **Deprived** | | |
|  | **Total** | **Positive** | **OR (IC95%)** | **Total** | **Positive** | **OR (IC95%)** | **Total** | **Positive** | **OR (IC95%)** |
|  |  | **n (%)** |  |  | **n (%)** |  |  | **n (%)** |  |
| **Gender** |  |  |  |  |  |  |  |  |  |
| Female | 222 | 60 (27.0) | 1.00 | 433 | 179 (41.4) | 1.00 | 557 | 225 (40.4) | 1.00 |
| Male | 194 | 42 (21.4) | 0.74 (0.50 - 1.10) | 293 | 118 (40.1) | 0.95 (0.74-1.22) | 371 | 146 (39.4) | 0.96 (0.72-1.28) |
| **Age group (years)** |  |  |  |  |  |  |  |  |  |
| 5 – 14 | 41 | 11 (27.7) | 1.00 | 89 | 33 (37.0) | 1.00 | 134 | 41 (30.4) | 1.00 |
| 15 – 24 | 66 | 12 (18.4) | 0.59 (0.25-1.38) | 127 | 48 (37.8) | 1.03 (0.66-1.63) | 164 | 73 (44.5) | 1.84 (1.09-3.10) |
| 25 – 34 | 70 | 16 (23.5) | 0.80 (0.35-1.82) | 115 | 45 (38.8) | 1.08 (0.71-1.63) | 138 | 60 (43.5) | 1.77 (1.14-2.73) |
| 35 – 44 | 86 | 21 (24.0) | 0.83 (0.37-1.87) | 106 | 45 (42.0) | 1.23 (0.76-2.01) | 182 | 67 (36.8) | 1.34 (0.85-2.10) |
| 45 – 54 | 61 | 20 (32.9) | 1.28 (0.70-2.36) | 157 | 64 (40.9) | 1.18 (0.74-1.89) | 170 | 66 (38.7) | 1.45 (0.82-2.58) |
| 55 – 65 | 92 | 21 (22.6) | 0.77 (0.39-1.50) | 132 | 62 (47.3) | 1.53 (0.87-2.70) | 140 | 65 (46.2) | 1.97 (1.09-3.56) |
| **Raçe/Skin color** |  |  |  |  |  |  |  |  |  |
| White | 188 | 39 (20.7) | 1.00 | 231 | 96 (41.7) | 1.00 | 174 | 67 (38.6) | 1.00 |
| Mixed race | 173 | 48 (27.5) | 1.45 (0.89-2.35) | 368 | 150 (40.7) | 0.96 (0.70-1.30) | 571 | 218 (38.2) | 0.98 (0.71-1.36) |
| Black | 44 | 11 (26.0) | 1.34 (0.79-2.28) | 107 | 45 (41.7) | 1.00 (0.65-1.54) | 170 | 84 (49.3) | 1.55 (0.91-2.63) |
| Others/ Ignored | 11 | 3 (30.8) | 1.70 (0.44-6.51) | 20 | 6 (30.4) | 0.61 (0.20-1.92) | 13 | 2 (18.2) | 0.35 (0.05-2.56) |
| **Schooling** |  |  |  |  |  |  |  |  |  |
| University | 232 | 36 (15.3) | 1.00 | 189 | 59 (31.1) | 1.00 | 86 | 22 (25.0) | 1.00 |
| High school | 97 | 32 (33.0) | 2.73 (1.70-4.40) | 274 | 128 (46.8) | 1.94 (1.38-2.73) | 405 | 170 (42.0) | 2.17 (1.43-3.31) |
| Fundamental/ illiterate | 84 | 34 (40.2) | 3.72 (1.97-7.04) | 247 | 104 (42.2) | 1.62 (1.16-2.26) | 411 | 172 (42.0) | 2.17 (1.31-3.60) |
| **Monthly income (in minimum wages)** |  |  |  |  |  |  |  |  |  |
| No income / Up to 2 | 179 | 58 (32.4) | 1.00 | 432 | 193 (44.7) | 1.00 | 652 | 280 (43.0) | 1.00 |
| >2-4 | 69 | 13 (19.0) | 0.49 (0.26-0.92) | 118 | 48 (40.9) | 0.86 (0.57-1.29) | 81 | 23 (27.9) | 0.51 (0.28-0.94) |
| >4 | 108 | 16 (14.4) | 0.35 (0.18-0.68) | 47 | 11 (22.6) | 0.36 (0.20-0.65) | 12 | 2 (20.0) | 0.33 (0.07-1.64) |
| **Study and/or work in the same neighborhood of residence** |  |  |  |  |  |  |  |  |  |
| Yes | 112 | 29 (25.6) | 1.00 | 241 | 110 (45.4) | 1.00 | 311 | 141 (45.4) | 1.00 |
| No | 214 | 43 (20.2) | 0.74 (0.48-1.15) | 291 | 104 (35.8) | 0.67 (0.47-0.97) | 349 | 122 (35.1) | 0.65 (0.49-0.87) |
| Neither study nor work | 88 | 29 (33.3) | 1.46 (0.81-2.60) | 185 | 80 (43.3) | 0.92 (0.64-1.32) | 265 | 107 (40.3) | 0.81 (0.59-1.12) |
| **Use of repellent** |  |  |  |  |  |  |  |  |  |
| Not use | 316 | 78 (24.7) | 1.00 | 576 | 239 (41.5) | 1.00 | 746 | 302 (40.4) | 1.00 |
| Use daily | 37 | 3 (7.0) | 0.23 (0.05-1.02) | 54 | 21 (39.3) | 0.92 (0.51-1.65) | 50 | 25 (50.0) | 1.47 (0.81-2.68) |
| At least three days a week | 63 | 21 (33.3) | 1.53 (0.83-2.82) | 96 | 37 (38.0) | 0.86 (0.61-1.22) | 132 | 44 (33.6) | 0.75 (0.52-1.06) |
| **Previous DENV infection** |  |  |  |  |  |  |  |  |  |
| No | 35 | 9 (25.0) | 1.00 | 90 | 27 (29.7) | 1.00 | 118 | 42 (35.4) | 1.00 |
| Yes | 381 | 93 (24.3) | 0.96 (0.46-2.04) | 636 | 270 (42.4) | 1.75 (1.16-2.63) | 810 | 329 (40.7) | 1.25 (0.84-1.88) |
