# Supplementary material for: Seroprevalence of Dengue, Chikungunya and Zika at the epicenter of the congenital microcephaly epidemic in Northeast Brazil: A population-based survey
Source: PLoS Negl Trop Dis. 2023 Jul 3;17(7):e0011270. doi: 10.1371/journal.pntd.0011270 (PMC10348596; doi:10.1371/journal.pntd.0011270)
Supplement: S7 Table — Recife, Brazil, 2018–2019. (DOCX) [file pntd.0011270.s008.docx]

**S7 Table. Final model of the association of household and individual characteristics with CHIKV infection. Recife, Brazil, 2018-2019.**

| **Characteristics** | **Socioeconomic strata** | | | | | | | | |
| --- | --- | --- | --- | --- | --- | --- | --- | --- | --- |
|  | **High (n=416)** | | | **Intermediate (n=726)** | | | **Deprived (n=928)** | | |
|  | **Adjusted OR (95%CI)** | **p-value** | | **Adjusted OR (95%CI)** | **p-value** | | **Adjusted OR (95%CI)** | | **p-value** |
| **Individual** |  |  | |  |  | |  | |  |
| **Age group (years)** |  |  | |  |  | |  | |  |
| 5 – 14 |  |  | |  |  | | 1.00 | | - |
| 15 – 24 |  |  | |  |  | | 2.00 (1.08-3.72) | | **0.043** |
| 25 – 34 |  |  | |  |  | | 1.95 (1.09-3.46) | | **0.037** |
| 35 – 44 |  |  | |  |  | | 1.50 (0.91-2.48) | | 0.134 |
| 45 – 54 |  |  | |  |  | | 1.54 (0.77-3.05) | | 0.237 |
| 55 – 65 |  |  | |  |  | | 2.10 (1.09-4.06) | | **0.042** |
| **Schooling (age ≥13 years)** |  |  | |  |  | |  | |  |
| University | 1.00 | - | | 1.00 | - | | 1.00 | | - |
| High school | 1.58 (0.88-2.84) | 0.140 | | 1.37 (0.94-2.00) | 0.123 | | 1.99 (1.31-3.02) | | **0.005** |
| Fundamental/ illiterate | 1.77 (1.05-2.99) | **0.043** | | 1.16 (0.82-1.63) | 0.419 | | 2.11 (1.29-3.45) | | **0.008** |
| **Monthly income (in minimum wages)** |  |  | |  |  | |  | |  |
| No income /Up to 2 |  |  | | 1.00 | - | |  | |  |
| >2-4 |  |  | | 1.02 (0.65-1.59) | 0.951 | |  | |  |
| >4 |  |  | | 0.61 (0.33-1.15) | 0.145 | |  | |  |
| **Use of repellent** |  |  | |  |  | |  | |  |
| Do not use | 1.00 | - | |  |  | |  | |  |
| Use daily | 0.22 (0.05-0.87) | **0.041** | |  |  | |  | |  |
| At least three days a week | 1.91 (0.98-3.76) | 0.071 | |  |  | |  | |  |
| **Previous DENV infection** |  |  | |  |  | |  | |  |
| No |  |  | | 1,00 | - | |  | |  |
| Yes |  |  | | 1.97 (1.28-3.02) | **0.006** | |  | |  |
| **Related to the household** |  |  | |  |  | |  | |  |
| **Type of household** |  |  | |  |  | |  | |  |
| Apartment | 1.00 | - | | 1.00 | - | |  | |  |
| House | 2.64 (1.34-5.17) | **0.010** | | 3.23 (1.80-5.80) | **0.001** | |  | |  |
| **Waste destination** |  |  | |  |  | |  | |  |
| Public network | 1.00 | | - |  | |  |  |  | |
| Other destination | 2.50 (1.39-4.52) | | **0.006** |  | |  |  |  | |
| **Monthly income (in minimum wages)** |  | |  |  | |  |  |  | |
| No income /Up to 2 |  | |  |  | |  | 1.00 | - | |
| >2-4 |  | |  |  | |  | 0.59 (0.43-0.80) | **0.004** | |
| >4 |  | |  |  | |  | 0.27 (0.07-1.08) | 0.082 | |
| **Schooling** |  | |  |  | |  |  |  | |
| University | 1.00 | | - |  | |  |  |  | |
| High school | 2.37 (1.20-4.68) | | **0.021** |  | |  |  |  | |
| Fundamental/illiterate | 1.92 (0.86-4.33) | | 0.126 |  | |  |  |  | |
