# Supplementary material for: Seroprevalence of Dengue, Chikungunya and Zika at the epicenter of the congenital microcephaly epidemic in Northeast Brazil: A population-based survey
Source: PLoS Negl Trop Dis. 2023 Jul 3;17(7):e0011270. doi: 10.1371/journal.pntd.0011270 (PMC10348596; doi:10.1371/journal.pntd.0011270)
Supplement: S1 Additional methods — (DOCX) [file pntd.0011270.s009.docx]

**S1 Additional methods.**

**ZIKV NS1 IgG3 assay**

The detection of recent ZIKV infection (with seroconversion within 4 months prior blood collection) was performed through de measurement of anti-ZIKV IgG3 antibodies using a previously described protocol (1). Briefly, half area 96-well plates (Corning, USA) were coated overnight at 4ºC with ZIKV NS1 (Native Antigen, UK) at 2.3 μg/mL in carbonate buffer (Thermo Fisher, USA). Plates were blocked with skimmed milk (Bio-Rad) at 5% (w/v) in PBS-T buffer [1X PBS with 0.05% (v/v) Tween 20] for 15 min at room temperature (RT). Serum samples and assay controls were diluted at 1:50 in assay buffer [5% (w/v) skimmed milk in PBS-T], added to the wells in duplicate, and incubated for 2 hours at RT. Sera from recently infected individuals (collected 20-30 days post onset of symptoms) were pooled and used as ZIKV IgG3 positive control. Pooled sera from recent DENV infection (collected 20-30 days post onset of symptoms) and pooled sera from healthy individuals naïve to both ZIKV and DENV were used as DENV IgG3 positive control and ZIKV IgG3 negative controls, respectively. After 5 washes with PBS-T, horseradish peroxidase (HEP)-conjugated mouse monoclonal antibody anti-human IgG3 (Invitrogen, USA) was added to the wells at a 1:500 dilution and incubated for 1 hour at RT. After another washing cycle, the reaction was developed by the addition of tetramethylbenzidine TMB-KPL substrate (KPL, USA) to the wells followed by incubation for 30 min at RT. The reaction was stopped with 1N HCl (Sigma, USA). Optical densities at a wavelength of 450nm (OD450nm) were read using a microplate spectrophotometer (BioTek, USA). Blank correction was performed for each well prior analysis and the controls were run in each plate to determine the reproducibility of the assay.

Data analysis was performed by calculating the ZIKV ratio by dividing the OD450nm of the samples by the average OD450nm of the DENV recent infection control. ROC curve analysis was used to evaluate the sensitivity and specificity at all possible cut-off points and to choose the cut-off that would maximize the sensitivity while maintaining specificity > 95%. Therefore, the cut-off was set to 1.14. A sample was defined as eligible for analysis if the average ratio of a sample was above the cut-off value (antigen-specific IgG3 positive sample) and coefficient of variation [(standard deviation of the replicates/average of replicates) x 100] of replicates was below 20%.

**DENV NS1 total IgG assay**

Detection of anti-DENV NS1 total IgG was performed using an indirect ELISA previously described (2) with modifications. Briefly, half area 96-well plates (Corning, USA) were coated overnight at 4ºC with DENV 1-4 NS1 (pooled at equivocar ratio; Native Antigen, UK) at 1 μg/mL in carbonate buffer (Thermo Fisher, USA). Plates were blocked or 15 min at RT. Serum samples and assay controls were diluted at 1:50 in assay buffer, added to the wells in duplicate, and incubated for 2 hours at RT. Pooled sera from DENV IgG positive and from DENV naïve individuals were used as DENV total IgG positive and negative controls, respectively, and added to each plate. Plates were washed 5 times with PBS-T and incubated with HRP-conjugated anti-human IgG antibody (Jackson Immunoresearch, USA), for 1 hour at RT. Plates were then washed and the reaction was developed as described for the ZIKV NS1 IgG3 assay. Blank correction was performed for each well prior analysis.

Data analysis was performed by calculating the DENV ration by dividing the OD450nm of the samples by the average OD450nm of the negative control. ROC curve analysis was used to evaluate the sensitivity and specificity at all possible cut-off points and to chose the cut-off that would maximize the sensitivity while maintaining specificity > 95% (3.62). A sample was defined as eligible for analysis if the OD450nm of a sample was above the cut-off value and coefficient of variation of replicates was below 20%.

**Plaque reduction neutralization test (PRNT)**

A subset of randomly selected 156 serum samples were tested for ZIKV-specific neutralizing antibodies through PRNT assay aiming to validate the serological ELISA tests (presence of IgM and IgG antibodies by ELISA). ZIKV-specific neutralizing antibodies were initially assessed by PRNT, following a modified protocol described in detail elsewhere (3). Briefly, neutralization was assessed in a monolayer of Vero cells infected with the ZIKV local strain (BR-PE243/2015). The cut-off for PRNT positivity was defined based on a 50% reduction in plaque counts (PRNT50), and ZIKV-specific antibody titers were estimated using a four-parameter non-linear regression. Samples were considered positive when neutralizing antibody levels were ≥1:100 for ZIKV.
