# Supplementary material for: Seroprevalence of Dengue, Chikungunya and Zika at the epicenter of the congenital microcephaly epidemic in Northeast Brazil: A population-based survey
Source: PLoS Negl Trop Dis. 2023 Jul 3;17(7):e0011270. doi: 10.1371/journal.pntd.0011270 (PMC10348596; doi:10.1371/journal.pntd.0011270)
Supplement: S2 Additional methods — (DOCX) [file pntd.0011270.s010.docx]

**S2 Additional methods.**

Packages for Prevalence Data Analysis using R Programme

#########################

## DATA ANALYSIS ##

#########################

##################################################

## Packages (libraries) for Data Analysis in R ##

##################################################

require(foreign)

library(prevalence)

require(survey)

require(prevalence)

library(MASS)

library(mgcv)

library(dplyr)

library(ggplot2)

library(ggnewscale)

library(ggplot2)

library(tidyverse)

library(gridExtra)

library(rmarkdown)

library(visibly)

library(ggplot2)

library(visreg)

require(ggthemes)

require(sjmisc)

require(cowplot)

################

# READING DATA #

################

setwd("C:/Users/cflun/downloads/Cynthia Braga/Artigo 1 - Prevalência/dados")

dados1<-read.spss("dados.sav")

dados2<-read.csv("dados.csv", sep=";", dec=",")

#options(survey.lonely.psu = "adjust")

options(survey.lonely.psu = "certainty")

svy<-svydesign(id=~cod_setor+cod_dom, fpc=~fpc1+fpc2, strata=~cluster,weights=~peso, data=dados1, nest=TRUE)

summary(svy)

#options(survey.lonely.psu = "adjust")

options(survey.lonely.psu = "certainty")

svy<-svydesign(id=~ï..cod_setor+cod_dom, fpc=~fpc1+fpc2, strata=~cluster,weights=~peso, data=dados2, nest=TRUE)

summary(svy)

cluster1<-subset(dados2, cluster==1)

cluster2<-subset(dados2, cluster==2)

cluster3<-subset(dados2, cluster==3)

options(survey.lonely.psu = "certainty")

svy.cluster1<-svydesign(id=~ï..cod_setor+cod_dom, fpc=~fpc1+fpc2, weights=~peso, data=cluster1, nest=TRUE)

svy.cluster2<-svydesign(id=~ï..cod_setor+cod_dom, fpc=~fpc1+fpc2, weights=~peso, data=cluster2, nest=TRUE)

svy.cluster3<-svydesign(id=~ï..cod_setor+cod_dom, fpc=~fpc1+fpc2, weights=~peso, data=cluster3, nest=TRUE)

sexoF<-subset(dados2, sex==0)

sexoM<-subset(dados2, sex==1)

options(survey.lonely.psu = "certainty")

svy.sexoF<-svydesign(id=~ï..cod_setor+cod_dom, fpc=~fpc1+fpc2, strata=~cluster, weights=~peso, data=sexoF, nest=TRUE)

svy.sexoM<-svydesign(id=~ï..cod_setor+cod_dom, fpc=~fpc1+fpc2, strata=~cluster, weights=~peso, data=sexoM, nest=TRUE)

idgrupo1<-subset(dados2, idgrupo==1)

idgrupo2<-subset(dados2, idgrupo==2)

idgrupo3<-subset(dados2, idgrupo==3)

idgrupo4<-subset(dados2, idgrupo==4)

idgrupo5<-subset(dados2, idgrupo==5)

idgrupo6<-subset(dados2, idgrupo==6)

svy.idgrupo1<-svydesign(id=~ï..cod_setor+cod_dom, fpc=~fpc1+fpc2, strata=~cluster, weights=~peso, data=idgrupo1, nest=TRUE)

svy.idgrupo2<-svydesign(id=~ï..cod_setor+cod_dom, fpc=~fpc1+fpc2, strata=~cluster, weights=~peso, data=idgrupo2, nest=TRUE)

svy.idgrupo3<-svydesign(id=~ï..cod_setor+cod_dom, fpc=~fpc1+fpc2, strata=~cluster, weights=~peso, data=idgrupo3, nest=TRUE)

svy.idgrupo4<-svydesign(id=~ï..cod_setor+cod_dom, fpc=~fpc1+fpc2, strata=~cluster, weights=~peso, data=idgrupo4, nest=TRUE)

svy.idgrupo5<-svydesign(id=~ï..cod_setor+cod_dom, fpc=~fpc1+fpc2, strata=~cluster, weights=~peso, data=idgrupo5, nest=TRUE)

svy.idgrupo6<-svydesign(id=~ï..cod_setor+cod_dom, fpc=~fpc1+fpc2, strata=~cluster, weights=~peso, data=idgrupo6, nest=TRUE)

idgrupo3.1<-subset(dados2, idgrupo3==1)

idgrupo3.2<-subset(dados2, idgrupo3==2)

idgrupo3.3<-subset(dados2, idgrupo3==3)

svy.idgrupo3.1<-svydesign(id=~ï..cod_setor+cod_dom, fpc=~fpc1+fpc2, strata=~cluster, weights=~peso, data=idgrupo3.1, nest=TRUE)

svy.idgrupo3.2<-svydesign(id=~ï..cod_setor+cod_dom, fpc=~fpc1+fpc2, strata=~cluster, weights=~peso, data=idgrupo3.2, nest=TRUE)

svy.idgrupo3.3<-svydesign(id=~ï..cod_setor+cod_dom, fpc=~fpc1+fpc2, strata=~cluster, weights=~peso, data=idgrupo3.3, nest=TRUE)

##############

# TABLE 1 #

##############

# SAMPLE

round(svytable(~cluster, design = svy), digits=0)

round(svytable(~sex, design = svy), digits=0)

round(svytable(~idgrupo, design = svy), digits=0)

round(svytable(~idgrupo3, design = svy), digits=0)

##############

### DENGUE ###

##############

### Total

round(svytable(~igg_denv, design = svy), digits=0)

svymean(~igg_denv, svy)

confint(svymean(~igg_denv, svy))

### Cluster

round(svytable(~igg_denv+cluster, design = svy), digits=0)

round(prop.table(svytable(~igg_denv+cluster, design = svy), margin = 2), digits = 3)

round(svyby(formula = ~igg_denv, by = ~cluster, design = svy, FUN = svymean, na.rm=TRUE, vartype=c("se","ci")), digits = 3)

### Sex

round(svytable(~igg_denv+sex, design = svy), digits=0)

round(prop.table(svytable(~igg_denv+sex, design = svy), margin = 2), digits = 3)

round(svyby(formula = ~igg_denv, by = ~sex, design = svy, FUN = svymean, na.rm=TRUE, vartype=c("se","ci")), digits = 3)

### AGE GROUPS

round(svytable(~igg_denv+idgrupo, design = svy), digits=0)

round(prop.table(svytable(~igg_denv+idgrupo, design = svy), margin = 2), digits = 3)

round(svyby(formula = ~igg_denv, by = ~idgrupo, design = svy, FUN = svymean, na.rm=TRUE, vartype=c("se","ci")), digits = 3)

###############

### Zika ###

###############

###########

## IGG ##

###########

### Total

round(svytable(~igg_zikv, design = svy), digits=0)

svymean(~igg_zikv, svy, na.rm=TRUE)

confint(svymean(~igg_zikv, svy, na.rm=TRUE))

### Cluster

round(svytable(~igg_zikv+cluster, design = svy), digits=0)

round(prop.table(svytable(~igg_zikv+cluster, design = svy), margin = 2), digits = 3)

round(svyby(formula = ~igg_zikv, by = ~cluster, design = svy, FUN = svymean, na.rm=TRUE, vartype=c("se","ci")), digits = 3)

### Sex

round(svytable(~igg_zikv+sex, design = svy), digits=0)

round(prop.table(svytable(~igg_zikv+sex, design = svy), margin = 2), digits = 3)

round(svyby(formula = ~igg_zikv, by = ~sex, design = svy, FUN = svymean, na.rm=TRUE, vartype=c("se","ci")), digits = 3)

### AGE GROUPS

round(svytable(~igg_zikv+idgrupo, design = svy), digits=0)

round(prop.table(svytable(~igg_zikv+idgrupo, design = svy), margin = 2), digits = 3)

round(svyby(formula = ~igg_zikv, by = ~idgrupo, design = svy, FUN = svymean, na.rm=TRUE, vartype=c("se","ci")), digits = 3)

###########

## IGG3 ##

###########

### Total

round(svytable(~igg3_zikv, design = svy), digits=0)

svymean(~igg3_zikv, svy, na.rm=TRUE)

confint(svymean(~igg3_zikv, svy, na.rm=TRUE))

### Cluster

round(svytable(~igg3_zikv+cluster, design = svy), digits=0)

round(prop.table(svytable(~igg3_zikv+cluster, design = svy), margin = 2), digits = 3)

round(svyby(formula = ~igg3_zikv, by = ~cluster, design = svy, FUN = svymean, na.rm=TRUE, vartype=c("se","ci")), digits = 3)

### Sex

round(svytable(~igg3_zikv+sex, design = svy), digits=0)

round(prop.table(svytable(~igg3_zikv+sex, design = svy), margin = 2), digits = 3)

round(svyby(formula = ~igg3_zikv, by = ~sex, design = svy, FUN = svymean, na.rm=TRUE, vartype=c("se","ci")), digits = 3)

### AGE GROUPS

round(svytable(~igg3_zikv+idgrupo, design = svy), digits=0)

round(prop.table(svytable(~igg3_zikv+idgrupo, design = svy), margin = 2), digits = 3)

round(svyby(formula = ~igg3_zikv, by = ~idgrupo, design = svy, FUN = svymean, na.rm=TRUE, vartype=c("se","ci")), digits = 3)

####################

## IGG_ZIKV_TOTAL ##

####################

### Total

round(svytable(~igg_zikv_total, design = svy), digits=0)

round(svymean(~igg_zikv_total, svy, na.rm=TRUE), digits=3)

round(confint(svymean(~igg_zikv_total, svy, na.rm=TRUE)), digits=3)

### Cluster

round(svytable(~igg_zikv_total+cluster, design = svy), digits=0)

round(prop.table(svytable(~igg_zikv_total+cluster, design = svy), margin = 2), digits = 3)

round(svyby(formula = ~igg_zikv_total, by = ~cluster, design = svy, FUN = svymean, na.rm=TRUE, vartype=c("se","ci")), digits = 3)

### Sex

round(svytable(~igg_zikv_total+sex, design = svy), digits=0)

round(prop.table(svytable(~igg_zikv_total+sex, design = svy), margin = 2), digits = 3)

round(svyby(formula = ~igg_zikv_total, by = ~sex, design = svy, FUN = svymean, na.rm=TRUE, vartype=c("se","ci")), digits = 3)

### AGE GROUPS

round(svytable(~igg_zikv_total+idgrupo, design = svy), digits=0)

round(prop.table(svytable(~igg_zikv_total+idgrupo, design = svy), margin = 2), digits = 3)

round(svyby(formula = ~igg_zikv_total, by = ~idgrupo, design = svy, FUN = svymean, na.rm=TRUE, vartype=c("se","ci")), digits = 3)

#############################################################

## CORRECTION OF PREVALENCE BY SENSITIVITY AND SPECIFICITY ##

#############################################################

### Total

round(svytable(~igg_zikv, design = svy), digits=0)

truePrev(1044, sum(1027,1044), SE = ~dunif(0.75, 1.00), SP = ~dunif(0.60, 0.80))

round(svytable(~igg3_zikv, design = svy), digits=0)

truePrev(104, sum(104,1966), SE = ~dunif(0.60, 0.95), SP = ~dunif(0.88, 0.97))

round(svytable(~igg_zikv_total, design = svy), digits=0)

truePrev(1072, sum(1072,998), SE = ~dunif(0.75, 1.00), SP = ~dunif(0.60, 0.80))

### Cluster

round(svytable(~igg_zikv+cluster, design = svy), digits=0)

round(prop.table(svytable(~igg_zikv+cluster, design = svy), margin = 2), digits = 3)

truePrev(148, sum(148,265), SE = ~dunif(0.75, 1.00), SP = ~dunif(0.60, 0.80), prior=c(6,4))

truePrev(386, sum(386,342), SE = ~dunif(0.75, 1.00), SP = ~dunif(0.60, 0.80), prior=c(6,4))

truePrev(510, sum(510,420), SE = ~dunif(0.75, 1.00), SP = ~dunif(0.60, 0.80), prior=c(6,4))

round(svytable(~igg3_zikv+cluster, design = svy), digits=0)

round(prop.table(svytable(~igg3_zikv+cluster, design = svy), margin = 2), digits = 3)

truePrev(25, sum(25,389), SE = ~dunif(0.60, 0.95), SP = ~dunif(0.88, 0.97), prior=c(6,4))

truePrev(41, sum(41,686), SE = ~dunif(0.60, 0.95), SP = ~dunif(0.88, 0.97), prior=c(6,4))

truePrev(38, sum(38,891), SE = ~dunif(0.60, 0.95), SP = ~dunif(0.88, 0.97), prior=c(6,4))

round(svytable(~igg_zikv_total+cluster, design = svy), digits=0)

round(prop.table(svytable(~igg_zikv_total+cluster, design = svy), margin = 2), digits = 3)

truePrev(157, sum(157,257), SE = ~dunif(0.75, 1.00), SP = ~dunif(0.60, 0.80), prior=c(6,4))

truePrev(400, sum(400,328), SE = ~dunif(0.75, 1.00), SP = ~dunif(0.60, 0.80), prior=c(6,4))

truePrev(516, sum(516,414), SE = ~dunif(0.75, 1.00), SP = ~dunif(0.60, 0.80), prior=c(6,4))

### Sex

round(svytable(~igg_zikv+sex, design = svy), digits=0)

round(prop.table(svytable(~igg_zikv+sex, design = svy), margin = 2), digits = 3)

truePrev(618, sum(618,594), SE = ~dunif(0.75, 1.00), SP = ~dunif(0.60, 0.80))

truePrev(426, sum(426,433), SE = ~dunif(0.75, 1.00), SP = ~dunif(0.60, 0.80))

round(svytable(~igg3_zikv+sex, design = svy), digits=0)

round(prop.table(svytable(~igg3_zikv+sex, design = svy), margin = 2), digits = 3)

truePrev(61, sum(61,1151), SE = ~dunif(0.60, 0.95), SP = ~dunif(0.88, 0.97))

truePrev(43, sum(43,815), SE = ~dunif(0.60, 0.95), SP = ~dunif(0.88, 0.97))

round(svytable(~igg_zikv_total+sex, design = svy), digits=0)

round(prop.table(svytable(~igg_zikv_total+sex, design = svy), margin = 2), digits = 3)

truePrev(633, sum(633,579), SE = ~dunif(0.75, 1.00), SP = ~dunif(0.60, 0.80))

truePrev(439, sum(439,419), SE = ~dunif(0.75, 1.00), SP = ~dunif(0.60, 0.80))

# AGE GROUPS

round(svytable(~igg_zikv+idgrupo, design = svy), digits=0)

round(prop.table(svytable(~igg_zikv+idgrupo, design = svy), margin = 2), digits = 3)

truePrev(64, sum(64,200), SE = ~dunif(0.75, 1.00), SP = ~dunif(0.60, 0.80), prior=c(6,4))

truePrev(162, sum(162,195), SE = ~dunif(0.75, 1.00), SP = ~dunif(0.60, 0.80), prior=c(6,4))

truePrev(160, sum(160,162), SE = ~dunif(0.75, 1.00), SP = ~dunif(0.60, 0.80), prior=c(6,4))

truePrev(206, sum(206,169), SE = ~dunif(0.75, 1.00), SP = ~dunif(0.60, 0.80), prior=c(6,4))

truePrev(235, sum(235,153), SE = ~dunif(0.75, 1.00), SP = ~dunif(0.60, 0.80), prior=c(6,4))

truePrev(217, sum(217,147), SE = ~dunif(0.75, 1.00), SP = ~dunif(0.60, 0.80), prior=c(6,4))

round(svytable(~igg3_zikv+idgrupo, design = svy), digits=0)

round(prop.table(svytable(~igg3_zikv+idgrupo, design = svy), margin = 2), digits = 3)

truePrev(11, sum(11,253), SE = ~dunif(0.60, 0.95), SP = ~dunif(0.88, 0.97), prior=c(6,4))

truePrev(14, sum(14,343), SE = ~dunif(0.60, 0.95), SP = ~dunif(0.88, 0.97), prior=c(6,4))

truePrev(13, sum(13,310), SE = ~dunif(0.60, 0.95), SP = ~dunif(0.88, 0.97), prior=c(6,4))

truePrev(26, sum(26,349), SE = ~dunif(0.60, 0.95), SP = ~dunif(0.88, 0.97), prior=c(6,4))

truePrev(19, sum(19,369), SE = ~dunif(0.60, 0.95), SP = ~dunif(0.88, 0.97), prior=c(6,4))

truePrev(21, sum(21,343), SE = ~dunif(0.60, 0.95), SP = ~dunif(0.88, 0.97), prior=c(6,4))

round(svytable(~igg_zikv_total+idgrupo, design = svy), digits=0)

round(prop.table(svytable(~igg_zikv_total+idgrupo, design = svy), margin = 2), digits = 3)

truePrev( 69, sum(69,195), SE = ~dunif(0.75, 1.00), SP = ~dunif(0.60, 0.80), prior=c(6,4))

truePrev(168, sum(168,190), SE = ~dunif(0.75, 1.00), SP = ~dunif(0.60, 0.80), prior=c(6,4))

truePrev(162, sum(162,161), SE = ~dunif(0.75, 1.00), SP = ~dunif(0.60, 0.80), prior=c(6,4))

truePrev(213, sum(213,162), SE = ~dunif(0.75, 1.00), SP = ~dunif(0.60, 0.80), prior=c(6,4))

truePrev(240, sum(240,148), SE = ~dunif(0.75, 1.00), SP = ~dunif(0.60, 0.80), prior=c(6,4))

truePrev(221, sum(221,143), SE = ~dunif(0.75, 1.00), SP = ~dunif(0.60, 0.80), prior=c(6,4))

###################

### Chikungunya ###

###################

#########

## IGG ##

#########

### Total

round(svytable(~igg_chikv, design = svy), digits=0)

svymean(~igg_chikv, svy, na.rm=TRUE)

confint(svymean(~igg_chikv, svy, na.rm=TRUE))

###Cluster

round(svytable(~igg_chikv+cluster, design = svy), digits=0)

round(prop.table(svytable(~igg_chikv+cluster, design = svy), margin = 2), digits = 3)

round(svyby(formula = ~igg_chikv, by = ~cluster, design = svy, FUN = svymean, na.rm=TRUE, vartype=c("se","ci")), digits = 3)

### Sex

round(svytable(~igg_chikv+sex, design = svy), digits=0)

round(prop.table(svytable(~igg_chikv+sex, design = svy), margin = 2), digits = 3)

round(svyby(formula = ~igg_chikv, by = ~sex, design = svy, FUN = svymean, na.rm=TRUE, vartype=c("se","ci")), digits = 3)

### AGE GROUPS

round(svytable(~igg_chikv+idgrupo, design = svy), digits=0)

round(prop.table(svytable(~igg_chikv+idgrupo, design = svy), margin = 2), digits = 3)

round(svyby(formula = ~igg_chikv, by = ~idgrupo, design = svy, FUN = svymean, na.rm=TRUE, vartype=c("se","ci")), digits = 3)

#########

## IGM ##

#########

### Total

round(svytable(~igm_chikv, design = svy), digits=0)

svymean(~igm_chikv, svy, na.rm=TRUE)

confint(svymean(~igm_chikv, svy, na.rm=TRUE))

### Cluster

round(svytable(~igm_chikv+cluster, design = svy), digits=0)

round(prop.table(svytable(~igm_chikv+cluster, design = svy), margin = 2), digits = 3)

round(svyby(formula = ~igm_chikv, by = ~cluster, design = svy, FUN = svymean, na.rm=TRUE, vartype=c("se","ci")), digits = 3)

### Sex

round(svytable(~igm_chikv+sex, design = svy), digits=0)

round(prop.table(svytable(~igm_chikv+sex, design = svy), margin = 2), digits = 3)

round(svyby(formula = ~igm_chikv, by = ~sex, design = svy, FUN = svymean, na.rm=TRUE, vartype=c("se","ci")), digits = 3)

### AGE GROUPS

round(svytable(~igm_chikv+idgrupo, design = svy), digits=0)

round(prop.table(svytable(~igm_chikv+idgrupo, design = svy), margin = 2), digits = 3)

round(svyby(formula = ~igm_chikv, by = ~idgrupo, design = svy, FUN = svymean, na.rm=TRUE, vartype=c("se","ci")), digits = 3)

#################

## CHIKV_TOTAL ##

#################

### Total

round(svytable(~chikv_total, design = svy), digits=0)

svymean(~chikv_total, svy, na.rm=TRUE)

confint(svymean(~chikv_total, svy, na.rm=TRUE))

### Cluster

round(svytable(~chikv_total+cluster, design = svy), digits=0)

round(prop.table(svytable(~chikv_total+cluster, design = svy), margin = 2), digits = 3)

round(svyby(formula = ~chikv_total, by = ~cluster, design = svy, FUN = svymean, na.rm=TRUE, vartype=c("se","ci")), digits = 3)

### Sex

round(svytable(~chikv_total+sex, design = svy), digits=0)

round(prop.table(svytable(~chikv_total+sex, design = svy), margin = 2), digits = 3)

round(svyby(formula = ~chikv_total, by = ~sex, design = svy, FUN = svymean, na.rm=TRUE, vartype=c("se","ci")), digits = 3)

### AGE GROUPS

round(svytable(~chikv_total+idgrupo, design = svy), digits=0)

round(prop.table(svytable(~chikv_total+idgrupo, design = svy), margin = 2), digits = 3)

round(svyby(formula = ~chikv_total, by = ~idgrupo, design = svy, FUN = svymean, na.rm=TRUE, vartype=c("se","ci")), digits = 3)

##############

# TABLE 2 #

##############

##############

## IGG DENV ##

##############

### SEX

# COMPARISONS IN EACH CLUSTER #

round(svytable(~igg_denv+sex, design = svy.cluster1), digits=0)

svychisq(~igg_denv+sex, svy.cluster1)

round(svyby(formula = ~igg_denv, by = ~sex, design = svy.cluster1, FUN = svymean, na.rm=TRUE, vartype=c("se","ci")), digits = 3)

round(svytable(~igg_denv+sex, design = svy.cluster2), digits=0)

svychisq(~igg_denv+sex, svy.cluster2)

round(svyby(formula = ~igg_denv, by = ~sex, design = svy.cluster2, FUN = svymean, na.rm=TRUE, vartype=c("se","ci")), digits = 3)

round(svytable(~igg_denv+sex, design = svy.cluster3), digits=0)

svychisq(~igg_denv+sex, svy.cluster3)

round(svyby(formula = ~igg_denv, by = ~sex, design = svy.cluster3, FUN = svymean, na.rm=TRUE, vartype=c("se","ci")), digits = 3)

# COMPARISONS BETWEEN CLUSTERS #

round(svytable(~igg_denv+cluster, design = svy.sexoF), digits=0)

svychisq(~igg_denv+cluster, svy.sexoF)

round(svyby(formula = ~igg_denv, by = ~cluster, design = svy.sexoF, FUN = svymean, na.rm=TRUE, vartype=c("se","ci")), digits = 3)

round(svytable(~igg_denv+cluster, design = svy.sexoM), digits=0)

svychisq(~igg_denv+cluster, svy.sexoM)

round(svyby(formula = ~igg_denv, by = ~cluster, design = svy.sexoM, FUN = svymean, na.rm=TRUE, vartype=c("se","ci")), digits = 3)

### IDGRUPO

# COMPARISONS IN EACH CLUSTER #

round(svytable(~igg_denv+idgrupo, design = svy.cluster1), digits=0)

svychisq(~igg_denv+idgrupo, svy.cluster1)

round(svyby(formula = ~igg_denv, by = ~idgrupo, design = svy.cluster1, FUN = svymean, na.rm=TRUE, vartype=c("se","ci")), digits = 3)

round(svytable(~igg_denv+idgrupo, design = svy.cluster2), digits=0)

svychisq(~igg_denv+idgrupo, svy.cluster2)

round(svyby(formula = ~igg_denv, by = ~idgrupo, design = svy.cluster2, FUN = svymean, na.rm=TRUE, vartype=c("se","ci")), digits = 3)

round(svytable(~igg_denv+idgrupo, design = svy.cluster3), digits=0)

svychisq(~igg_denv+idgrupo, svy.cluster3)

round(svyby(formula = ~igg_denv, by = ~idgrupo, design = svy.cluster3, FUN = svymean, na.rm=TRUE, vartype=c("se","ci")), digits = 3)

# COMPARISONS BETWEEN CLUSTERS #

round(svytable(~igg_denv+cluster, design = svy.idgrupo1), digits=0)

svychisq(~igg_denv+cluster, svy.idgrupo1)

round(svyby(formula = ~igg_denv, by = ~cluster, design = svy.idgrupo1, FUN = svymean, na.rm=TRUE, vartype=c("se","ci")), digits = 3)

round(svytable(~igg_denv+cluster, design = svy.idgrupo2), digits=0)

svychisq(~igg_denv+cluster, svy.idgrupo2)

round(svyby(formula = ~igg_denv, by = ~cluster, design = svy.idgrupo2, FUN = svymean, na.rm=TRUE, vartype=c("se","ci")), digits = 3)

round(svytable(~igg_denv+cluster, design = svy.idgrupo3), digits=0)

svychisq(~igg_denv+cluster, svy.idgrupo3)

round(svyby(formula = ~igg_denv, by = ~cluster, design = svy.idgrupo3, FUN = svymean, na.rm=TRUE, vartype=c("se","ci")), digits = 3)

round(svytable(~igg_denv+cluster, design = svy.idgrupo4), digits=0)

svychisq(~igg_denv+cluster, svy.idgrupo4)

round(svyby(formula = ~igg_denv, by = ~cluster, design = svy.idgrupo4, FUN = svymean, na.rm=TRUE, vartype=c("se","ci")), digits = 3)

round(svytable(~igg_denv+cluster, design = svy.idgrupo5), digits=0)

svychisq(~igg_denv+cluster, svy.idgrupo5)

round(svyby(formula = ~igg_denv, by = ~cluster, design = svy.idgrupo5, FUN = svymean, na.rm=TRUE, vartype=c("se","ci")), digits = 3)

round(svytable(~igg_denv+cluster, design = svy.idgrupo6), digits=0)

svychisq(~igg_denv+cluster, svy.idgrupo6)

round(svyby(formula = ~igg_denv, by = ~cluster, design = svy.idgrupo6, FUN = svymean, na.rm=TRUE, vartype=c("se","ci")), digits = 3)

### IDGRUPO3

# COMPARISONS IN EACH CLUSTER #

round(svytable(~igg_denv+idgrupo3, design = svy.cluster1), digits=0)

svychisq(~igg_denv+idgrupo3, svy.cluster1)

round(svyby(formula = ~igg_denv, by = ~idgrupo3, design = svy.cluster1, FUN = svymean, na.rm=TRUE, vartype=c("se","ci")), digits = 3)

round(svytable(~igg_denv+idgrupo3, design = svy.cluster2), digits=0)

svychisq(~igg_denv+idgrupo3, svy.cluster2)

round(svyby(formula = ~igg_denv, by = ~idgrupo3, design = svy.cluster2, FUN = svymean, na.rm=TRUE, vartype=c("se","ci")), digits = 3)

round(svytable(~igg_denv+idgrupo3, design = svy.cluster3), digits=0)

svychisq(~igg_denv+idgrupo3, svy.cluster3)

round(svyby(formula = ~igg_denv, by = ~idgrupo3, design = svy.cluster3, FUN = svymean, na.rm=TRUE, vartype=c("se","ci")), digits = 3)

# COMPARISONS BETWEEN CLUSTERS #

round(svytable(~igg_denv+cluster, design = svy.idgrupo3.1), digits=0)

svychisq(~igg_denv+cluster, svy.idgrupo3.1)

round(svyby(formula = ~igg_denv, by = ~cluster, design = svy.idgrupo3.1, FUN = svymean, na.rm=TRUE, vartype=c("se","ci")), digits = 3)

round(svytable(~igg_denv+cluster, design = svy.idgrupo3.2), digits=0)

svychisq(~igg_denv+cluster, svy.idgrupo3.2)

round(svyby(formula = ~igg_denv, by = ~cluster, design = svy.idgrupo3.2, FUN = svymean, na.rm=TRUE, vartype=c("se","ci")), digits = 3)

round(svytable(~igg_denv+cluster, design = svy.idgrupo3.3), digits=0)

svychisq(~igg_denv+cluster, svy.idgrupo3.3)

round(svyby(formula = ~igg_denv, by = ~cluster, design = svy.idgrupo3.3, FUN = svymean, na.rm=TRUE, vartype=c("se","ci")), digits = 3)

####################

## IGG_ZIKV_TOTAL ##

####################

### SEX

round(svytable(~igg_zikv_total+sex+cluster, design = svy), digits=0)

truePrev(84, sum(84,137), SE = ~dunif(0.75, 1.00), SP = ~dunif(0.60, 0.80), prior=c(1,1))

truePrev(73, sum(73,120), SE = ~dunif(0.75, 1.00), SP = ~dunif(0.60, 0.80), prior=c(1,1))

truePrev(240, sum(240,194), SE = ~dunif(0.75, 1.00), SP = ~dunif(0.60, 0.80), prior=c(1,1))

truePrev(160, sum(160,134), SE = ~dunif(0.75, 1.00), SP = ~dunif(0.60, 0.80), prior=c(1,1))

truePrev(309, sum(309,248), SE = ~dunif(0.75, 1.00), SP = ~dunif(0.60, 0.80), prior=c(1,1))

truePrev(206, sum(206,166), SE = ~dunif(0.75, 1.00), SP = ~dunif(0.60, 0.80), prior=c(1,1))

# COMPARISONS IN EACH CLUSTER #

round(svytable(~igg_zikv_total+sex+cluster, design = svy), digits=0)

temp1<-truePrev(84, sum(84,137), SE = ~dunif(0.75, 1.00), SP = ~dunif(0.60, 0.80), prior=c(1,1))

sexF_clusterH<-as.matrix(temp1)

temp2<-truePrev(73, sum(73,120), SE = ~dunif(0.75, 1.00), SP = ~dunif(0.60, 0.80), prior=c(1,1))

sexM_clusterH<-as.matrix(temp2)

t.test(sexF_clusterH[,1], sexM_clusterH[,1])

temp3<-truePrev(240, sum(240,194), SE = ~dunif(0.75, 1.00), SP = ~dunif(0.60, 0.80), prior=c(1,1))

sexF_clusterI<-as.matrix(temp3)

temp4<-truePrev(160, sum(160,134), SE = ~dunif(0.75, 1.00), SP = ~dunif(0.60, 0.80), prior=c(1,1))

sexM_clusterI<-as.matrix(temp4)

t.test(sexF_clusterI[,1], sexM_clusterI[,1])

temp5<-truePrev(309, sum(309,248), SE = ~dunif(0.75, 1.00), SP = ~dunif(0.60, 0.80), prior=c(1,1))

sexF_clusterL<-as.matrix(temp5)

temp6<-truePrev(206, sum(206,166), SE = ~dunif(0.75, 1.00), SP = ~dunif(0.60, 0.80), prior=c(1,1))

sexM_clusterL<-as.matrix(temp6)

t.test(sexF_clusterL[,1], sexM_clusterL[,1])

# COMPARISONS BETWEEN CLUSTERS #

round(svytable(~igg_zikv_total+sex+cluster, design = svy), digits=0)

temp34<-truePrev(84, sum(84,137), SE = ~dunif(0.75, 1.00), SP = ~dunif(0.60, 0.80), prior=c(1,1))

sexF_clusterH<-data.frame(id=1, y=as.matrix(temp34))

temp35<-truePrev(240, sum(240,194), SE = ~dunif(0.75, 1.00), SP = ~dunif(0.60, 0.80), prior=c(1,1))

sexF_clusterI<-data.frame(id=2, y=as.matrix(temp35))

temp36<-truePrev(309, sum(309,248), SE = ~dunif(0.75, 1.00), SP = ~dunif(0.60, 0.80), prior=c(1,1))

sexF_clusterL<-data.frame(id=3, y=as.matrix(temp36))

sexF<-rbind(sexF_clusterH,sexF_clusterI,sexF_clusterL)

names(sexF)

summary(aov(y.TP~id, data=sexF))

temp37<-truePrev(73, sum(73,120), SE = ~dunif(0.75, 1.00), SP = ~dunif(0.60, 0.80), prior=c(1,1))

sexM_clusterH<-data.frame(id=1, y=as.matrix(temp37))

temp38<-truePrev(160, sum(160,134), SE = ~dunif(0.75, 1.00), SP = ~dunif(0.60, 0.80), prior=c(1,1))

sexM_clusterI<-data.frame(id=2, y=as.matrix(temp38))

temp39<-truePrev(206, sum(206,166), SE = ~dunif(0.75, 1.00), SP = ~dunif(0.60, 0.80), prior=c(1,1))

sexM_clusterL<-data.frame(id=3, y=as.matrix(temp39))

sexM<-rbind(sexM_clusterH,sexM_clusterI,sexM_clusterL)

names(sexM)

summary(aov(y.TP~id, data=sexM))

### IDGRUPO

round(svytable(~igg_zikv_total+idgrupo+cluster, design = svy), digits=0)

truePrev(12, sum(12,28), SE = ~dunif(0.75, 1.00), SP = ~dunif(0.60, 0.80), prior=c(6,4))

truePrev(21, sum(21,45), SE = ~dunif(0.75, 1.00), SP = ~dunif(0.60, 0.80), prior=c(6,4))

truePrev(24, sum(24,46), SE = ~dunif(0.75, 1.00), SP = ~dunif(0.60, 0.80), prior=c(6,4))

truePrev(36, sum(36,50), SE = ~dunif(0.75, 1.00), SP = ~dunif(0.60, 0.80), prior=c(6,4))

truePrev(27, sum(27,34), SE = ~dunif(0.75, 1.00), SP = ~dunif(0.60, 0.80), prior=c(6,4))

truePrev(37, sum(37,54), SE = ~dunif(0.75, 1.00), SP = ~dunif(0.60, 0.80), prior=c(6,4))

truePrev(21, sum(21,68), SE = ~dunif(0.75, 1.00), SP = ~dunif(0.60, 0.80), prior=c(6,4))

truePrev(61, sum(61,67), SE = ~dunif(0.75, 1.00), SP = ~dunif(0.60, 0.80), prior=c(6,4))

truePrev(66, sum(66,49), SE = ~dunif(0.75, 1.00), SP = ~dunif(0.60, 0.80), prior=c(6,4))

truePrev(66, sum(66,40), SE = ~dunif(0.75, 1.00), SP = ~dunif(0.60, 0.80), prior=c(6,4))

truePrev(103,sum(103,54), SE = ~dunif(0.75, 1.00), SP = ~dunif(0.60, 0.80), prior=c(6,4))

truePrev(83, sum(83,49), SE = ~dunif(0.75, 1.00), SP = ~dunif(0.60, 0.80), prior=c(6,4))

truePrev(36, sum(36,98), SE = ~dunif(0.75, 1.00), SP = ~dunif(0.60, 0.80), prior=c(6,4))

truePrev(86, sum(86,78), SE = ~dunif(0.75, 1.00), SP = ~dunif(0.60, 0.80), prior=c(6,4))

truePrev(72, sum(72,66), SE = ~dunif(0.75, 1.00), SP = ~dunif(0.60, 0.80), prior=c(6,4))

truePrev(110, sum(110,72), SE = ~dunif(0.75, 1.00), SP = ~dunif(0.60, 0.80), prior=c(6,4))

truePrev(110, sum(110,60), SE = ~dunif(0.75, 1.00), SP = ~dunif(0.60, 0.80), prior=c(6,4))

truePrev(101, sum(101,40), SE = ~dunif(0.75, 1.00), SP = ~dunif(0.60, 0.80), prior=c(6,4))

# COMPARISONS IN EACH CLUSTER #

round(svytable(~igg_zikv_total+idgrupo+cluster, design = svy), digits=0)

temp7<-truePrev(12, sum(12,28), SE = ~dunif(0.75, 1.00), SP = ~dunif(0.60, 0.80), prior=c(6,4))

idgrupo1_clusterH<-data.frame(id=1, y=as.matrix(temp7))

temp8<-truePrev(21, sum(21,45), SE = ~dunif(0.75, 1.00), SP = ~dunif(0.60, 0.80), prior=c(6,4))

idgrupo2_clusterH<-data.frame(id=2, y=as.matrix(temp8))

temp9<-truePrev(24, sum(24,46), SE = ~dunif(0.75, 1.00), SP = ~dunif(0.60, 0.80), prior=c(6,4))

idgrupo3_clusterH<-data.frame(id=3, y=as.matrix(temp9))

temp10<-truePrev(36, sum(36,50), SE = ~dunif(0.75, 1.00), SP = ~dunif(0.60, 0.80), prior=c(6,4))

idgrupo4_clusterH<-data.frame(id=4, y=as.matrix(temp10))

temp11<-truePrev(27, sum(27,34), SE = ~dunif(0.75, 1.00), SP = ~dunif(0.60, 0.80), prior=c(6,4))

idgrupo5_clusterH<-data.frame(id=5, y=as.matrix(temp11))

temp12<-truePrev(37, sum(37,54), SE = ~dunif(0.75, 1.00), SP = ~dunif(0.60, 0.80), prior=c(6,4))

idgrupo6_clusterH<-data.frame(id=6, y=as.matrix(temp12))

idgrupo_clusterH<-rbind(idgrupo1_clusterH,idgrupo2_clusterH,idgrupo3_clusterH,idgrupo4_clusterH,idgrupo5_clusterH,idgrupo6_clusterH)

names(idgrupo_clusterH)

summary(aov(y.TP~id, data=idgrupo_clusterH))

temp13<-truePrev(21, sum(21,68), SE = ~dunif(0.75, 1.00), SP = ~dunif(0.60, 0.80), prior=c(6,4))

idgrupo1_clusterI<-data.frame(id=1, y=as.matrix(temp13))

temp14<-truePrev(61, sum(61,67), SE = ~dunif(0.75, 1.00), SP = ~dunif(0.60, 0.80), prior=c(6,4))

idgrupo2_clusterI<-data.frame(id=2, y=as.matrix(temp14))

temp15<-truePrev(66, sum(66,49), SE = ~dunif(0.75, 1.00), SP = ~dunif(0.60, 0.80), prior=c(6,4))

idgrupo3_clusterI<-data.frame(id=3, y=as.matrix(temp15))

temp16<-truePrev(66, sum(66,40), SE = ~dunif(0.75, 1.00), SP = ~dunif(0.60, 0.80), prior=c(6,4))

idgrupo4_clusterI<-data.frame(id=4, y=as.matrix(temp16))

temp17<-truePrev(103, sum(103,54), SE = ~dunif(0.75, 1.00), SP = ~dunif(0.60, 0.80), prior=c(6,4))

idgrupo5_clusterI<-data.frame(id=5, y=as.matrix(temp17))

temp18<-truePrev(83, sum(83,49), SE = ~dunif(0.75, 1.00), SP = ~dunif(0.60, 0.80), prior=c(6,4))

idgrupo6_clusterI<-data.frame(id=6, y=as.matrix(temp18))

idgrupo_clusterI<-rbind(idgrupo1_clusterI,idgrupo2_clusterI,idgrupo3_clusterI,idgrupo4_clusterI,idgrupo5_clusterI,idgrupo6_clusterI)

names(idgrupo_clusterI)

summary(aov(y.TP~id, data=idgrupo_clusterI))

temp19<-truePrev(36, sum(36,98), SE = ~dunif(0.75, 1.00), SP = ~dunif(0.60, 0.80), prior=c(6,4))

idgrupo1_clusterL<-data.frame(id=1, y=as.matrix(temp19))

temp20<-truePrev(86, sum(86,78), SE = ~dunif(0.75, 1.00), SP = ~dunif(0.60, 0.80), prior=c(6,4))

idgrupo2_clusterL<-data.frame(id=2, y=as.matrix(temp20))

temp21<-truePrev(72, sum(72,66), SE = ~dunif(0.75, 1.00), SP = ~dunif(0.60, 0.80), prior=c(6,4))

idgrupo3_clusterL<-data.frame(id=3, y=as.matrix(temp21))

temp22<-truePrev(110, sum(110,72), SE = ~dunif(0.75, 1.00), SP = ~dunif(0.60, 0.80), prior=c(6,4))

idgrupo4_clusterL<-data.frame(id=4, y=as.matrix(temp22))

temp23<-truePrev(110, sum(110,60), SE = ~dunif(0.75, 1.00), SP = ~dunif(0.60, 0.80), prior=c(6,4))

idgrupo5_clusterL<-data.frame(id=5, y=as.matrix(temp23))

temp24<-truePrev(101, sum(101,40), SE = ~dunif(0.75, 1.00), SP = ~dunif(0.60, 0.80), prior=c(6,4))

idgrupo6_clusterL<-data.frame(id=6, y=as.matrix(temp24))

idgrupo_clusterL<-rbind(idgrupo1_clusterL,idgrupo2_clusterL,idgrupo3_clusterL,idgrupo4_clusterL,idgrupo5_clusterL,idgrupo6_clusterL)

names(idgrupo_clusterL)

summary(aov(y.TP~id, data=idgrupo_clusterL))

# COMPARISONS BETWEEN CLUSTERS #

round(svytable(~igg_zikv_total+idgrupo+cluster, design = svy), digits=0)

temp40<-truePrev(12, sum(12,28), SE = ~dunif(0.75, 1.00), SP = ~dunif(0.60, 0.80), prior=c(6,4))

idgrupo1_clusterH<-data.frame(id=1, y=as.matrix(temp40))

temp41<-truePrev(21, sum(21,68), SE = ~dunif(0.75, 1.00), SP = ~dunif(0.60, 0.80), prior=c(6,4))

idgrupo1_clusterI<-data.frame(id=2, y=as.matrix(temp41))

temp42<-truePrev(36, sum(36,98), SE = ~dunif(0.75, 1.00), SP = ~dunif(0.60, 0.80), prior=c(6,4))

idgrupo1_clusterL<-data.frame(id=3, y=as.matrix(temp42))

idgrupo1<-rbind(idgrupo1_clusterH,idgrupo1_clusterI,idgrupo1_clusterL)

names(idgrupo1)

summary(aov(y.TP~id, data=idgrupo1))

temp43<-truePrev(21, sum(21,45), SE = ~dunif(0.75, 1.00), SP = ~dunif(0.60, 0.80), prior=c(6,4))

idgrupo2_clusterH<-data.frame(id=1, y=as.matrix(temp43))

temp44<-truePrev(61, sum(61,67), SE = ~dunif(0.75, 1.00), SP = ~dunif(0.60, 0.80), prior=c(6,4))

idgrupo2_clusterI<-data.frame(id=2, y=as.matrix(temp44))

temp45<-truePrev(86, sum(86,78), SE = ~dunif(0.75, 1.00), SP = ~dunif(0.60, 0.80), prior=c(6,4))

idgrupo2_clusterL<-data.frame(id=3, y=as.matrix(temp45))

idgrupo2<-rbind(idgrupo2_clusterH,idgrupo2_clusterI,idgrupo2_clusterL)

names(idgrupo2)

summary(aov(y.TP~id, data=idgrupo2))

temp46<-truePrev(24, sum(24,46), SE = ~dunif(0.75, 1.00), SP = ~dunif(0.60, 0.80), prior=c(6,4))

idgrupo3_clusterH<-data.frame(id=1, y=as.matrix(temp46))

temp47<-truePrev(66, sum(66,49), SE = ~dunif(0.75, 1.00), SP = ~dunif(0.60, 0.80), prior=c(6,4))

idgrupo3_clusterI<-data.frame(id=2, y=as.matrix(temp47))

temp48<-truePrev(72, sum(72,66), SE = ~dunif(0.75, 1.00), SP = ~dunif(0.60, 0.80), prior=c(6,4))

idgrupo3_clusterL<-data.frame(id=3, y=as.matrix(temp48))

idgrupo3<-rbind(idgrupo3_clusterH,idgrupo3_clusterI,idgrupo3_clusterL)

names(idgrupo3)

summary(aov(y.TP~id, data=idgrupo3))

temp49<-truePrev(36, sum(36,50), SE = ~dunif(0.75, 1.00), SP = ~dunif(0.60, 0.80), prior=c(6,4))

idgrupo4_clusterH<-data.frame(id=1, y=as.matrix(temp49))

temp50<-truePrev(66, sum(66,40), SE = ~dunif(0.75, 1.00), SP = ~dunif(0.60, 0.80), prior=c(6,4))

idgrupo4_clusterI<-data.frame(id=2, y=as.matrix(temp50))

temp51<-truePrev(110, sum(110,72), SE = ~dunif(0.75, 1.00), SP = ~dunif(0.60, 0.80), prior=c(6,4))

idgrupo4_clusterL<-data.frame(id=3, y=as.matrix(temp51))

idgrupo4<-rbind(idgrupo4_clusterH,idgrupo4_clusterI,idgrupo4_clusterL)

names(idgrupo4)

summary(aov(y.TP~id, data=idgrupo4))

temp52<-truePrev(27, sum(27,34), SE = ~dunif(0.75, 1.00), SP = ~dunif(0.60, 0.80), prior=c(6,4))

idgrupo5_clusterH<-data.frame(id=1, y=as.matrix(temp52))

temp53<-truePrev(103, sum(103,54), SE = ~dunif(0.75, 1.00), SP = ~dunif(0.60, 0.80), prior=c(6,4))

idgrupo5_clusterI<-data.frame(id=2, y=as.matrix(temp53))

temp54<-truePrev(110, sum(110,60), SE = ~dunif(0.75, 1.00), SP = ~dunif(0.60, 0.80), prior=c(6,4))

idgrupo5_clusterL<-data.frame(id=3, y=as.matrix(temp54))

idgrupo5<-rbind(idgrupo5_clusterH,idgrupo5_clusterI,idgrupo5_clusterL)

names(idgrupo5)

summary(aov(y.TP~id, data=idgrupo5))

temp55<-truePrev(37, sum(37,54), SE = ~dunif(0.75, 1.00), SP = ~dunif(0.60, 0.80), prior=c(6,4))

idgrupo6_clusterH<-data.frame(id=1, y=as.matrix(temp55))

temp56<-truePrev(83, sum(83,49), SE = ~dunif(0.75, 1.00), SP = ~dunif(0.60, 0.80), prior=c(6,4))

idgrupo6_clusterI<-data.frame(id=2, y=as.matrix(temp56))

temp57<-truePrev(101, sum(101,40), SE = ~dunif(0.75, 1.00), SP = ~dunif(0.60, 0.80), prior=c(6,4))

idgrupo6_clusterL<-data.frame(id=3, y=as.matrix(temp57))

idgrupo6<-rbind(idgrupo6_clusterH,idgrupo6_clusterI,idgrupo6_clusterL)

names(idgrupo6)

summary(aov(y.TP~id, data=idgrupo6))

### IDGRUPO3

round(svytable(~igg_zikv_total+idgrupo3+cluster, design = svy), digits=0)

truePrev(33, sum(33,73), SE = ~dunif(0.75, 1.00), SP = ~dunif(0.60, 0.80), prior=c(6,4))

truePrev(60, sum(60,96), SE = ~dunif(0.75, 1.00), SP = ~dunif(0.60, 0.80), prior=c(6,4))

truePrev(64, sum(64,88), SE = ~dunif(0.75, 1.00), SP = ~dunif(0.60, 0.80), prior=c(6,4))

truePrev(82, sum(82,135), SE = ~dunif(0.75, 1.00), SP = ~dunif(0.60, 0.80), prior=c(6,4))

truePrev(132, sum(132,89), SE = ~dunif(0.75, 1.00), SP = ~dunif(0.60, 0.80), prior=c(6,4))

truePrev(186, sum(186,104), SE = ~dunif(0.75, 1.00), SP = ~dunif(0.60, 0.80), prior=c(6,4))

truePrev(122, sum(122,176), SE = ~dunif(0.75, 1.00), SP = ~dunif(0.60, 0.80), prior=c(6,4))

truePrev(182, sum(182,138), SE = ~dunif(0.75, 1.00), SP = ~dunif(0.60, 0.80), prior=c(6,4))

truePrev(211, sum(211,100), SE = ~dunif(0.75, 1.00), SP = ~dunif(0.60, 0.80), prior=c(6,4))

# COMPARISONS IN EACH CLUSTER #

round(svytable(~igg_zikv_total+idgrupo3+cluster, design = svy), digits=0)

temp25<-truePrev(33, sum(33,73), SE = ~dunif(0.75, 1.00), SP = ~dunif(0.60, 0.80), prior=c(6,4))

idgrupo3.1_clusterH<-data.frame(id=1, y=as.matrix(temp25))

temp26<-truePrev(60, sum(60,96), SE = ~dunif(0.75, 1.00), SP = ~dunif(0.60, 0.80), prior=c(6,4))

idgrupo3.2_clusterH<-data.frame(id=2, y=as.matrix(temp26))

temp27<-truePrev(64, sum(64,88), SE = ~dunif(0.75, 1.00), SP = ~dunif(0.60, 0.80), prior=c(6,4))

idgrupo3.3_clusterH<-data.frame(id=3, y=as.matrix(temp27))

idgrupo3_clusterH<-rbind(idgrupo3.1_clusterH,idgrupo3.2_clusterH,idgrupo3.3_clusterH)

names(idgrupo3_clusterH)

summary(aov(y.TP~id, data=idgrupo3_clusterH))

temp28<-truePrev(82, sum(82,135), SE = ~dunif(0.75, 1.00), SP = ~dunif(0.60, 0.80), prior=c(6,4))

idgrupo3.1_clusterI<-data.frame(id=1, y=as.matrix(temp28))

temp29<-truePrev(132, sum(132,89), SE = ~dunif(0.75, 1.00), SP = ~dunif(0.60, 0.80), prior=c(6,4))

idgrupo3.2_clusterI<-data.frame(id=2, y=as.matrix(temp29))

temp30<-truePrev(186, sum(186,104), SE = ~dunif(0.75, 1.00), SP = ~dunif(0.60, 0.80), prior=c(6,4))

idgrupo3.3_clusterI<-data.frame(id=3, y=as.matrix(temp30))

idgrupo3_clusterI<-rbind(idgrupo3.1_clusterI,idgrupo3.2_clusterI,idgrupo3.3_clusterI)

names(idgrupo3_clusterI)

summary(aov(y.TP~id, data=idgrupo3_clusterI))

temp31<-truePrev(122, sum(122,176), SE = ~dunif(0.75, 1.00), SP = ~dunif(0.60, 0.80), prior=c(6,4))

idgrupo3.1_clusterL<-data.frame(id=1, y=as.matrix(temp31))

temp32<-truePrev(182, sum(182,138), SE = ~dunif(0.75, 1.00), SP = ~dunif(0.60, 0.80), prior=c(6,4))

idgrupo3.2_clusterL<-data.frame(id=2, y=as.matrix(temp32))

temp33<-truePrev(211, sum(211,100), SE = ~dunif(0.75, 1.00), SP = ~dunif(0.60, 0.80), prior=c(6,4))

idgrupo3.3_clusterL<-data.frame(id=3, y=as.matrix(temp33))

idgrupo3_clusterL<-rbind(idgrupo3.1_clusterL,idgrupo3.2_clusterL,idgrupo3.3_clusterL)

names(idgrupo3_clusterL)

summary(aov(y.TP~id, data=idgrupo3_clusterL))

# COMPARISONS BETWEEN CLUSTERS #

round(svytable(~igg_zikv_total+idgrupo3+cluster, design = svy), digits=0)

temp58<-truePrev(33, sum(33,73), SE = ~dunif(0.75, 1.00), SP = ~dunif(0.60, 0.80), prior=c(6,4))

idgrupo3.1_clusterH<-data.frame(id=1, y=as.matrix(temp58))

temp59<-truePrev(82, sum(82,135), SE = ~dunif(0.75, 1.00), SP = ~dunif(0.60, 0.80), prior=c(6,4))

idgrupo3.1_clusterI<-data.frame(id=2, y=as.matrix(temp59))

temp60<-truePrev(122, sum(122,176), SE = ~dunif(0.75, 1.00), SP = ~dunif(0.60, 0.80), prior=c(6,4))

idgrupo3.1_clusterL<-data.frame(id=3, y=as.matrix(temp60))

idgrupo3.1<-rbind(idgrupo3.1_clusterH,idgrupo3.1_clusterI,idgrupo3.1_clusterL)

names(idgrupo311)

summary(aov(y.TP~id, data=idgrupo3.1))

temp61<-truePrev(60, sum(60,96), SE = ~dunif(0.75, 1.00), SP = ~dunif(0.60, 0.80), prior=c(6,4))

idgrupo3.2_clusterH<-data.frame(id=1, y=as.matrix(temp61))

temp62<-truePrev(132, sum(132,89), SE = ~dunif(0.75, 1.00), SP = ~dunif(0.60, 0.80), prior=c(6,4))

idgrupo3.2_clusterI<-data.frame(id=2, y=as.matrix(temp62))

temp63<-truePrev(182, sum(182,138), SE = ~dunif(0.75, 1.00), SP = ~dunif(0.60, 0.80), prior=c(6,4))

idgrupo3.2_clusterL<-data.frame(id=3, y=as.matrix(temp63))

idgrupo3.2<-rbind(idgrupo3.2_clusterH,idgrupo3.2_clusterI,idgrupo3.2_clusterL)

names(idgrupo3.2)

summary(aov(y.TP~id, data=idgrupo3.2))

temp64<-truePrev(64, sum(64,88), SE = ~dunif(0.75, 1.00), SP = ~dunif(0.60, 0.80), prior=c(6,4))

idgrupo3.3_clusterH<-data.frame(id=1, y=as.matrix(temp64))

temp65<-truePrev(186, sum(186,104), SE = ~dunif(0.75, 1.00), SP = ~dunif(0.60, 0.80), prior=c(6,4))

idgrupo3.3_clusterI<-data.frame(id=2, y=as.matrix(temp65))

temp66<-truePrev(211, sum(211,100), SE = ~dunif(0.75, 1.00), SP = ~dunif(0.60, 0.80), prior=c(6,4))

idgrupo3.3_clusterL<-data.frame(id=3, y=as.matrix(temp66))

idgrupo3.3<-rbind(idgrupo3.3_clusterH,idgrupo3.3_clusterI,idgrupo3.3_clusterL)

names(idgrupo3.3)

summary(aov(y.TP~id, data=idgrupo3.3))

#################

## CHIKV_TOTAL ##

#################

### SEX

# COMPARISONS IN EACH CLUSTER #

round(svytable(~chikv_total+sex, design = svy.cluster1), digits=0)

svychisq(~chikv_total+sex, svy.cluster1)

round(svyby(formula = ~chikv_total, by = ~sex, design = svy.cluster1, FUN = svymean, na.rm=TRUE, vartype=c("se","ci")), digits = 3)

round(svytable(~chikv_total+sex, design = svy.cluster2), digits=0)

svychisq(~chikv_total+sex, svy.cluster2)

round(svyby(formula = ~chikv_total, by = ~sex, design = svy.cluster2, FUN = svymean, na.rm=TRUE, vartype=c("se","ci")), digits = 3)

round(svytable(~chikv_total+sex, design = svy.cluster3), digits=0)

svychisq(~chikv_total+sex, svy.cluster3)

round(svyby(formula = ~chikv_total, by = ~sex, design = svy.cluster3, FUN = svymean, na.rm=TRUE, vartype=c("se","ci")), digits = 3)

# COMPARISONS BETWEEN CLUSTERS #

round(svytable(~chikv_total+cluster, design = svy.sexoF), digits=0)

svychisq(~chikv_total+cluster, svy.sexoF)

round(svyby(formula = ~chikv_total, by = ~cluster, design = svy.sexoF, FUN = svymean, na.rm=TRUE, vartype=c("se","ci")), digits = 3)

round(svytable(~chikv_total+cluster, design = svy.sexoM), digits=0)

svychisq(~chikv_total+cluster, svy.sexoM)

round(svyby(formula = ~chikv_total, by = ~cluster, design = svy.sexoM, FUN = svymean, na.rm=TRUE, vartype=c("se","ci")), digits = 3)

### IDGRUPO

# COMPARISONS IN EACH CLUSTER #

round(svytable(~chikv_total+idgrupo, design = svy.cluster1), digits=0)

svychisq(~chikv_total+idgrupo, svy.cluster1)

round(svyby(formula = ~chikv_total, by = ~idgrupo, design = svy.cluster1, FUN = svymean, na.rm=TRUE, vartype=c("se","ci")), digits = 3)

round(svytable(~chikv_total+idgrupo, design = svy.cluster2), digits=0)

svychisq(~chikv_total+idgrupo, svy.cluster2)

round(svyby(formula = ~chikv_total, by = ~idgrupo, design = svy.cluster2, FUN = svymean, na.rm=TRUE, vartype=c("se","ci")), digits = 3)

round(svytable(~chikv_total+idgrupo, design = svy.cluster3), digits=0)

svychisq(~chikv_total+idgrupo, svy.cluster3)

round(svyby(formula = ~chikv_total, by = ~idgrupo, design = svy.cluster3, FUN = svymean, na.rm=TRUE, vartype=c("se","ci")), digits = 3)

# COMPARISONS BETWEEN CLUSTERS #

round(svytable(~chikv_total+cluster, design = svy.idgrupo1), digits=0)

svychisq(~chikv_total+cluster, svy.idgrupo1)

round(svyby(formula = ~chikv_total, by = ~cluster, design = svy.idgrupo1, FUN = svymean, na.rm=TRUE, vartype=c("se","ci")), digits = 3)

round(svytable(~chikv_total+cluster, design = svy.idgrupo2), digits=0)

svychisq(~chikv_total+cluster, svy.idgrupo2)

round(svyby(formula = ~chikv_total, by = ~cluster, design = svy.idgrupo2, FUN = svymean, na.rm=TRUE, vartype=c("se","ci")), digits = 3)

round(svytable(~chikv_total+cluster, design = svy.idgrupo3), digits=0)

svychisq(~chikv_total+cluster, svy.idgrupo3)

round(svyby(formula = ~chikv_total, by = ~cluster, design = svy.idgrupo3, FUN = svymean, na.rm=TRUE, vartype=c("se","ci")), digits = 3)

round(svytable(~chikv_total+cluster, design = svy.idgrupo4), digits=0)

svychisq(~chikv_total+cluster, svy.idgrupo4)

round(svyby(formula = ~chikv_total, by = ~cluster, design = svy.idgrupo4, FUN = svymean, na.rm=TRUE, vartype=c("se","ci")), digits = 3)

round(svytable(~chikv_total+cluster, design = svy.idgrupo5), digits=0)

svychisq(~chikv_total+cluster, svy.idgrupo5)

round(svyby(formula = ~chikv_total, by = ~cluster, design = svy.idgrupo5, FUN = svymean, na.rm=TRUE, vartype=c("se","ci")), digits = 3)

round(svytable(~chikv_total+cluster, design = svy.idgrupo6), digits=0)

svychisq(~chikv_total+cluster, svy.idgrupo6)

round(svyby(formula = ~chikv_total, by = ~cluster, design = svy.idgrupo6, FUN = svymean, na.rm=TRUE, vartype=c("se","ci")), digits = 3)

### IDGRUPO3

# COMPARISONS IN EACH CLUSTER #

round(svytable(~igg_denv+idgrupo3, design = svy.cluster1), digits=0)

svychisq(~igg_denv+idgrupo3, svy.cluster1)

round(svyby(formula = ~igg_denv, by = ~idgrupo3, design = svy.cluster1, FUN = svymean, na.rm=TRUE, vartype=c("se","ci")), digits = 3)

round(svytable(~igg_denv+idgrupo3, design = svy.cluster2), digits=0)

svychisq(~igg_denv+idgrupo3, svy.cluster2)

round(svyby(formula = ~igg_denv, by = ~idgrupo3, design = svy.cluster2, FUN = svymean, na.rm=TRUE, vartype=c("se","ci")), digits = 3)

round(svytable(~chikv_total+idgrupo3, design = svy.cluster3), digits=0)

svychisq(~chikv_total+idgrupo3, svy.cluster3)

round(svyby(formula = ~chikv_total, by = ~idgrupo3, design = svy.cluster3, FUN = svymean, na.rm=TRUE, vartype=c("se","ci")), digits = 3)

# COMPARISONS BETWEEN CLUSTERS #

round(svytable(~chikv_total+cluster, design = svy.idgrupo3.1), digits=0)

svychisq(~chikv_total+cluster, svy.idgrupo3.1)

round(svyby(formula = ~chikv_total, by = ~cluster, design = svy.idgrupo3.1, FUN = svymean, na.rm=TRUE, vartype=c("se","ci")), digits = 3)

round(svytable(~chikv_total+cluster, design = svy.idgrupo3.2), digits=0)

svychisq(~chikv_total+cluster, svy.idgrupo3.2)

round(svyby(formula = ~chikv_total, by = ~cluster, design = svy.idgrupo3.2, FUN = svymean, na.rm=TRUE, vartype=c("se","ci")), digits = 3)

round(svytable(~chikv_total+cluster, design = svy.idgrupo3.3), digits=0)

svychisq(~chikv_total+cluster, svy.idgrupo3.3)

round(svyby(formula = ~chikv_total, by = ~cluster, design = svy.idgrupo3.3, FUN = svymean, na.rm=TRUE, vartype=c("se","ci")), digits = 3)

##################

#### FIGURE 3 ####

##################

################

# READING DATA #

################

setwd("C:/Users/cflun/downloads/Cynthia Braga/Artigo 1 - Prevalência/")

banco<-read.csv("dados_figura3.csv", sep=";", dec=",")

# CLUSTER HIGH

cluster1<-subset(banco, ï..cluster ==1)

# CLUSTER INTERMEDIATE

cluster2<-subset(banco, ï..cluster ==2)

# CLUSTER DEPRIVED

cluster3<-subset(banco, ï..cluster ==3)

# CLUSTER OVERALL

overall<-subset(banco, ï..cluster ==4)

plot_mod_high = ggplot(aes(x=factor(var),y=prev), data=cluster1) +

geom_point(stat = "identity", size = 2) +

geom_errorbar(aes(ymin = lower, ymax = upper), width = 0.2) +

labs(title = "High", x= ' ', y = 'Seroprevalence (%)') +

ylim(0,100) +

theme(text = element_text(size=20)) +

theme_few() +

theme(axis.title = element_text(size = 10), axis.text = element_text(size = 10), strip.text = element_text(size = 16) ) +

theme(plot.title = element_text(hjust = 0.5))

plot_mod_high

plot_mod_intermediate = ggplot(aes(x=factor(var),y=prev), data=cluster2) +

geom_point(stat = "identity", size = 2) +

geom_errorbar(aes(ymin = lower, ymax = upper), width = 0.2) +

labs(title = "Intermediate", x= ' ', y = 'Seroprevalence (%)') +

ylim(0,100) +

theme(text = element_text(size=20)) +

theme_few() +

theme(axis.title = element_text(size = 10), axis.text = element_text(size = 10), strip.text = element_text(size = 16) ) +

theme(plot.title = element_text(hjust = 0.5))

plot_mod_intermediate

plot_mod_deprived = ggplot(aes(x=factor(var),y=prev), data=cluster3) +

geom_point(stat = "identity", size = 2) +

geom_errorbar(aes(ymin = lower, ymax = upper), width = 0.2) +

labs(title = "Deprived", x= ' ', y = 'Seroprevalence (%)') +

ylim(0,100) +

theme(text = element_text(size=20)) +

theme_few() +

theme(axis.title = element_text(size = 10), axis.text = element_text(size = 10), strip.text = element_text(size = 16) ) +

theme(plot.title = element_text(hjust = 0.5))

plot_mod_deprived

plot_mod_overall = ggplot(aes(x=factor(var),y=prev), data=overall) +

geom_point(stat = "identity", size = 2) +

geom_errorbar(aes(ymin = lower, ymax = upper), width = 0.2) +

labs(title = "Overall", x= ' ', y = 'Seroprevalence (%)') +

ylim(0,100) +

theme(text = element_text(size=20)) +

theme_few() +

theme(axis.title = element_text(size = 10), axis.text = element_text(size = 10), strip.text = element_text(size = 16) ) +

theme(plot.title = element_text(hjust = 0.5))

plot_mod_overall

plot_grid(plot_mod_high,plot_mod_intermediate,plot_mod_deprived,plot_mod_overall, nrow=2, ncol=2)

##################

#### FIGURE 4 ####

##################

##################################

# GLM ZIKV #

##################################

# Model Regression Cluster1

mod_glm1<-svyglm(igg_zikv_total~age, design=svy.cluster1,family=quasibinomial(link = "logit"))

summary(mod_glm1)

testdata1 = data.frame(age = seq(5, 65, length = 100))

fits1 = predict.glm(mod_glm1, newdata=testdata1, type='response', se=T)

fits1$fit<-fits1$fit*100

fits1$se.fit<-fits1$se.fit*100

predicts1 = data.frame(testdata1, fits1) %>% mutate(lower = fit - 1.96*se.fit, upper = fit + 1.96*se.fit) %>% mutate(cluster="1")

# Model Regression Cluster2

mod_glm2<-svyglm(igg_zikv_total~age, design=svy.cluster2,family=quasibinomial(link = "logit"))

summary(mod_glm2)

testdata2 = data.frame(age = seq(5, 65, length = 100))

fits2 = predict.glm(mod_glm2, newdata=testdata2, type='response', se=T)

fits2$fit<-fits2$fit*100

fits2$se.fit<-fits2$se.fit*100

predicts2 = data.frame(testdata2, fits2) %>% mutate(lower = fit - 1.96*se.fit, upper = fit + 1.96*se.fit) %>% mutate(cluster="2")

# Model Regression Cluster3

mod_glm3<-svyglm(igg_zikv_total~age, design=svy.cluster3,family=quasibinomial(link = "logit"))

summary(mod_glm3)

testdata3 = data.frame(age = seq(5, 65, length = 100))

fits3 = predict.glm(mod_glm3, newdata=testdata3, type='response', se=T)

fits3$fit<-fits3$fit*100

fits3$se.fit<-fits3$se.fit*100

predicts3 = data.frame(testdata3, fits3) %>% mutate(lower = fit - 1.96*se.fit, upper = fit + 1.96*se.fit) %>% mutate(cluster="3")

predicts_glm1<-rbind(predicts1,predicts2,predicts3)

predicts_glm1$cluster2 <- factor(predicts_glm1$cluster, labels = c("High", "Intermediate", "Deprived"))

# Graphical Display

plot_mod_glm1_response = ggplot(aes(x=age,y=fit), data=predicts_glm1) +

geom_ribbon(aes(ymin = lower, ymax=upper), fill='#DEEBF7') +

geom_line(color='#2171B5') +

labs(x = 'Age (years)', y = 'ZIKV Seroprev. (%)') +

scale_x_continuous(breaks = seq(5, 65, 5), expand = c(0, 5)) +

ylim(0,100) +

facet_wrap(~ cluster2, nrow=1, ncol=3) +

theme(text = element_text(size=20)) +

theme_few() +

theme(axis.title = element_text(size = 18), axis.text = element_text(size = 16), strip.text = element_text(size = 16) )

##################################

# GLM CHIKV #

##################################

# Model Regression Cluster1

mod_glm4<-svyglm(chikv_total~age, design=svy.cluster1,family=quasibinomial(link = "logit"))

summary(mod_glm4)

testdata4 = data.frame(age = seq(5, 65, length = 100))

fits4 = predict.glm(mod_glm4, newdata=testdata4, type='response', se=T)

fits4$fit<-fits4$fit*100

fits4$se.fit<-fits4$se.fit*100

predicts4 = data.frame(testdata4, fits4) %>% mutate(lower = fit - 1.96*se.fit, upper = fit + 1.96*se.fit) %>% mutate(cluster="High")

# Model Regression Cluster2

mod_glm5<-svyglm(chikv_total~age, design=svy.cluster2,family=quasibinomial(link = "logit"))

summary(mod_glm5)

testdata5 = data.frame(age = seq(5, 65, length = 100))

fits5 = predict.glm(mod_glm5, newdata=testdata5, type='response', se=T)

fits5$fit<-fits5$fit*100

fits5$se.fit<-fits5$se.fit*100

predicts5 = data.frame(testdata5, fits5) %>% mutate(lower = fit - 1.96*se.fit, upper = fit + 1.96*se.fit) %>% mutate(cluster="Intermediate")

# Model Regression Cluster3

mod_glm6<-svyglm(chikv_total~age, design=svy.cluster3,family=quasibinomial(link = "logit"))

summary(mod_glm6)

testdata6 = data.frame(age = seq(5, 65, length = 100))

fits6 = predict.glm(mod_glm6, newdata=testdata6, type='response', se=T)

fits6$fit<-fits6$fit*100

fits6$se.fit<-fits6$se.fit*100

predicts6 = data.frame(testdata6, fits6) %>% mutate(lower = fit - 1.96*se.fit, upper = fit + 1.96*se.fit) %>% mutate(cluster="Deprived")

predicts_glm2<-rbind(predicts4,predicts5,predicts6)

predicts_glm2$cluster2 <- factor(predicts_glm2$cluster, labels = c("High", "Intermediate", "Deprived"))

# Graphical Display

plot_mod_glm2_response = ggplot(aes(x=age,y=fit), data=predicts_glm2) +

geom_ribbon(aes(ymin = lower, ymax=upper), fill='#DEEBF7') +

geom_line(color='#2171B5') +

labs(x = 'Age (years)', y = 'CHIKV Seroprev. (%)') +

scale_x_continuous(breaks = seq(5, 65, 5), expand = c(0, 5)) +

ylim(0,100) +

facet_wrap(~ cluster2, nrow=1, ncol=3) +

theme(text = element_text(size=20)) +

theme_few() +

theme(axis.title = element_text(size = 18), axis.text = element_text(size = 16), strip.text = element_text(size = 16) )

################################

# GLM DENV #

################################

# Model Regression Cluster1

mod_glm7<-svyglm(igg_denv~age, design=svy.cluster1,family=quasibinomial(link = "logit"))

summary(mod_glm7)

testdata7 = data.frame(age = seq(5, 65, length = 100))

fits7 = predict.glm(mod_glm7, newdata=testdata7, type='response', se=T)

fits7$fit<-fits7$fit*100

fits7$se.fit<-fits7$se.fit*100

predicts7 = data.frame(testdata7, fits7) %>% mutate(lower = fit - 1.96*se.fit, upper = fit + 1.96*se.fit) %>% mutate(cluster="High")

predicts7$upper<-rec(predicts7$upper, rec="100:200=100; else=copy")

# Model Regression Cluster2

mod_glm8<-svyglm(igg_denv~age, design=svy.cluster2,family=quasibinomial(link = "logit"))

summary(mod_glm8)

testdata8 = data.frame(age = seq(5, 65, length = 100))

fits8 = predict.glm(mod_glm8, newdata=testdata8, type='response', se=T)

fits8$fit<-fits8$fit*100

fits8$se.fit<-fits8$se.fit*100

predicts8 = data.frame(testdata8, fits8) %>% mutate(lower = fit - 1.96*se.fit, upper = fit + 1.96*se.fit) %>% mutate(cluster="Intermediate")

predicts8$upper<-rec(predicts8$upper, rec="100:200=100; else=copy")

# Model Regression Cluster3

mod_glm9<-svyglm(igg_denv~age, design=svy.cluster3,family=quasibinomial(link = "logit"))

summary(mod_glm9)

testdata9 = data.frame(age = seq(5, 65, length = 100))

fits9 = predict.glm(mod_glm9, newdata=testdata9, type='response', se=T)

fits9$fit<-fits9$fit*100

fits9$se.fit<-fits9$se.fit*100

predicts9 = data.frame(testdata9, fits9) %>% mutate(lower = fit - 1.96*se.fit, upper = fit + 1.96*se.fit) %>% mutate(cluster="Deprived")

predicts9$upper<-rec(predicts9$upper, rec="100:200=100; else=copy")

predicts_glm3<-rbind(predicts7,predicts8,predicts9)

predicts_glm3$cluster2 <- factor(predicts_glm3$cluster, labels = c("High", "Intermediate", "Deprived"))

# Graphical Display

plot_mod_glm3_response = ggplot(aes(x=age,y=fit), data=predicts_glm3) +

geom_ribbon(aes(ymin = lower, ymax=upper), fill='#DEEBF7') +

geom_line(color='#2171B5') +

labs(x = 'Age (years)', y = 'DENV Seroprev. (%)') +

scale_x_continuous(breaks = seq(5, 65, 5), expand = c(0, 5)) +

ylim(0,100) +

facet_wrap(~ cluster2, nrow=1, ncol=3) +

theme_few() +

theme(axis.title = element_text(size = 18), axis.text = element_text(size = 16), strip.text = element_text(size = 16) )

plot_grid(plot_mod_glm3_response, plot_mod_glm1_response,plot_mod_glm2_response, nrow=3, ncol=1)

###################

#### FIGURE 4A ####

###################

#DENV

round(svytable(~igg_denv+idgrupo, design = svy.cluster1), digits=0)

a1<-round(svyby(formula = ~igg_denv, by = ~idgrupo, design = svy.cluster1, FUN = svymean, na.rm=TRUE, vartype=c("se","ci")), digits = 3)

a1 = data.frame(a1) %>% mutate(cluster="1")

round(svytable(~igg_denv+idgrupo, design = svy.cluster2), digits=0)

a2<-round(svyby(formula = ~igg_denv, by = ~idgrupo, design = svy.cluster2, FUN = svymean, na.rm=TRUE, vartype=c("se","ci")), digits = 3)

a2 = data.frame(a2) %>% mutate(cluster="2")

round(svytable(~igg_denv+idgrupo, design = svy.cluster3), digits=0)

a3<-round(svyby(formula = ~igg_denv, by = ~idgrupo, design = svy.cluster3, FUN = svymean, na.rm=TRUE, vartype=c("se","ci")), digits = 3)

a3 = data.frame(a3) %>% mutate(cluster="3")

a = rbind(a1,a2,a3)

a$cluster2 <- factor(a$cluster, labels = c("High", "Intermediate", "Deprived"))

dados_denv = a %>% select(idgrupo, igg_denv, ci_l, ci_u,cluster, cluster2)

dados_denv <- rename(dados_denv, prev=igg_denv, lower=ci_l, upper=ci_u)

dados_denv$prev<-dados_denv$prev*100

dados_denv$lower<-dados_denv$lower*100

dados_denv$upper<-dados_denv$upper*100

dados_denv$upper<-rec(dados_denv$upper, rec="100:200=100; else=copy")

# Graphical Display

plot_mod_denv = ggplot(aes(x=factor(idgrupo),y=prev), data=dados_denv) +

geom_point(stat = "identity", size = 2) +

geom_errorbar(aes(ymin = lower, ymax = upper), width = 0.2) +

labs(x = 'Age (years)', y = 'DENV Seroprev. (%)') +

scale_x_discrete(labels=c("05-14", "15-24", "25-34", "35-44", "45-54", "55-65")) +

ylim(0,100) +

facet_wrap(~ cluster2, nrow=1, ncol=3) +

theme(text = element_text(size=20)) +

theme_few() +

theme(axis.title = element_text(size = 16), axis.text = element_text(size = 16), strip.text = element_text(size = 16) )

#ZIKV

dados_zikv<-dados_denv

dados_zikv[1,2]=0.319

dados_zikv[1,3]=0.148

dados_zikv[1,4]=0.527

dados_zikv[2,2]=0.285

dados_zikv[2,3]=0.138

dados_zikv[2,4]=0.460

dados_zikv[3,2]=0.303

dados_zikv[3,3]=0.149

dados_zikv[3,4]=0.481

dados_zikv[4,2]=0.361

dados_zikv[4,3]=0.191

dados_zikv[4,4]=0.543

dados_zikv[5,2]=0.405

dados_zikv[5,3]=0.217

dados_zikv[5,4]=0.610

dados_zikv[6,2]=0.345

dados_zikv[6,3]=0.180

dados_zikv[6,4]=0.526

dados_zikv[7,2]=0.194

dados_zikv[7,3]=0.087

dados_zikv[7,4]=0.331

dados_zikv[8,2]=0.405

dados_zikv[8,3]=0.235

dados_zikv[8,4]=0.583

dados_zikv[9,2]=0.524

dados_zikv[9,3]=0.338

dados_zikv[9,4]=0.716

dados_zikv[10,2]=0.585

dados_zikv[10,3]=0.391

dados_zikv[10,4]=0.780

dados_zikv[11,2]=0.622

dados_zikv[11,3]=0.441

dados_zikv[11,4]=0.805

dados_zikv[12,2]=0.589

dados_zikv[12,3]=0.403

dados_zikv[12,4]=0.781

dados_zikv[13,2]=0.190

dados_zikv[13,3]=0.089

dados_zikv[13,4]=0.309

dados_zikv[14,2]=0.457

dados_zikv[14,3]=0.276

dados_zikv[14,4]=0.635

dados_zikv[15,2]=0.456

dados_zikv[15,3]=0.277

dados_zikv[15,4]=0.638

dados_zikv[16,2]=0.556

dados_zikv[16,3]=0.383

dados_zikv[16,4]=0.735

dados_zikv[17,2]=0.611

dados_zikv[17,3]=0.433

dados_zikv[17,4]=0.793

dados_zikv[18,2]=0.686

dados_zikv[18,3]=0.517

dados_zikv[18,4]=0.859

dados_zikv$prev<-dados_zikv$prev*100

dados_zikv$lower<-dados_zikv$lower*100

dados_zikv$upper<-dados_zikv$upper*100

dados_zikv$upper<-rec(dados_zikv$upper, rec="100:200=100; else=copy")

# Graphical Display

plot_mod_zikv = ggplot(aes(x=factor(idgrupo),y=prev), data=dados_zikv) +

geom_point(stat = "identity", size = 2) +

geom_errorbar(aes(ymin = lower, ymax = upper), width = 0.2) +

labs(x = 'Age (years)', y = 'ZIKV Seroprev. (%)') +

scale_x_discrete(labels=c("05-14", "15-24", "25-34", "35-44", "45-54", "55-65")) +

ylim(0,100) +

facet_wrap(~ cluster2, nrow=1, ncol=3) +

theme(text = element_text(size=20)) +

theme_few() +

theme(axis.title = element_text(size = 16), axis.text = element_text(size = 16), strip.text = element_text(size = 16) )

# CHIKV

round(svytable(~chikv_total+idgrupo, design = svy.cluster1), digits=0)

b1<-round(svyby(formula = ~chikv_total, by = ~idgrupo, design = svy.cluster1, FUN = svymean, na.rm=TRUE, vartype=c("se","ci")), digits = 3)

b1 = data.frame(b1) %>% mutate(cluster="1")

round(svytable(~chikv_total+idgrupo, design = svy.cluster2), digits=0)

b2<-round(svyby(formula = ~chikv_total, by = ~idgrupo, design = svy.cluster2, FUN = svymean, na.rm=TRUE, vartype=c("se","ci")), digits = 3)

b2 = data.frame(b2) %>% mutate(cluster="2")

round(svytable(~chikv_total+idgrupo, design = svy.cluster3), digits=0)

b3<-round(svyby(formula = ~chikv_total, by = ~idgrupo, design = svy.cluster3, FUN = svymean, na.rm=TRUE, vartype=c("se","ci")), digits = 3)

b3 = data.frame(b3) %>% mutate(cluster="3")

b = rbind(b1,b2,b3)

b$cluster2 <- factor(b$cluster, labels = c("High", "Intermediate", "Deprived"))

dados_chikv = b %>% select(idgrupo, chikv_total, ci_l, ci_u,cluster, cluster2)

dados_chikv <- rename(dados_chikv, prev=chikv_total, lower=ci_l, upper=ci_u)

dados_chikv$prev<-dados_chikv$prev*100

dados_chikv$lower<-dados_chikv$lower*100

dados_chikv$upper<-dados_chikv$upper*100

dados_chikv$upper<-rec(dados_chikv$upper, rec="100:200=100; else=copy")

plot_mod_chikv = ggplot(aes(x=factor(idgrupo),y=prev), data=dados_chikv) +

geom_point(stat = "identity", size = 2) +

geom_errorbar(aes(ymin = lower, ymax = upper), width = 0.2) +

labs(x = 'Age (years)', y = 'CHIKV Seroprev. (%)') +

scale_x_discrete(labels=c("05-14", "15-24", "25-34", "35-44", "45-54", "55-65")) +

ylim(0,100) +

facet_wrap(~ cluster2, nrow=1, ncol=3) +

theme(text = element_text(size=20)) +

theme_few() +

theme(axis.title = element_text(size = 16), axis.text = element_text(size = 16), strip.text = element_text(size = 16) )

plot_grid(plot_mod_denv, plot_mod_zikv,plot_mod_chikv, nrow=3, ncol=1)

###################

#### FIGURE 4B ####

###################

round(svytable(~igg_zikv_total+idgrupo, design = svy.cluster1), digits=0)

e1<-round(svyby(formula = ~igg_zikv_total, by = ~idgrupo, design = svy.cluster1, FUN = svymean, na.rm=TRUE, vartype=c("se","ci")), digits = 3)

e1 = data.frame(e1) %>% mutate(cluster="1")

round(svytable(~igg_zikv_total+idgrupo, design = svy.cluster2), digits=0)

e2<-round(svyby(formula = ~igg_zikv_total, by = ~idgrupo, design = svy.cluster2, FUN = svymean, na.rm=TRUE, vartype=c("se","ci")), digits = 3)

e2 = data.frame(e2) %>% mutate(cluster="2")

round(svytable(~igg_zikv_total+idgrupo, design = svy.cluster3), digits=0)

e3<-round(svyby(formula = ~igg_zikv_total, by = ~idgrupo, design = svy.cluster3, FUN = svymean, na.rm=TRUE, vartype=c("se","ci")), digits = 3)

e3 = data.frame(e3) %>% mutate(cluster="3")

e = rbind(e1,e2,e3)

e$cluster2 <- factor(e$cluster, labels = c("High", "Intermediate", "Deprived"))

dados_zikv1 = e %>% select(idgrupo, igg_zikv_total, ci_l, ci_u,cluster, cluster2)

dados_zikv1 <- rename(dados_zikv1, prev=igg_zikv_total, lower=ci_l, upper=ci_u)

dados_zikv1$prev<-dados_zikv1$prev*100

dados_zikv1$lower<-dados_zikv1$lower*100

dados_zikv1$upper<-dados_zikv1$upper*100

dados_zikv1$upper<-rec(dados_zikv1$upper, rec="100:200=100; else=copy")

# Graphical Display

plot_mod_zikv1 = ggplot(aes(x=factor(idgrupo),y=prev), data=dados_zikv1) +

geom_point(stat = "identity", size = 2) +

geom_errorbar(aes(ymin = lower, ymax = upper), width = 0.2) +

labs(x = 'Age (years)', y = 'ZIKV Seroprev. (%)') +

scale_x_discrete(labels=c("05-14", "15-24", "25-34", "35-44", "45-54", "55-65")) +

ylim(0,100) +

facet_wrap(~ cluster2, nrow=1, ncol=3) +

theme(text = element_text(size=20)) +

theme_few() +

theme(axis.title = element_text(size = 16), axis.text = element_text(size = 16), strip.text = element_text(size = 16) )

plot_grid(plot_mod_denv, plot_mod_zikv1,plot_mod_chikv, nrow=3, ncol=1)

##################

#### FIGURE 5 ####

##################

# CHIKV

round(svytable(~igg_chikv+idgrupo, design = svy.cluster1), digits=0)

c1<-round(svyby(formula = ~igg_chikv, by = ~idgrupo, design = svy.cluster1, FUN = svymean, na.rm=TRUE, vartype=c("se","ci")), digits = 3)

c1 = data.frame(c1) %>% mutate(cluster="1")

round(svytable(~igg_chikv+idgrupo, design = svy.cluster2), digits=0)

c2<-round(svyby(formula = ~igg_chikv, by = ~idgrupo, design = svy.cluster2, FUN = svymean, na.rm=TRUE, vartype=c("se","ci")), digits = 3)

c2 = data.frame(c2) %>% mutate(cluster="2")

round(svytable(~igg_chikv+idgrupo, design = svy.cluster3), digits=0)

c3<-round(svyby(formula = ~igg_chikv, by = ~idgrupo, design = svy.cluster3, FUN = svymean, na.rm=TRUE, vartype=c("se","ci")), digits = 3)

c3 = data.frame(c3) %>% mutate(cluster="3")

c = rbind(c1,c2,c3)

c$cluster2 <- factor(c$cluster, labels = c("High", "Intermediate", "Deprived"))

dados_chikv_igg = c %>% select(idgrupo, igg_chikv, ci_l, ci_u,cluster, cluster2)

dados_chikv_igg <- rename(dados_chikv_igg, prev=igg_chikv, lower=ci_l, upper=ci_u)

dados_chikv_igg$prev<-dados_chikv_igg$prev*100

dados_chikv_igg$lower<-dados_chikv_igg$lower*100

dados_chikv_igg$upper<-dados_chikv_igg$upper*100

dados_chikv_igg$upper<-rec(dados_chikv_igg$upper, rec="100:200=100; else=copy")

plot_mod_chikv_igg = ggplot(aes(x=factor(idgrupo),y=prev), data=dados_chikv_igg) +

geom_point(stat = "identity", size = 2) +

geom_errorbar(aes(ymin = lower, ymax = upper), width = 0.2) +

labs(x = 'Age (years)', y = 'CHIKV_IgG Seroprev. (%)') +

scale_x_discrete(labels=c("05-14", "15-24", "25-34", "35-44", "45-54", "55-65")) +

ylim(0,100) +

facet_wrap(~ cluster2, nrow=1, ncol=3) +

theme(text = element_text(size=20)) +

theme_few() +

theme(axis.title = element_text(size = 16), axis.text = element_text(size = 16), strip.text = element_text(size = 16) )

round(svytable(~igm_chikv+idgrupo, design = svy.cluster1), digits=0)

d1<-round(svyby(formula = ~igm_chikv, by = ~idgrupo, design = svy.cluster1, FUN = svymean, na.rm=TRUE, vartype=c("se","ci")), digits = 3)

d1 = data.frame(d1) %>% mutate(cluster="1")

round(svytable(~igm_chikv+idgrupo, design = svy.cluster2), digits=0)

d2<-round(svyby(formula = ~igm_chikv, by = ~idgrupo, design = svy.cluster2, FUN = svymean, na.rm=TRUE, vartype=c("se","ci")), digits = 3)

d2 = data.frame(d2) %>% mutate(cluster="2")

round(svytable(~igm_chikv++idgrupo, design = svy.cluster3), digits=0)

d3<-round(svyby(formula = ~igm_chikv, by = ~idgrupo, design = svy.cluster3, FUN = svymean, na.rm=TRUE, vartype=c("se","ci")), digits = 3)

d3 = data.frame(d3) %>% mutate(cluster="3")

d = rbind(d1,d2,d3)

d$cluster2 <- factor(d$cluster, labels = c("High", "Intermediate", "Deprived"))

dados_chikv_igm = d %>% select(idgrupo, igm_chikv, ci_l, ci_u,cluster, cluster2)

dados_chikv_igm <- rename(dados_chikv_igm, prev=igm_chikv, lower=ci_l, upper=ci_u)

dados_chikv_igm$prev<-dados_chikv_igm$prev*100

dados_chikv_igm$lower<-dados_chikv_igm$lower*100

dados_chikv_igm$upper<-dados_chikv_igm$upper*100

dados_chikv_igm$upper<-rec(dados_chikv_igm$upper, rec="100:200=100; else=copy")

plot_mod_chikv_igm = ggplot(aes(x=factor(idgrupo),y=prev), data=dados_chikv_igm) +

geom_point(stat = "identity", size = 2) +

geom_errorbar(aes(ymin = lower, ymax = upper), width = 0.2) +

labs(x = 'Age (years)', y = 'CHIKV_IgM Seroprev. (%)') +

scale_x_discrete(labels=c("05-14", "15-24", "25-34", "35-44", "45-54", "55-65")) +

ylim(0,100) +

facet_wrap(~ cluster2, nrow=1, ncol=3) +

theme(text = element_text(size=20)) +

theme_few() +

theme(axis.title = element_text(size = 16), axis.text = element_text(size = 16), strip.text = element_text(size = 16) )

plot_grid(plot_mod_chikv, plot_mod_chikv_igg,plot_mod_chikv_igm, nrow=3, ncol=1)

##################

#### FIGURE 6 ####

##################

### AGE GROUPS

round(svytable(~igg_zikv_total+idgrupo, design = svy.cluster1), digits=0)

round(prop.table(svytable(~igg_zikv_total+idgrupo, design = svy.cluster1), margin = 2), digits = 3)

round(svyby(formula = ~igg_zikv_total, by = ~idgrupo, design = svy.cluster1, FUN = svymean, na.rm=TRUE, vartype=c("se","ci")), digits = 3)

round(svytable(~igg_zikv_total+idgrupo, design = svy.cluster2), digits=0)

round(prop.table(svytable(~igg_zikv_total+idgrupo, design = svy.cluster2), margin = 2), digits = 3)

round(svyby(formula = ~igg_zikv_total, by = ~idgrupo, design = svy.cluster2, FUN = svymean, na.rm=TRUE, vartype=c("se","ci")), digits = 3)

round(svytable(~igg_zikv_total+idgrupo, design = svy.cluster3), digits=0)

round(prop.table(svytable(~igg_zikv_total+idgrupo, design = svy.cluster3), margin = 2), digits = 3)

round(svyby(formula = ~igg_zikv_total, by = ~idgrupo, design = svy.cluster3, FUN = svymean, na.rm=TRUE, vartype=c("se","ci")), digits = 3)

#ZIKV

dados_zikv_total<-dados_denv

dados_zikv_total[1,2]=0.298

dados_zikv_total[1,3]=0.141

dados_zikv_total[1,4]=0.455

dados_zikv_total[2,2]=0.316

dados_zikv_total[2,3]=0.191

dados_zikv_total[2,4]=0.441

dados_zikv_total[3,2]=0.346

dados_zikv_total[3,3]=0.200

dados_zikv_total[3,4]=0.492

dados_zikv_total[4,2]=0.420

dados_zikv_total[4,3]=0.284

dados_zikv_total[4,4]=0.556

dados_zikv_total[5,2]=0.443

dados_zikv_total[5,3]=0.316

dados_zikv_total[5,4]=0.569

dados_zikv_total[6,2]=0.406

dados_zikv_total[6,3]=0.296

dados_zikv_total[6,4]=0.516

dados_zikv_total[7,2]=0.240

dados_zikv_total[7,3]=0.147

dados_zikv_total[7,4]=0.333

dados_zikv_total[8,2]=0.476

dados_zikv_total[8,3]=0.387

dados_zikv_total[8,4]=0.564

dados_zikv_total[9,2]=0.574

dados_zikv_total[9,3]=0.480

dados_zikv_total[9,4]=0.667

dados_zikv_total[10,2]=0.622

dados_zikv_total[10,3]=0.516

dados_zikv_total[10,4]=0.728

dados_zikv_total[11,2]=0.653

dados_zikv_total[11,3]=0.577

dados_zikv_total[11,4]=0.730

dados_zikv_total[12,2]=0.628

dados_zikv_total[12,3]=0.526

dados_zikv_total[12,4]=0.730

dados_zikv_total[13,2]=0.268

dados_zikv_total[13,3]=0.173

dados_zikv_total[13,4]=0.363

dados_zikv_total[14,2]=0.526

dados_zikv_total[14,3]=0.420

dados_zikv_total[14,4]=0.631

dados_zikv_total[15,2]=0.522

dados_zikv_total[15,3]=0.415

dados_zikv_total[15,4]=0.628

dados_zikv_total[16,2]=0.605

dados_zikv_total[16,3]=0.532

dados_zikv_total[16,4]=0.678

dados_zikv_total[17,2]=0.648

dados_zikv_total[17,3]=0.556

dados_zikv_total[17,4]=0.740

dados_zikv_total[18,2]=0.718

dados_zikv_total[18,3]=0.633

dados_zikv_total[18,4]=0.803

dados_zikv_total$prev<-dados_zikv_total$prev*100

dados_zikv_total$lower<-dados_zikv_total$lower*100

dados_zikv_total$upper<-dados_zikv_total$upper*100

dados_zikv_total$upper<-rec(dados_zikv_total$upper, rec="100:200=100; else=copy")

# Graphical Display

plot_mod_zikv_total = ggplot(aes(x=factor(idgrupo),y=prev), data=dados_zikv_total) +

geom_point(stat = "identity", size = 2) +

geom_errorbar(aes(ymin = lower, ymax = upper), width = 0.2) +

labs(x = 'Age (years)', y = 'ZIKV Seroprev. (%)') +

scale_x_discrete(labels=c("05-14", "15-24", "25-34", "35-44", "45-54", "55-65")) +

ylim(0,100) +

facet_wrap(~ cluster2, nrow=1, ncol=3) +

theme(text = element_text(size=20)) +

theme_few() +

theme(axis.title = element_text(size = 16), axis.text = element_text(size = 16), strip.text = element_text(size = 16) )

round(svytable(~igg_zikv+idgrupo, design = svy.cluster1), digits=0)

round(prop.table(svytable(~igg_zikv+idgrupo, design = svy.cluster1), margin = 2), digits = 3)

round(svyby(formula = ~igg_zikv, by = ~idgrupo, design = svy.cluster1, FUN = svymean, na.rm=TRUE, vartype=c("se","ci")), digits = 3)

round(svytable(~igg_zikv+idgrupo, design = svy.cluster2), digits=0)

round(prop.table(svytable(~igg_zikv+idgrupo, design = svy.cluster2), margin = 2), digits = 3)

round(svyby(formula = ~igg_zikv, by = ~idgrupo, design = svy.cluster2, FUN = svymean, na.rm=TRUE, vartype=c("se","ci")), digits = 3)

round(svytable(~igg_zikv+idgrupo, design = svy.cluster3), digits=0)

round(prop.table(svytable(~igg_zikv+idgrupo, design = svy.cluster3), margin = 2), digits = 3)

round(svyby(formula = ~igg_zikv, by = ~idgrupo, design = svy.cluster3, FUN = svymean, na.rm=TRUE, vartype=c("se","ci")), digits = 3)

dados_zikv_igg<-dados_denv

dados_zikv_igg[1,2]=0.234

dados_zikv_igg[1,3]=0.114

dados_zikv_igg[1,4]=0.354

dados_zikv_igg[2,2]=0.303

dados_zikv_igg[2,3]=0.177

dados_zikv_igg[2,4]=0.428

dados_zikv_igg[3,2]=0.321

dados_zikv_igg[3,3]=0.176

dados_zikv_igg[3,4]=0.466

dados_zikv_igg[4,2]=0.390

dados_zikv_igg[4,3]=0.259

dados_zikv_igg[4,4]=0.521

dados_zikv_igg[5,2]=0.443

dados_zikv_igg[5,3]=0.316

dados_zikv_igg[5,4]=0.569

dados_zikv_igg[6,2]=0.396

dados_zikv_igg[6,3]=0.284

dados_zikv_igg[6,4]=0.508

dados_zikv_igg[7,2]=0.220

dados_zikv_igg[7,3]=0.120

dados_zikv_igg[7,4]=0.320

dados_zikv_igg[8,2]=0.448

dados_zikv_igg[8,3]=0.349

dados_zikv_igg[8,4]=0.546

dados_zikv_igg[9,2]=0.574

dados_zikv_igg[9,3]=0.480

dados_zikv_igg[9,4]=0.667

dados_zikv_igg[10,2]=0.580

dados_zikv_igg[10,3]=0.479

dados_zikv_igg[10,4]=0.681

dados_zikv_igg[11,2]=0.631

dados_zikv_igg[11,3]=0.561

dados_zikv_igg[11,4]=0.701

dados_zikv_igg[12,2]=0.622

dados_zikv_igg[12,3]=0.520

dados_zikv_igg[12,4]=0.723

dados_zikv_igg[13,2]=0.259

dados_zikv_igg[13,3]=0.161

dados_zikv_igg[13,4]=0.357

dados_zikv_igg[14,2]=0.518

dados_zikv_igg[14,3]=0.410

dados_zikv_igg[14,4]=0.626

dados_zikv_igg[15,2]=0.522

dados_zikv_igg[15,3]=0.415

dados_zikv_igg[15,4]=0.628

dados_zikv_igg[16,2]=0.605

dados_zikv_igg[16,3]=0.532

dados_zikv_igg[16,4]=0.678

dados_zikv_igg[17,2]=0.641

dados_zikv_igg[17,3]=0.546

dados_zikv_igg[17,4]=0.736

dados_zikv_igg[18,2]=0.701

dados_zikv_igg[18,3]=0.608

dados_zikv_igg[18,4]=0.793

dados_zikv_igg$prev<-dados_zikv_igg$prev*100

dados_zikv_igg$lower<-dados_zikv_igg$lower*100

dados_zikv_igg$upper<-dados_zikv_igg$upper*100

dados_zikv_igg$upper<-rec(dados_zikv_igg$upper, rec="100:200=100; else=copy")

# Graphical Display

plot_mod_zikv_igg = ggplot(aes(x=factor(idgrupo),y=prev), data=dados_zikv_igg) +

geom_point(stat = "identity", size = 2) +

geom_errorbar(aes(ymin = lower, ymax = upper), width = 0.2) +

labs(x = 'Age (years)', y = 'ZIKV_IgG Seroprev. (%)') +

scale_x_discrete(labels=c("05-14", "15-24", "25-34", "35-44", "45-54", "55-65")) +

ylim(0,100) +

facet_wrap(~ cluster2, nrow=1, ncol=3) +

theme(text = element_text(size=20)) +

theme_few() +

theme(axis.title = element_text(size = 16), axis.text = element_text(size = 16), strip.text = element_text(size = 16) )

round(svytable(~igg3_zikv+idgrupo, design = svy.cluster1), digits=0)

round(prop.table(svytable(~igg3_zikv+idgrupo, design = svy.cluster1), margin = 2), digits = 3)

round(svyby(formula = ~igg3_zikv, by = ~idgrupo, design = svy.cluster1, FUN = svymean, na.rm=TRUE, vartype=c("se","ci")), digits = 3)

round(svytable(~igg3_zikv+idgrupo, design = svy.cluster2), digits=0)

round(prop.table(svytable(~igg3_zikv+idgrupo, design = svy.cluster2), margin = 2), digits = 3)

round(svyby(formula = ~igg3_zikv, by = ~idgrupo, design = svy.cluster2, FUN = svymean, na.rm=TRUE, vartype=c("se","ci")), digits = 3)

round(svytable(~igg3_zikv+idgrupo, design = svy.cluster3), digits=0)

round(prop.table(svytable(~igg3_zikv+idgrupo, design = svy.cluster3), margin = 2), digits = 3)

round(svyby(formula = ~igg3_zikv, by = ~idgrupo, design = svy.cluster3, FUN = svymean, na.rm=TRUE, vartype=c("se","ci")), digits = 3)

dados_zikv_igm<-dados_denv

dados_zikv_igm[1,2]=0.106

dados_zikv_igm[1,3]=0.000

dados_zikv_igm[1,4]=0.219

dados_zikv_igm[2,2]=0.026

dados_zikv_igm[2,3]=0.000

dados_zikv_igm[2,4]=0.075

dados_zikv_igm[3,2]=0.049

dados_zikv_igm[3,3]=0.006

dados_zikv_igm[3,4]=0.093

dados_zikv_igm[4,2]=0.090

dados_zikv_igm[4,3]=0.028

dados_zikv_igm[4,4]=0.152

dados_zikv_igm[5,2]=0.071

dados_zikv_igm[5,3]=0.014

dados_zikv_igm[5,4]=0.129

dados_zikv_igm[6,2]=0.038

dados_zikv_igm[6,3]=0.004

dados_zikv_igm[6,4]=0.072

dados_zikv_igm[7,2]=0.040

dados_zikv_igm[7,3]=0.004

dados_zikv_igm[7,4]=0.076

dados_zikv_igm[8,2]=0.070

dados_zikv_igm[8,3]=0.028

dados_zikv_igm[8,4]=0.112

dados_zikv_igm[9,2]=0.031

dados_zikv_igm[9,3]=0.002

dados_zikv_igm[9,4]=0.060

dados_zikv_igm[10,2]=0.092

dados_zikv_igm[10,3]=0.040

dados_zikv_igm[10,4]=0.145

dados_zikv_igm[11,2]=0.040

dados_zikv_igm[11,3]=0.014

dados_zikv_igm[11,4]=0.066

dados_zikv_igm[12,2]=0.068

dados_zikv_igm[12,3]=0.020

dados_zikv_igm[12,4]=0.115

dados_zikv_igm[13,2]=0.027

dados_zikv_igm[13,3]=0.000

dados_zikv_igm[13,4]=0.055

dados_zikv_igm[14,2]=0.022

dados_zikv_igm[14,3]=0.000

dados_zikv_igm[14,4]=0.054

dados_zikv_igm[15,2]=0.043

dados_zikv_igm[15,3]=0.000

dados_zikv_igm[15,4]=0.086

dados_zikv_igm[16,2]=0.046

dados_zikv_igm[16,3]=0.008

dados_zikv_igm[16,4]=0.084

dados_zikv_igm[17,2]=0.049

dados_zikv_igm[17,3]=0.012

dados_zikv_igm[17,4]=0.086

dados_zikv_igm[18,2]=0.060

dados_zikv_igm[18,3]=0.011

dados_zikv_igm[18,4]=0.109

dados_zikv_igm$prev<-dados_zikv_igm$prev*100

dados_zikv_igm$lower<-dados_zikv_igm$lower*100

dados_zikv_igm$upper<-dados_zikv_igm$upper*100

dados_zikv_igm$upper<-rec(dados_zikv_igm$upper, rec="100:200=100; else=copy")

# Graphical Display

plot_mod_zikv_igm = ggplot(aes(x=factor(idgrupo),y=prev), data=dados_zikv_igm) +

geom_point(stat = "identity", size = 2) +

geom_errorbar(aes(ymin = lower, ymax = upper), width = 0.2) +

labs(x = 'Age (years)', y = 'ZIKV_IgM Seroprev. (%)') +

scale_x_discrete(labels=c("05-14", "15-24", "25-34", "35-44", "45-54", "55-65")) +

ylim(0,100) +

facet_wrap(~ cluster2, nrow=1, ncol=3) +

theme(text = element_text(size=20)) +

theme_few() +

theme(axis.title = element_text(size = 16), axis.text = element_text(size = 16), strip.text = element_text(size = 16) )

plot_grid(plot_mod_zikv_total, plot_mod_zikv_igg,plot_mod_zikv_igm, nrow=3, ncol=1)

###################

# FORCE INFECTION #

###################

# CARREGANDO OS PACOTES #

require(foreign)

library(prevalence)

require(survey)

require(prevalence)

library(MASS)

library(mgcv)

library(dplyr)

library(ggplot2)

library(ggnewscale)

library(ggplot2)

library(tidyverse)

library(gridExtra)

library(rmarkdown)

library(visibly)

library(ggplot2)

library(visreg)

require(ggthemes)

require(sjmisc)

require(cowplot)

################

# READING DATA #

################

setwd("C:/Users/cflun/downloads/Cynthia Braga/Fatores associados")

dados1<-read.csv("dados_smv.csv", sep=";", dec=",")

# CLUSTER HIGH

cluster1<-subset(dados1, cluster==1)

# CLUSTER INTERMEDIATE

cluster2<-subset(dados1, cluster==2)

# CLUSTER DEPRIVED

cluster3<-subset(dados1, cluster==3)

######################################

# EFEITO DO DESENHO E PLANO AMOSTRAL #

######################################

options(survey.lonely.psu = "certainty")

svy<-svydesign(id=~cod_setor+cod_dom, fpc=~fpc1+fpc2, strata=~cluster,weights=~peso, data=dados1, nest=TRUE)

svy.cluster1<-svydesign(id=~cod_setor+cod_dom, fpc=~fpc1+fpc2, weights=~peso, data=cluster1, nest=TRUE)

svy.cluster2<-svydesign(id=~cod_setor+cod_dom, fpc=~fpc1+fpc2, weights=~peso, data=cluster2, nest=TRUE)

svy.cluster3<-svydesign(id=~cod_setor+cod_dom, fpc=~fpc1+fpc2, weights=~peso, data=cluster3, nest=TRUE)

##################################

# GLM ZIKV #

##################################

# Model Regression Cluster1

#mod_glm1<-svyglm(igg_zikv_total~age, design=svy.cluster1,family=quasibinomial(link = "logit"))

#summary(mod_glm1)

mod_glm1<-svyglm(igg_zikv_total~log(age), design=svy.cluster1,family=quasibinomial(link = "cloglog"))

summary(mod_glm1)

testdata1 = data.frame(age = seq(5, 65, length = 100))

fits1 = predict.glm(mod_glm1, newdata=testdata1, type='response', se=T)

fits1$fit<-fits1$fit*100

fits1$se.fit<-fits1$se.fit*100

predicts1 = data.frame(testdata1, fits1) %>% mutate(lower = fit - 1.96*se.fit, upper = fit + 1.96*se.fit) %>% mutate(cluster="1")

# Model Regression Cluster2

#mod_glm2<-svyglm(igg_zikv_total~age, design=svy.cluster2,family=quasibinomial(link = "logit"))

#summary(mod_glm2)

mod_glm2<-svyglm(igg_zikv_total~log(age), design=svy.cluster2,family=quasibinomial(link = "cloglog"))

summary(mod_glm2)

testdata2 = data.frame(age = seq(5, 65, length = 100))

fits2 = predict.glm(mod_glm2, newdata=testdata2, type='response', se=T)

fits2$fit<-fits2$fit*100

fits2$se.fit<-fits2$se.fit*100

predicts2 = data.frame(testdata2, fits2) %>% mutate(lower = fit - 1.96*se.fit, upper = fit + 1.96*se.fit) %>% mutate(cluster="2")

# Model Regression Cluster3

#mod_glm3<-svyglm(igg_zikv_total~age, design=svy.cluster3,family=quasibinomial(link = "logit"))

#summary(mod_glm3)

mod_glm3<-svyglm(igg_zikv_total~log(age), design=svy.cluster3,family=quasibinomial(link = "cloglog"))

summary(mod_glm3)

testdata3 = data.frame(age = seq(5, 65, length = 100))

fits3 = predict.glm(mod_glm3, newdata=testdata3, type='response', se=T)

fits3$fit<-fits3$fit*100

fits3$se.fit<-fits3$se.fit*100

predicts3 = data.frame(testdata3, fits3) %>% mutate(lower = fit - 1.96*se.fit, upper = fit + 1.96*se.fit) %>% mutate(cluster="3")

predicts_glm1<-rbind(predicts1,predicts2,predicts3)

predicts_glm1$cluster2 <- factor(predicts_glm1$cluster, labels = c("High", "Intermediate", "Deprived"))

# Graphical Display

plot_mod_glm1_response = ggplot(aes(x=age,y=fit), data=predicts_glm1) +

geom_ribbon(aes(ymin = lower, ymax=upper), fill='#DEEBF7') +

geom_line(color='#2171B5') +

labs(x = 'Age (years)', y = 'ZIKV Seroprev. (%)') +

scale_x_continuous(breaks = seq(5, 65, 5), expand = c(0, 5)) +

ylim(0,100) +

facet_wrap(~ cluster2, nrow=1, ncol=3) +

theme(text = element_text(size=20)) +

theme_few() +

theme(axis.title = element_text(size = 18), axis.text = element_text(size = 16), strip.text = element_text(size = 16) )

##################################

# GLM CHIKV #

##################################

# Model Regression Cluster1

#mod_glm4<-svyglm(chikv_total~age, design=svy.cluster1,family=quasibinomial(link = "logit"))

#summary(mod_glm4)

mod_glm4<-svyglm(chikv_total~log(age), design=svy.cluster1,family=quasibinomial(link = "cloglog"))

summary(mod_glm4)

testdata4 = data.frame(age = seq(5, 65, length = 100))

fits4 = predict.glm(mod_glm4, newdata=testdata4, type='response', se=T)

fits4$fit<-fits4$fit*100

fits4$se.fit<-fits4$se.fit*100

predicts4 = data.frame(testdata4, fits4) %>% mutate(lower = fit - 1.96*se.fit, upper = fit + 1.96*se.fit) %>% mutate(cluster="High")

# Model Regression Cluster2

#mod_glm5<-svyglm(chikv_total~age, design=svy.cluster2,family=quasibinomial(link = "logit"))

#summary(mod_glm5)

mod_glm5<-svyglm(chikv_total~log(age), design=svy.cluster2,family=quasibinomial(link = "cloglog"))

summary(mod_glm5)

testdata5 = data.frame(age = seq(5, 65, length = 100))

fits5 = predict.glm(mod_glm5, newdata=testdata5, type='response', se=T)

fits5$fit<-fits5$fit*100

fits5$se.fit<-fits5$se.fit*100

predicts5 = data.frame(testdata5, fits5) %>% mutate(lower = fit - 1.96*se.fit, upper = fit + 1.96*se.fit) %>% mutate(cluster="Intermediate")

# Model Regression Cluster3

#mod_glm6<-svyglm(chikv_total~age, design=svy.cluster3,family=quasibinomial(link = "logit"))

#summary(mod_glm6)

mod_glm6<-svyglm(chikv_total~log(age), design=svy.cluster3,family=quasibinomial(link = "cloglog"))

summary(mod_glm6)

testdata6 = data.frame(age = seq(5, 65, length = 100))

fits6 = predict.glm(mod_glm6, newdata=testdata6, type='response', se=T)

fits6$fit<-fits6$fit*100

fits6$se.fit<-fits6$se.fit*100

predicts6 = data.frame(testdata6, fits6) %>% mutate(lower = fit - 1.96*se.fit, upper = fit + 1.96*se.fit) %>% mutate(cluster="Deprived")

predicts_glm2<-rbind(predicts4,predicts5,predicts6)

predicts_glm2$cluster2 <- factor(predicts_glm2$cluster, labels = c("High", "Intermediate", "Deprived"))

# Graphical Display

plot_mod_glm2_response = ggplot(aes(x=age,y=fit), data=predicts_glm2) +

geom_ribbon(aes(ymin = lower, ymax=upper), fill='#DEEBF7') +

geom_line(color='#2171B5') +

labs(x = 'Age (years)', y = 'CHIKV Seroprev. (%)') +

scale_x_continuous(breaks = seq(5, 65, 5), expand = c(0, 5)) +

ylim(0,100) +

facet_wrap(~ cluster2, nrow=1, ncol=3) +

theme(text = element_text(size=20)) +

theme_few() +

theme(axis.title = element_text(size = 18), axis.text = element_text(size = 16), strip.text = element_text(size = 16) )

################################

# GLM DENV #

################################

# Model Regression Cluster1

#mod_glm7<-svyglm(igg_denv~age, design=svy.cluster1,family=quasibinomial(link = "logit"))

#summary(mod_glm7)

mod_glm7<-svyglm(igg_denv~log(age), design=svy.cluster1,family=quasibinomial(link = "cloglog"))

summary(mod_glm7)

testdata7 = data.frame(age = seq(5, 65, length = 100))

fits7 = predict.glm(mod_glm7, newdata=testdata7, type='response', se=T)

fits7$fit<-fits7$fit*100

fits7$se.fit<-fits7$se.fit*100

predicts7 = data.frame(testdata7, fits7) %>% mutate(lower = fit - 1.96*se.fit, upper = fit + 1.96*se.fit) %>% mutate(cluster="High")

predicts7$upper<-rec(predicts7$upper, rec="100:200=100; else=copy")

# Model Regression Cluster2

#mod_glm8<-svyglm(igg_denv~age, design=svy.cluster2,family=quasibinomial(link = "logit"))

#summary(mod_glm8)

mod_glm8<-svyglm(igg_denv~log(age), design=svy.cluster2,family=quasibinomial(link = "cloglog"))

summary(mod_glm8)

testdata8 = data.frame(age = seq(5, 65, length = 100))

fits8 = predict.glm(mod_glm8, newdata=testdata8, type='response', se=T)

fits8$fit<-fits8$fit*100

fits8$se.fit<-fits8$se.fit*100

predicts8 = data.frame(testdata8, fits8) %>% mutate(lower = fit - 1.96*se.fit, upper = fit + 1.96*se.fit) %>% mutate(cluster="Intermediate")

predicts8$upper<-rec(predicts8$upper, rec="100:200=100; else=copy")

# Model Regression Cluster3

#mod_glm9<-svyglm(igg_denv~age, design=svy.cluster3,family=quasibinomial(link = "logit"))

#summary(mod_glm9)

mod_glm9<-svyglm(igg_denv~log(age), design=svy.cluster3,family=quasibinomial(link = "cloglog"))

summary(mod_glm9)

testdata9 = data.frame(age = seq(5, 65, length = 100))

fits9 = predict.glm(mod_glm9, newdata=testdata9, type='response', se=T)

fits9$fit<-fits9$fit*100

fits9$se.fit<-fits9$se.fit*100

predicts9 = data.frame(testdata9, fits9) %>% mutate(lower = fit - 1.96*se.fit, upper = fit + 1.96*se.fit) %>% mutate(cluster="Deprived")

predicts9$upper<-rec(predicts9$upper, rec="100:200=100; else=copy")

predicts_glm3<-rbind(predicts7,predicts8,predicts9)

predicts_glm3$cluster2 <- factor(predicts_glm3$cluster, labels = c("High", "Intermediate", "Deprived"))

# Graphical Display

plot_mod_glm3_response = ggplot(aes(x=age,y=fit), data=predicts_glm3) +

geom_ribbon(aes(ymin = lower, ymax=upper), fill='#DEEBF7') +

geom_line(color='#2171B5') +

labs(x = 'Age (years)', y = 'DENV Seroprev. (%)') +

scale_x_continuous(breaks = seq(5, 65, 5), expand = c(0, 5)) +

ylim(0,100) +

facet_wrap(~ cluster2, nrow=1, ncol=3) +

theme_few() +

theme(axis.title = element_text(size = 18), axis.text = element_text(size = 16), strip.text = element_text(size = 16) )

##################

#### FIGURE 3 ####

##################

plot_grid(plot_mod_glm3_response, plot_mod_glm1_response,plot_mod_glm2_response, nrow=3, ncol=1)

#################################

## ASSOCIATED FACTORS ANALYSIS ##

#################################

##################################################

## Packages (libraries) for Data Analysis in R ##

##################################################

require(foreign)

library(prevalence)

require(survey)

require(prevalence)

library(MASS)

################

# READING DATA #

################

setwd("C:/Users/cflun/downloads/Cynthia Braga/Fatores associados")

dados1<-read.spss("dados_smv.sav")

dados2<-read.csv("dados_smv.csv", sep=";", dec=",")

dados3<-read.csv("dados_dom_smv.csv", sep=";", dec=",")

dados4<-read.csv("temp.csv", sep=";", dec=",")

######################################

# EFEITO DO DESENHO E PLANO AMOSTRAL #

######################################

#options(survey.lonely.psu = "adjust")

options(survey.lonely.psu = "certainty")

svy<-svydesign(id=~cod_setor+cod_dom, fpc=~fpc1+fpc2, strata=~cluster,weights=~peso, data=dados1, nest=TRUE)

summary(svy)

#options(survey.lonely.psu = "adjust")

options(survey.lonely.psu = "certainty")

svy<-svydesign(id=~cod_setor+cod_dom, fpc=~fpc1+fpc2, strata=~cluster,weights=~peso, data=dados2, nest=TRUE)

summary(svy)

#options(survey.lonely.psu = "adjust")

options(survey.lonely.psu = "certainty")

svy2<-svydesign(id=~cod_setor+cod_dom, fpc=~fpc1+fpc2, strata=~cluster,weights=~peso, data=dados3, nest=TRUE)

summary(svy2)

#options(survey.lonely.psu = "adjust")

options(survey.lonely.psu = "certainty")

svy3<-svydesign(id=~cod_setor+cod_dom, fpc=~fpc1+fpc2, strata=~cluster,weights=~peso, data=dados4, nest=TRUE)

summary(svy3)

###############################

# COMPARISONS IN EACH CLUSTER #

###############################

cluster1<-subset(dados2, cluster==1)

options(survey.lonely.psu = "certainty")

svy.cluster1<-svydesign(id=~cod_setor+cod_dom, fpc=~fpc1+fpc2, weights=~peso, data=cluster1, nest=TRUE)

summary(svy.cluster1)

cluster2<-subset(dados2, cluster==2)

svy.cluster2<-svydesign(id=~cod_setor+cod_dom, fpc=~fpc1+fpc2, weights=~peso, data=cluster2, nest=TRUE)

summary(svy.cluster2)

cluster3<-subset(dados2, cluster==3)

svy.cluster3<-svydesign(id=~cod_setor+cod_dom, fpc=~fpc1+fpc2, weights=~peso, data=cluster3, nest=TRUE)

summary(svy.cluster3)

source("longit.R")

##############

# TABLE 1 #

##############

# Número de domicílios

round(svytable(~cluster, design = svy2), digits=0)

round(prop.table(svytable(~cluster, design = svy2))*100, digits = 1)

svychisq(~cluster, svy2)

# Moradores por dormitório

round(svymean(~densi_mor, design = svy2), digits=2)

round(svyby(formula = ~densi_mor, by = ~cluster, design = svy2, FUN = svymean, na.rm=TRUE, vartype=c("se")), digits = 3)

#ANOVA (anova(svyglm(resident_count~cluster, design=svy2,family=gaussian(link = "identity")))

# só funciona a partir de duas variaveis

regTermTest(svyglm(densi_mor~cluster, design=svy2,family=gaussian(link = "identity")), "cluster")

# Tipo de domicílio

round(svytable(~type_house_1, design = svy2), digits=0)

round(prop.table(svytable(~type_house_1, design = svy2))*100, digits = 1)

round(svytable(~type_house_1+cluster, design = svy2), digits=0)

round(prop.table(svytable(~type_house_1+cluster, design = svy2), margin = 2)*100, digits = 1)

svychisq(~type_house_1+cluster, svy2)

# Esgotamento sanitário

round(svytable(~bathroom_waste_1, design = svy2), digits=0)

round(prop.table(svytable(~bathroom_waste_1, design = svy2))*100, digits = 1)

round(svytable(~bathroom_waste_1+cluster, design = svy2), digits=0)

round(prop.table(svytable(~bathroom_waste_1+cluster, design = svy2), margin = 2)*100, digits = 1)

svychisq(~bathroom_waste_1+cluster, svy2)

# Forma de abastecimento de água

round(svytable(~water_supply_1, design = svy2), digits=0)

round(prop.table(svytable(~water_supply_1, design = svy2))*100, digits = 1)

round(svytable(~water_supply_1+cluster, design = svy2), digits=0)

round(prop.table(svytable(~water_supply_1+cluster, design = svy2), margin = 2)*100, digits = 1)

svychisq(~water_supply_1+cluster, svy2)

# Abastecimento regular de água

round(svytable(~water_shortage, design = svy2), digits=0)

round(prop.table(svytable(~water_shortage, design = svy2))*100, digits = 1)

round(svytable(~water_shortage+cluster, design = svy2), digits=0)

round(prop.table(svytable(~water_shortage+cluster, design = svy2), margin = 2)*100, digits = 1)

svychisq(~water_shortage+cluster, svy2)

# Coleta de lixo realizada pelo serviço de limpeza pública

round(svytable(~garbage, design = svy2), digits=0)

round(prop.table(svytable(~garbage, design = svy2))*100, digits = 1)

round(svytable(~garbage+cluster, design = svy2), digits=0)

round(prop.table(svytable(~garbage+cluster, design = svy2), margin = 2)*100, digits = 1)

svychisq(~garbage+cluster, svy2)

# Sexo

round(svytable(~sex, design = svy2), digits=0)

round(prop.table(svytable(~sex, design = svy2))*100, digits = 1)

round(svytable(~sex+cluster, design = svy2), digits=0)

round(prop.table(svytable(~sex+cluster, design = svy2), margin = 2)*100, digits = 1)

svychisq(~sex+cluster, svy2)

# Renda do chefe

round(svytable(~income_chef, design = svy2), digits=0)

round(prop.table(svytable(~income_chef, design = svy2))*100, digits = 1)

round(svytable(~income_chef+cluster, design = svy2), digits=0)

round(prop.table(svytable(~income_chef+cluster, design = svy2), margin = 2)*100, digits = 1)

svychisq(~income_chef+cluster, svy2)

# Escolaridade do chefe

round(svytable(~education_chef, design = svy2), digits=0)

round(prop.table(svytable(~education_chef, design = svy2))*100, digits = 1)

round(svytable(~education_chef+cluster, design = svy2), digits=0)

round(prop.table(svytable(~education_chef+cluster, design = svy2), margin = 2)*100, digits = 1)

svychisq(~education_chef+cluster, svy2)

##############

# TABLE 6 #

##############

# CLUSTER 1

round(svytable(~numor_house_1, design = svy.cluster1), digits=0)

round(svytable(~numor_house_1+chikv_total, design = svy.cluster1), digits=0)

round(prop.table(svytable(~numor_house_1+chikv_total, design = svy.cluster1), margin = 1), digits = 3)

m1.cluster1<-svyglm(chikv_total~numor_house_1, design=svy.cluster1,family=quasibinomial(link = "logit"))

summary(m1.cluster1)

m1.cluster1<-svyglm(chikv_total~factor(numor_house_1), design=svy.cluster1,family=quasibinomial(link = "logit"))

summary(m1.cluster1)

or.svyglm(m1.cluster1)

round(svytable(~type_house_1, design = svy.cluster1), digits=0)

round(svytable(~type_house_1+chikv_total, design = svy.cluster1), digits=0)

round(prop.table(svytable(~type_house_1+chikv_total, design = svy.cluster1), margin = 1), digits = 3)

m2.cluster1<-svyglm(chikv_total~type_house_1, design=svy.cluster1,family=quasibinomial(link = "logit"))

summary(m2.cluster1)

m2.cluster1<-svyglm(chikv_total~factor(type_house_1), design=svy.cluster1,family=quasibinomial(link = "logit"))

summary(m2.cluster1)

or.svyglm(m2.cluster1)

round(svytable(~bathroom_waste_1, design = svy.cluster1), digits=0)

round(svytable(~bathroom_waste_1+chikv_total, design = svy.cluster1), digits=0)

round(prop.table(svytable(~bathroom_waste_1+chikv_total, design = svy.cluster1), margin = 1), digits = 3)

m3.cluster1<-svyglm(chikv_total~bathroom_waste_1, design=svy.cluster1,family=quasibinomial(link = "logit"))

summary(m3.cluster1)

m3.cluster1<-svyglm(chikv_total~factor(bathroom_waste_1), design=svy.cluster1,family=quasibinomial(link = "logit"))

summary(m3.cluster1)

or.svyglm(m3.cluster1)

round(svytable(~water_supply_1, design = svy.cluster1), digits=0)

round(svytable(~water_supply_1+chikv_total, design = svy.cluster1), digits=0)

round(prop.table(svytable(~water_supply_1+chikv_total, design = svy.cluster1), margin = 1), digits = 3)

m4.cluster1<-svyglm(chikv_total~water_supply_1, design=svy.cluster1,family=quasibinomial(link = "logit"))

summary(m4.cluster1)

m4.cluster1<-svyglm(chikv_total~factor(water_supply_1), design=svy.cluster1,family=quasibinomial(link = "logit"))

summary(m4.cluster1)

or.svyglm(m4.cluster1)

round(svytable(~water_shortage, design = svy.cluster1), digits=0)

round(svytable(~water_shortage+chikv_total, design = svy.cluster1), digits=0)

round(prop.table(svytable(~water_shortage+chikv_total, design = svy.cluster1), margin = 1), digits = 3)

m5.cluster1<-svyglm(chikv_total~water_shortage, design=svy.cluster1,family=quasibinomial(link = "logit"))

summary(m5.cluster1)

m5.cluster1<-svyglm(chikv_total~factor(water_shortage), design=svy.cluster1,family=quasibinomial(link = "logit"))

summary(m5.cluster1)

or.svyglm(m5.cluster1)

round(svytable(~garbage_1, design = svy.cluster1), digits=0)

round(svytable(~garbage_1+chikv_total, design = svy.cluster1), digits=0)

round(prop.table(svytable(~garbage_1+chikv_total, design = svy.cluster1), margin = 1), digits = 3)

m6.cluster1<-svyglm(chikv_total~garbage_1, design=svy.cluster1,family=quasibinomial(link = "logit"))

summary(m6.cluster1)

m6.cluster1<-svyglm(chikv_total~factor(garbage_1), design=svy.cluster1,family=quasibinomial(link = "logit"))

summary(m6.cluster1)

or.svyglm(m6.cluster1)

round(svytable(~income_chef, design = svy.cluster1), digits=0)

round(svytable(~income_chef+chikv_total, design = svy.cluster1), digits=0)

round(prop.table(svytable(~income_chef+chikv_total, design = svy.cluster1), margin = 1), digits = 3)

m7.cluster1<-svyglm(chikv_total~income_chef, design=svy.cluster1,family=quasibinomial(link = "logit"))

summary(m7.cluster1)

m7.cluster1<-svyglm(chikv_total~factor(income_chef), design=svy.cluster1,family=quasibinomial(link = "logit"))

summary(m7.cluster1)

or.svyglm(m7.cluster1)

round(svytable(~education_chef, design = svy.cluster1), digits=0)

round(svytable(~education_chef+chikv_total, design = svy.cluster1), digits=0)

round(prop.table(svytable(~education_chef+chikv_total, design = svy.cluster1), margin = 1), digits = 3)

m8.cluster1<-svyglm(chikv_total~education_chef, design=svy.cluster1,family=quasibinomial(link = "logit"))

summary(m8.cluster1)

m8.cluster1<-svyglm(chikv_total~factor(education_chef), design=svy.cluster1,family=quasibinomial(link = "logit"))

summary(m8.cluster1)

or.svyglm(m8.cluster1)

round(svytable(~race_chef_2, design = svy.cluster1), digits=0)

round(svytable(~race_chef_2+chikv_total, design = svy.cluster1), digits=0)

round(prop.table(svytable(~race_chef_2+chikv_total, design = svy.cluster1), margin = 1), digits = 3)

m9.cluster1<-svyglm(chikv_total~race_chef_2, design=svy.cluster1,family=quasibinomial(link = "logit"))

summary(m9.cluster1)

m9.cluster1<-svyglm(chikv_total~factor(race_chef_2), design=svy.cluster1,family=quasibinomial(link = "logit"))

summary(m9.cluster1)

or.svyglm(m9.cluster1)

# CLUSTER 2

round(svytable(~numor_house_1, design = svy.cluster2), digits=0)

round(svytable(~numor_house_1+chikv_total, design = svy.cluster2), digits=0)

round(prop.table(svytable(~numor_house_1+chikv_total, design = svy.cluster2), margin = 1), digits = 3)

m1.cluster2<-svyglm(chikv_total~numor_house_1, design=svy.cluster2,family=quasibinomial(link = "logit"))

summary(m1.cluster2)

m1.cluster2<-svyglm(chikv_total~factor(numor_house_1), design=svy.cluster2,family=quasibinomial(link = "logit"))

summary(m1.cluster2)

or.svyglm(m1.cluster2)

round(svytable(~type_house_1, design = svy.cluster2), digits=0)

round(svytable(~type_house_1+chikv_total, design = svy.cluster2), digits=0)

round(prop.table(svytable(~type_house_1+chikv_total, design = svy.cluster2), margin = 1), digits = 3)

m2.cluster2<-svyglm(chikv_total~type_house_1, design=svy.cluster2,family=quasibinomial(link = "logit"))

summary(m2.cluster2)

m2.cluster2<-svyglm(chikv_total~factor(type_house_1), design=svy.cluster2,family=quasibinomial(link = "logit"))

summary(m2.cluster2)

or.svyglm(m2.cluster2)

round(svytable(~bathroom_waste_1, design = svy.cluster2), digits=0)

round(svytable(~bathroom_waste_1+chikv_total, design = svy.cluster2), digits=0)

round(prop.table(svytable(~bathroom_waste_1+chikv_total, design = svy.cluster2), margin = 1), digits = 3)

m3.cluster2<-svyglm(chikv_total~bathroom_waste_1, design=svy.cluster2,family=quasibinomial(link = "logit"))

summary(m3.cluster2)

m3.cluster2<-svyglm(chikv_total~factor(bathroom_waste_1), design=svy.cluster2,family=quasibinomial(link = "logit"))

summary(m3.cluster2)

or.svyglm(m3.cluster2)

round(svytable(~water_supply_1, design = svy.cluster2), digits=0)

round(svytable(~water_supply_1+chikv_total, design = svy.cluster2), digits=0)

round(prop.table(svytable(~water_supply_1+chikv_total, design = svy.cluster2), margin = 1), digits = 3)

m4.cluster2<-svyglm(chikv_total~water_supply_1, design=svy.cluster2,family=quasibinomial(link = "logit"))

summary(m4.cluster2)

m4.cluster2<-svyglm(chikv_total~factor(water_supply_1), design=svy.cluster2,family=quasibinomial(link = "logit"))

summary(m4.cluster2)

or.svyglm(m4.cluster2)

round(svytable(~water_shortage, design = svy.cluster2), digits=0)

round(svytable(~water_shortage+chikv_total, design = svy.cluster2), digits=0)

round(prop.table(svytable(~water_shortage+chikv_total, design = svy.cluster2), margin = 1), digits = 3)

m5.cluster2<-svyglm(chikv_total~water_shortage, design=svy.cluster2,family=quasibinomial(link = "logit"))

summary(m5.cluster2)

m5.cluster2<-svyglm(chikv_total~factor(water_shortage), design=svy.cluster2,family=quasibinomial(link = "logit"))

summary(m5.cluster2)

or.svyglm(m5.cluster2)

round(svytable(~garbage_1, design = svy.cluster2), digits=0)

round(svytable(~garbage_1+chikv_total, design = svy.cluster2), digits=0)

round(prop.table(svytable(~garbage_1+chikv_total, design = svy.cluster2), margin = 1), digits = 3)

m6.cluster2<-svyglm(chikv_total~garbage_1, design=svy.cluster2,family=quasibinomial(link = "logit"))

summary(m6.cluster2)

m6.cluster2<-svyglm(chikv_total~factor(garbage_1), design=svy.cluster2,family=quasibinomial(link = "logit"))

summary(m6.cluster2)

or.svyglm(m6.cluster2)

round(svytable(~income_chef, design = svy.cluster2), digits=0)

round(svytable(~income_chef+chikv_total, design = svy.cluster2), digits=0)

round(prop.table(svytable(~income_chef+chikv_total, design = svy.cluster2), margin = 1), digits = 3)

m7.cluster2<-svyglm(chikv_total~income_chef, design=svy.cluster2,family=quasibinomial(link = "logit"))

summary(m7.cluster2)

m7.cluster2<-svyglm(chikv_total~factor(income_chef), design=svy.cluster2,family=quasibinomial(link = "logit"))

summary(m7.cluster2)

or.svyglm(m7.cluster2)

round(svytable(~education_chef, design = svy.cluster2), digits=0)

round(svytable(~education_chef+chikv_total, design = svy.cluster2), digits=0)

round(prop.table(svytable(~education_chef+chikv_total, design = svy.cluster2), margin = 1), digits = 3)

m8.cluster2<-svyglm(chikv_total~education_chef, design=svy.cluster2,family=quasibinomial(link = "logit"))

summary(m8.cluster2)

m8.cluster2<-svyglm(chikv_total~factor(education_chef), design=svy.cluster2,family=quasibinomial(link = "logit"))

summary(m8.cluster2)

or.svyglm(m8.cluster2)

round(svytable(~race_chef_2, design = svy.cluster2), digits=0)

round(svytable(~race_chef_2+chikv_total, design = svy.cluster2), digits=0)

round(prop.table(svytable(~race_chef_2+chikv_total, design = svy.cluster2), margin = 1), digits = 3)

m9.cluster2<-svyglm(chikv_total~race_chef_2, design=svy.cluster2,family=quasibinomial(link = "logit"))

summary(m9.cluster2)

m9.cluster2<-svyglm(chikv_total~factor(race_chef_2), design=svy.cluster2,family=quasibinomial(link = "logit"))

summary(m9.cluster2)

or.svyglm(m9.cluster2)

# CLUSTER 3

round(svytable(~numor_house_1, design = svy.cluster3), digits=0)

round(svytable(~numor_house_1+chikv_total, design = svy.cluster3), digits=0)

round(prop.table(svytable(~numor_house_1+chikv_total, design = svy.cluster3), margin = 1), digits = 3)

m1.cluster3<-svyglm(chikv_total~numor_house_1, design=svy.cluster3,family=quasibinomial(link = "logit"))

summary(m1.cluster3)

m1.cluster3<-svyglm(chikv_total~factor(numor_house_1), design=svy.cluster3,family=quasibinomial(link = "logit"))

summary(m1.cluster3)

or.svyglm(m1.cluster3)

round(svytable(~type_house_1, design = svy.cluster3), digits=0)

round(svytable(~type_house_1+chikv_total, design = svy.cluster3), digits=0)

round(prop.table(svytable(~type_house_1+chikv_total, design = svy.cluster3), margin = 1), digits = 3)

m2.cluster3<-svyglm(chikv_total~type_house_1, design=svy.cluster3,family=quasibinomial(link = "logit"))

summary(m2.cluster3)

m2.cluster3<-svyglm(chikv_total~factor(type_house_1), design=svy.cluster3,family=quasibinomial(link = "logit"))

summary(m2.cluster3)

or.svyglm(m2.cluster3)

round(svytable(~bathroom_waste_1, design = svy.cluster3), digits=0)

round(svytable(~bathroom_waste_1+chikv_total, design = svy.cluster3), digits=0)

round(prop.table(svytable(~bathroom_waste_1+chikv_total, design = svy.cluster3), margin = 1), digits = 3)

m3.cluster3<-svyglm(chikv_total~bathroom_waste_1, design=svy.cluster3,family=quasibinomial(link = "logit"))

summary(m3.cluster3)

m3.cluster3<-svyglm(chikv_total~factor(bathroom_waste_1), design=svy.cluster3,family=quasibinomial(link = "logit"))

summary(m3.cluster3)

or.svyglm(m3.cluster3)

round(svytable(~water_supply_1, design = svy.cluster3), digits=0)

round(svytable(~water_supply_1+chikv_total, design = svy.cluster3), digits=0)

round(prop.table(svytable(~water_supply_1+chikv_total, design = svy.cluster3), margin = 1), digits = 3)

m4.cluster3<-svyglm(chikv_total~factor(water_supply_1), design=svy.cluster3,family=quasibinomial(link = "logit"))

summary(m4.cluster3)

m4.cluster3<-svyglm(chikv_total~factor(water_supply_1), design=svy.cluster3,family=quasibinomial(link = "logit"))

summary(m4.cluster3)

or.svyglm(m4.cluster3)

round(svytable(~water_shortage, design = svy.cluster3), digits=0)

round(svytable(~water_shortage+chikv_total, design = svy.cluster3), digits=0)

round(prop.table(svytable(~water_shortage+chikv_total, design = svy.cluster3), margin = 1), digits = 3)

m5.cluster3<-svyglm(chikv_total~water_shortage, design=svy.cluster3,family=quasibinomial(link = "logit"))

summary(m5.cluster3)

m5.cluster3<-svyglm(chikv_total~factor(water_shortage), design=svy.cluster3,family=quasibinomial(link = "logit"))

summary(m5.cluster3)

or.svyglm(m5.cluster3)

round(svytable(~garbage_1, design = svy.cluster3), digits=0)

round(svytable(~garbage_1+chikv_total, design = svy.cluster3), digits=0)

round(prop.table(svytable(~garbage_1+chikv_total, design = svy.cluster3), margin = 1), digits = 3)

m6.cluster3<-svyglm(chikv_total~garbage_1, design=svy.cluster3,family=quasibinomial(link = "logit"))

summary(m6.cluster3)

m6.cluster3<-svyglm(chikv_total~factor(garbage_1), design=svy.cluster3,family=quasibinomial(link = "logit"))

summary(m6.cluster3)

or.svyglm(m6.cluster3)

round(svytable(~income_chef, design = svy.cluster3), digits=0)

round(svytable(~income_chef+chikv_total, design = svy.cluster3), digits=0)

round(prop.table(svytable(~income_chef+chikv_total, design = svy.cluster3), margin = 1), digits = 3)

m7.cluster3<-svyglm(chikv_total~income_chef, design=svy.cluster3,family=quasibinomial(link = "logit"))

summary(m7.cluster3)

m7.cluster3<-svyglm(chikv_total~factor(income_chef), design=svy.cluster3,family=quasibinomial(link = "logit"))

summary(m7.cluster3)

or.svyglm(m7.cluster3)

round(svytable(~education_chef, design = svy.cluster3), digits=0)

round(svytable(~education_chef+chikv_total, design = svy.cluster3), digits=0)

round(prop.table(svytable(~education_chef+chikv_total, design = svy.cluster3), margin = 1), digits = 3)

m8.cluster3<-svyglm(chikv_total~education_chef, design=svy.cluster3,family=quasibinomial(link = "logit"))

summary(m8.cluster3)

m8.cluster3<-svyglm(chikv_total~factor(education_chef), design=svy.cluster3,family=quasibinomial(link = "logit"))

summary(m8.cluster3)

or.svyglm(m8.cluster3)

round(svytable(~race_chef_2, design = svy.cluster3), digits=0)

round(svytable(~race_chef_2+chikv_total, design = svy.cluster3), digits=0)

round(prop.table(svytable(~race_chef_2+chikv_total, design = svy.cluster3), margin = 1), digits = 3)

m9.cluster3<-svyglm(chikv_total~race_chef_2, design=svy.cluster3,family=quasibinomial(link = "logit"))

summary(m9.cluster3)

m9.cluster3<-svyglm(chikv_total~factor(race_chef_2), design=svy.cluster3,family=quasibinomial(link = "logit"))

summary(m9.cluster3)

or.svyglm(m9.cluster3)

##############

# TABLE 7 #

##############

# CLUSTER 1

round(svytable(~sex, design = svy.cluster1), digits=0)

round(svytable(~sex+chikv_total, design = svy.cluster1), digits=0)

round(prop.table(svytable(~sex+chikv_total, design = svy.cluster1), margin = 1), digits = 3)

m10.cluster1<-svyglm(chikv_total~sex, design=svy.cluster1,family=quasibinomial(link = "logit"))

summary(m10.cluster1)

m10.cluster1<-svyglm(chikv_total~factor(sex), design=svy.cluster1,family=quasibinomial(link = "logit"))

summary(m10.cluster1)

or.svyglm(m10.cluster1)

round(svytable(~idgrupo4, design = svy.cluster1), digits=0)

round(svytable(~idgrupo4+chikv_total, design = svy.cluster1), digits=0)

round(prop.table(svytable(~idgrupo4+chikv_total, design = svy.cluster1), margin = 1), digits = 3)

m11.cluster1<-svyglm(chikv_total~idgrupo4, design=svy.cluster1,family=quasibinomial(link = "logit"))

summary(m11.cluster1)

m11.cluster1<-svyglm(chikv_total~factor(idgrupo4), design=svy.cluster1,family=quasibinomial(link = "logit"))

summary(m11.cluster1)

or.svyglm(m11.cluster1)

round(svytable(~race_ind_2, design = svy.cluster1), digits=0)

round(svytable(~race_ind_2+chikv_total, design = svy.cluster1), digits=0)

round(prop.table(svytable(~race_ind_2+chikv_total, design = svy.cluster1), margin = 1), digits = 3)

m12.cluster1<-svyglm(chikv_total~race_ind_2, design=svy.cluster1,family=quasibinomial(link = "logit"))

summary(m12.cluster1)

m12.cluster1<-svyglm(chikv_total~factor(race_ind_2), design=svy.cluster1,family=quasibinomial(link = "logit"))

summary(m12.cluster1)

or.svyglm(m12.cluster1)

round(svytable(~education_ind, design = svy.cluster1), digits=0)

round(svytable(~education_ind+chikv_total, design = svy.cluster1), digits=0)

round(prop.table(svytable(~education_ind+chikv_total, design = svy.cluster1), margin = 1), digits = 3)

m13.cluster1<-svyglm(chikv_total~education_ind, design=svy.cluster1,family=quasibinomial(link = "logit"))

summary(m13.cluster1)

m13.cluster1<-svyglm(chikv_total~factor(education_ind), design=svy.cluster1,family=quasibinomial(link = "logit"))

summary(m13.cluster1)

or.svyglm(m13.cluster1)

round(svytable(~income_ind_2, design = svy.cluster1), digits=0)

round(svytable(~income_ind_2+chikv_total, design = svy.cluster1), digits=0)

round(prop.table(svytable(~income_ind_2+chikv_total, design = svy.cluster1), margin = 1), digits = 3)

#m14.cluster1<-svyglm(chikv_total~income_ind_2, design=svy.cluster1,family=quasibinomial(link = "logit"))

#summary(m14.cluster1)

#m14.cluster1<-svyglm(chikv_total~factor(income_ind_2), design=svy.cluster1,family=quasibinomial(link = "logit"))

#summary(m14.cluster1)

#or.svyglm(m14.cluster1)

round(svytable(~mobility, design = svy.cluster1), digits=0)

round(svytable(~mobility+chikv_total, design = svy.cluster1), digits=0)

round(prop.table(svytable(~mobility+chikv_total, design = svy.cluster1), margin = 1), digits = 3)

m15.cluster1<-svyglm(chikv_total~mobility, design=svy.cluster1,family=quasibinomial(link = "logit"))

summary(m15.cluster1)

m15.cluster1<-svyglm(chikv_total~factor(mobility), design=svy.cluster1,family=quasibinomial(link = "logit"))

summary(m15.cluster1)

or.svyglm(m15.cluster1)

round(svytable(~repellent, design = svy.cluster1), digits=0)

round(svytable(~repellent+chikv_total, design = svy.cluster1), digits=0)

round(prop.table(svytable(~repellent+chikv_total, design = svy.cluster1), margin = 1), digits = 3)

m16.cluster1<-svyglm(chikv_total~repellent, design=svy.cluster1,family=quasibinomial(link = "logit"))

summary(m16.cluster1)

m16.cluster1<-svyglm(chikv_total~factor(repellent), design=svy.cluster1,family=quasibinomial(link = "logit"))

summary(m16.cluster1)

or.svyglm(m16.cluster1)

round(svytable(~dengue_exp, design = svy.cluster1), digits=0)

round(svytable(~dengue_exp+chikv_total, design = svy.cluster1), digits=0)

round(prop.table(svytable(~dengue_exp+chikv_total, design = svy.cluster1), margin = 1), digits = 3)

m17.cluster1<-svyglm(chikv_total~dengue_exp, design=svy.cluster1,family=quasibinomial(link = "logit"))

summary(m17.cluster1)

m17.cluster1<-svyglm(chikv_total~factor(dengue_exp), design=svy.cluster1,family=quasibinomial(link = "logit"))

summary(m17.cluster1)

or.svyglm(m17.cluster1)

# CLUSTER 2

round(svytable(~sex, design = svy.cluster2), digits=0)

round(svytable(~sex+chikv_total, design = svy.cluster2), digits=0)

round(prop.table(svytable(~sex+chikv_total, design = svy.cluster2), margin = 1), digits = 3)

m10.cluster2<-svyglm(chikv_total~sex, design=svy.cluster2,family=quasibinomial(link = "logit"))

summary(m10.cluster2)

m10.cluster2<-svyglm(chikv_total~factor(sex), design=svy.cluster2,family=quasibinomial(link = "logit"))

summary(m10.cluster2)

or.svyglm(m10.cluster2)

round(svytable(~idgrupo4, design = svy.cluster2), digits=0)

round(svytable(~idgrupo4+chikv_total, design = svy.cluster2), digits=0)

round(prop.table(svytable(~idgrupo4+chikv_total, design = svy.cluster2), margin = 1), digits = 3)

m11.cluster2<-svyglm(chikv_total~idgrupo4, design=svy.cluster2,family=quasibinomial(link = "logit"))

summary(m11.cluster2)

m11.cluster2<-svyglm(chikv_total~factor(idgrupo4), design=svy.cluster2,family=quasibinomial(link = "logit"))

summary(m11.cluster2)

or.svyglm(m11.cluster2)

round(svytable(~race_ind_2, design = svy.cluster2), digits=0)

round(svytable(~race_ind_2+chikv_total, design = svy.cluster2), digits=0)

round(prop.table(svytable(~race_ind_2+chikv_total, design = svy.cluster2), margin = 1), digits = 3)

m12.cluster2<-svyglm(chikv_total~race_ind_2, design=svy.cluster2,family=quasibinomial(link = "logit"))

summary(m12.cluster2)

m12.cluster2<-svyglm(chikv_total~factor(race_ind_2), design=svy.cluster2,family=quasibinomial(link = "logit"))

summary(m12.cluster2)

or.svyglm(m12.cluster2)

round(svytable(~education_ind, design = svy.cluster2), digits=0)

round(svytable(~education_ind+chikv_total, design = svy.cluster2), digits=0)

round(prop.table(svytable(~education_ind+chikv_total, design = svy.cluster2), margin = 1), digits = 3)

m13.cluster2<-svyglm(chikv_total~education_ind, design=svy.cluster2,family=quasibinomial(link = "logit"))

summary(m13.cluster2)

m13.cluster2<-svyglm(chikv_total~factor(education_ind), design=svy.cluster2,family=quasibinomial(link = "logit"))

summary(m13.cluster2)

or.svyglm(m13.cluster2)

round(svytable(~income_ind_2, design = svy.cluster2), digits=0)

round(svytable(~income_ind_2+chikv_total, design = svy.cluster2), digits=0)

round(prop.table(svytable(~income_ind_2+chikv_total, design = svy.cluster2), margin = 1), digits = 3)

#m14.cluster2<-svyglm(chikv_total~income_ind_2, design=svy.cluster2,family=quasibinomial(link = "logit"))

#summary(m14.cluster2)

#m14.cluster2<-svyglm(chikv_total~factor(income_ind_2), design=svy.cluster2,family=quasibinomial(link = "logit"))

#summary(m14.cluster2)

#or.svyglm(m14.cluster2)

round(svytable(~mobility, design = svy.cluster2), digits=0)

round(svytable(~mobility+chikv_total, design = svy.cluster2), digits=0)

round(prop.table(svytable(~mobility+chikv_total, design = svy.cluster2), margin = 1), digits = 3)

m15.cluster2<-svyglm(chikv_total~mobility, design=svy.cluster2,family=quasibinomial(link = "logit"))

summary(m15.cluster2)

m15.cluster2<-svyglm(chikv_total~factor(mobility), design=svy.cluster2,family=quasibinomial(link = "logit"))

summary(m15.cluster2)

or.svyglm(m15.cluster2)

round(svytable(~repellent, design = svy.cluster2), digits=0)

round(svytable(~repellent+chikv_total, design = svy.cluster2), digits=0)

round(prop.table(svytable(~repellent+chikv_total, design = svy.cluster2), margin = 1), digits = 3)

m16.cluster2<-svyglm(chikv_total~repellent, design=svy.cluster2,family=quasibinomial(link = "logit"))

summary(m16.cluster2)

m16.cluster2<-svyglm(chikv_total~factor(repellent), design=svy.cluster2,family=quasibinomial(link = "logit"))

summary(m16.cluster2)

or.svyglm(m16.cluster2)

round(svytable(~dengue_exp, design = svy.cluster2), digits=0)

round(svytable(~dengue_exp+chikv_total, design = svy.cluster2), digits=0)

round(prop.table(svytable(~dengue_exp+chikv_total, design = svy.cluster2), margin = 1), digits = 3)

m17.cluster2<-svyglm(chikv_total~dengue_exp, design=svy.cluster2,family=quasibinomial(link = "logit"))

summary(m17.cluster2)

m17.cluster2<-svyglm(chikv_total~factor(dengue_exp), design=svy.cluster2,family=quasibinomial(link = "logit"))

summary(m17.cluster2)

or.svyglm(m17.cluster2)

# CLUSTER 3

round(svytable(~sex, design = svy.cluster3), digits=0)

round(svytable(~sex+chikv_total, design = svy.cluster3), digits=0)

round(prop.table(svytable(~sex+chikv_total, design = svy.cluster3), margin = 1), digits = 3)

m10.cluster3<-svyglm(chikv_total~sex, design=svy.cluster3,family=quasibinomial(link = "logit"))

summary(m10.cluster3)

m10.cluster3<-svyglm(chikv_total~factor(sex), design=svy.cluster3,family=quasibinomial(link = "logit"))

summary(m10.cluster3)

or.svyglm(m10.cluster3)

round(svytable(~idgrupo4, design = svy.cluster3), digits=0)

round(svytable(~idgrupo4+chikv_total, design = svy.cluster3), digits=0)

round(prop.table(svytable(~idgrupo4+chikv_total, design = svy.cluster3), margin = 1), digits = 3)

m11.cluster3<-svyglm(chikv_total~idgrupo4, design=svy.cluster3,family=quasibinomial(link = "logit"))

summary(m11.cluster3)

m11.cluster3<-svyglm(chikv_total~factor(idgrupo4), design=svy.cluster3,family=quasibinomial(link = "logit"))

summary(m11.cluster3)

or.svyglm(m11.cluster3)

round(svytable(~race_ind_2, design = svy.cluster3), digits=0)

round(svytable(~race_ind_2+chikv_total, design = svy.cluster3), digits=0)

round(prop.table(svytable(~race_ind_2+chikv_total, design = svy.cluster3), margin = 1), digits = 3)

m12.cluster3<-svyglm(chikv_total~race_ind_2, design=svy.cluster3,family=quasibinomial(link = "logit"))

summary(m12.cluster3)

m12.cluster3<-svyglm(chikv_total~factor(race_ind_2), design=svy.cluster3,family=quasibinomial(link = "logit"))

summary(m12.cluster3)

or.svyglm(m12.cluster3)

round(svytable(~education_ind, design = svy.cluster3), digits=0)

round(svytable(~education_ind+chikv_total, design = svy.cluster3), digits=0)

round(prop.table(svytable(~education_ind+chikv_total, design = svy.cluster3), margin = 1), digits = 3)

m13.cluster3<-svyglm(chikv_total~education_ind, design=svy.cluster3,family=quasibinomial(link = "logit"))

summary(m13.cluster3)

m13.cluster3<-svyglm(chikv_total~factor(education_ind), design=svy.cluster3,family=quasibinomial(link = "logit"))

summary(m13.cluster3)

or.svyglm(m13.cluster3)

round(svytable(~income_ind_2, design = svy.cluster3), digits=0)

round(svytable(~income_ind_2+chikv_total, design = svy.cluster3), digits=0)

round(prop.table(svytable(~income_ind_2+chikv_total, design = svy.cluster3), margin = 1), digits = 3)

#m14.cluster3<-svyglm(chikv_total~income_ind_2, design=svy.cluster3,family=quasibinomial(link = "logit"))

#summary(m14.cluster3)

#m14.cluster3<-svyglm(chikv_total~factor(income_ind_2), design=svy.cluster3,family=quasibinomial(link = "logit"))

#summary(m14.cluster3)

#or.svyglm(m14.cluster3)

round(svytable(~mobility, design = svy.cluster3), digits=0)

round(svytable(~mobility+chikv_total, design = svy.cluster3), digits=0)

round(prop.table(svytable(~mobility+chikv_total, design = svy.cluster3), margin = 1), digits = 3)

m15.cluster3<-svyglm(chikv_total~mobility, design=svy.cluster3,family=quasibinomial(link = "logit"))

summary(m15.cluster3)

m15.cluster3<-svyglm(chikv_total~factor(mobility), design=svy.cluster3,family=quasibinomial(link = "logit"))

summary(m15.cluster3)

or.svyglm(m15.cluster3)

round(svytable(~repellent, design = svy.cluster3), digits=0)

round(svytable(~repellent+chikv_total, design = svy.cluster3), digits=0)

round(prop.table(svytable(~repellent+chikv_total, design = svy.cluster3), margin = 1), digits = 3)

m16.cluster3<-svyglm(chikv_total~repellent, design=svy.cluster3,family=quasibinomial(link = "logit"))

summary(m16.cluster3)

m16.cluster3<-svyglm(chikv_total~factor(repellent), design=svy.cluster3,family=quasibinomial(link = "logit"))

summary(m16.cluster3)

or.svyglm(m16.cluster3)

round(svytable(~dengue_exp, design = svy.cluster3), digits=0)

round(svytable(~dengue_exp+chikv_total, design = svy.cluster3), digits=0)

round(prop.table(svytable(~dengue_exp+chikv_total, design = svy.cluster3), margin = 1), digits = 3)

m17.cluster3<-svyglm(chikv_total~dengue_exp, design=svy.cluster3,family=quasibinomial(link = "logit"))

summary(m17.cluster3)

m17.cluster3<-svyglm(chikv_total~factor(dengue_exp), design=svy.cluster3,family=quasibinomial(link = "logit"))

summary(m17.cluster3)

or.svyglm(m17.cluster3)

##############

# TABLE 8 #

##############

round(svytable(~cluster, design = svy), digits=0)

# ANALISE MULTIVARIADA (INCLUINDO APENAS AS VARIAVEIS COM P<=0,25 NA ANALISE BIVARIADA POR BLOCOS) #

# CLUSTER 1

mf1.b1.cluster1<-stepAIC(svyglm(chikv_total~factor(numor_house_1)+factor(type_house_1)+factor(bathroom_waste_1)+factor(water_shortage)+factor(income_chef)+factor(education_chef), design=svy.cluster1,family=quasibinomial(link = "logit")), direction = c("backward"))

summary(mf1.b1.cluster1)

mf1.b2.cluster1<-stepAIC(svyglm(chikv_total~factor(sex)+factor(idgrupo4)+factor(race_ind_2)+factor(education_ind)+factor(mobility)+factor(repellent)+factor(dengue_exp), design=svy.cluster1,family=quasibinomial(link = "logit")), direction = c("backward"))

summary(mf1.b2.cluster1)

mf1.b2.cluster1<-stepAIC(svyglm(chikv_total~factor(education_ind)+factor(repellent)+factor(dengue_exp), design=svy.cluster1,family=quasibinomial(link = "logit")), direction = c("backward"))

summary(mf1.b2.cluster1)

mf1.b2.cluster1<-stepAIC(svyglm(chikv_total~factor(education_ind)+factor(dengue_exp), design=svy.cluster1,family=quasibinomial(link = "logit")), direction = c("backward"))

summary(mf1.b2.cluster1)

mf1.b.cluster1<-svyglm(chikv_total~factor(type_house_1)+factor(bathroom_waste_1)+factor(education_chef)+factor(education_ind)+factor(dengue_exp), design=svy.cluster1,family=quasibinomial(link = "logit"))

or.svyglm(mf1.b.cluster1)

# CLUSTER 2

mf1.b1.cluster2<-stepAIC(svyglm(chikv_total~factor(numor_house_1)+factor(type_house_1)+factor(water_supply_1)+factor(water_shortage)+factor(income_chef)+factor(education_chef), design=svy.cluster2,family=quasibinomial(link = "logit")),direction = c("backward"))

summary(mf1.b1.cluster2)

mf1.b1.cluster2<-stepAIC(svyglm(chikv_total~factor(type_house_1)+factor(education_chef), design=svy.cluster2,family=quasibinomial(link = "logit")),direction = c("backward"))

summary(mf1.b1.cluster2)

mf1.b2.cluster2<-stepAIC(svyglm(chikv_total~factor(idgrupo4)+factor(education_ind)+factor(mobility)+factor(dengue_exp), design=svy.cluster2,family=quasibinomial(link = "logit")),direction = c("backward"))

summary(mf1.b2.cluster2)

mf1.b.cluster2<-svyglm(chikv_total~factor(type_house_1)+factor(education_chef)+factor(education_ind)+factor(mobility)+factor(dengue_exp), design=svy.cluster2,family=quasibinomial(link = "logit"))

or.svyglm(mf1.b.cluster2)

# CLUSTER 3

mf1.b1.cluster3<-stepAIC(svyglm(chikv_total~factor(bathroom_waste_1)+factor(garbage_1)+factor(income_chef)+factor(education_chef), design=svy.cluster3,family=quasibinomial(link = "logit")),direction = c("backward"))

summary(mf1.b1.cluster3)

mf1.b1.cluster3<-stepAIC(svyglm(chikv_total~factor(income_chef)+factor(education_chef), design=svy.cluster3,family=quasibinomial(link = "logit")),direction = c("backward"))

summary(mf1.b1.cluster3)

mf1.b1.cluster3<-stepAIC(svyglm(chikv_total~factor(income_chef), design=svy.cluster3,family=quasibinomial(link = "logit")),direction = c("backward"))

summary(mf1.b1.cluster3)

mf1.b2.cluster3<-stepAIC(svyglm(chikv_total~factor(idgrupo4)+factor(education_ind)+factor(mobility)+factor(repellent)+factor(dengue_exp), design=svy.cluster3,family=quasibinomial(link = "logit")),direction = c("backward"))

summary(mf1.b2.cluster3)

mf1.b2.cluster3<-stepAIC(svyglm(chikv_total~factor(education_ind)+factor(mobility)+factor(dengue_exp), design=svy.cluster3,family=quasibinomial(link = "logit")),direction = c("backward"))

summary(mf1.b2.cluster3)

mf1.b.cluster3<-svyglm(chikv_total~factor(income_chef)+factor(mobility)+factor(dengue_exp), design=svy.cluster3,family=quasibinomial(link = "logit"))

or.svyglm(mf1.b.cluster3)

##############

# TABLE 9 #

##############

# CLUSTER 1

round(svytable(~numor_house_1, design = svy.cluster1), digits=0)

round(svytable(~numor_house_1+igg_zikv_total, design = svy.cluster1), digits=0)

round(prop.table(svytable(~numor_house_1+igg_zikv_total, design = svy.cluster1), margin = 1), digits = 3)

m18.cluster1<-svyglm(igg_zikv_total~numor_house_1, design=svy.cluster1,family=quasibinomial(link = "logit"))

summary(m18.cluster1)

m18.cluster1<-svyglm(igg_zikv_total~factor(numor_house_1), design=svy.cluster1,family=quasibinomial(link = "logit"))

summary(m18.cluster1)

or.svyglm(m18.cluster1)

round(svytable(~type_house_1, design = svy.cluster1), digits=0)

round(svytable(~type_house_1+igg_zikv_total, design = svy.cluster1), digits=0)

round(prop.table(svytable(~type_house_1+igg_zikv_total, design = svy.cluster1), margin = 1), digits = 3)

m19.cluster1<-svyglm(igg_zikv_total~type_house_1, design=svy.cluster1,family=quasibinomial(link = "logit"))

summary(m19.cluster1)

m19.cluster1<-svyglm(igg_zikv_total~factor(type_house_1), design=svy.cluster1,family=quasibinomial(link = "logit"))

summary(m19.cluster1)

or.svyglm(m19.cluster1)

round(svytable(~bathroom_waste_1, design = svy.cluster1), digits=0)

round(svytable(~bathroom_waste_1+igg_zikv_total, design = svy.cluster1), digits=0)

round(prop.table(svytable(~bathroom_waste_1+igg_zikv_total, design = svy.cluster1), margin = 1), digits = 3)

m20.cluster1<-svyglm(igg_zikv_total~bathroom_waste_1, design=svy.cluster1,family=quasibinomial(link = "logit"))

summary(m20.cluster1)

m20.cluster1<-svyglm(igg_zikv_total~factor(bathroom_waste_1), design=svy.cluster1,family=quasibinomial(link = "logit"))

summary(m20.cluster1)

or.svyglm(m20.cluster1)

round(svytable(~water_supply_1, design = svy.cluster1), digits=0)

round(svytable(~water_supply_1+igg_zikv_total, design = svy.cluster1), digits=0)

round(prop.table(svytable(~water_supply_1+igg_zikv_total, design = svy.cluster1), margin = 1), digits = 3)

m21.cluster1<-svyglm(igg_zikv_total~water_supply_1, design=svy.cluster1,family=quasibinomial(link = "logit"))

summary(m21.cluster1)

m21.cluster1<-svyglm(igg_zikv_total~factor(water_supply_1), design=svy.cluster1,family=quasibinomial(link = "logit"))

summary(m21.cluster1)

or.svyglm(m21.cluster1)

round(svytable(~water_shortage, design = svy.cluster1), digits=0)

round(svytable(~water_shortage+igg_zikv_total, design = svy.cluster1), digits=0)

round(prop.table(svytable(~water_shortage+igg_zikv_total, design = svy.cluster1), margin = 1), digits = 3)

m22.cluster1<-svyglm(igg_zikv_total~water_shortage, design=svy.cluster1,family=quasibinomial(link = "logit"))

summary(m22.cluster1)

m22.cluster1<-svyglm(igg_zikv_total~factor(water_shortage), design=svy.cluster1,family=quasibinomial(link = "logit"))

summary(m22.cluster1)

or.svyglm(m22.cluster1)

round(svytable(~garbage_1, design = svy.cluster1), digits=0)

round(svytable(~garbage_1+igg_zikv_total, design = svy.cluster1), digits=0)

round(prop.table(svytable(~garbage_1+igg_zikv_total, design = svy.cluster1), margin = 1), digits = 3)

m23.cluster1<-svyglm(igg_zikv_total~garbage_1, design=svy.cluster1,family=quasibinomial(link = "logit"))

summary(m23.cluster1)

m23.cluster1<-svyglm(igg_zikv_total~factor(garbage_1), design=svy.cluster1,family=quasibinomial(link = "logit"))

summary(m23.cluster1)

or.svyglm(m23.cluster1)

round(svytable(~income_chef, design = svy.cluster1), digits=0)

round(svytable(~income_chef+igg_zikv_total, design = svy.cluster1), digits=0)

round(prop.table(svytable(~income_chef+igg_zikv_total, design = svy.cluster1), margin = 1), digits = 3)

m24.cluster1<-svyglm(igg_zikv_total~income_chef, design=svy.cluster1,family=quasibinomial(link = "logit"))

summary(m24.cluster1)

m24.cluster1<-svyglm(igg_zikv_total~factor(income_chef), design=svy.cluster1,family=quasibinomial(link = "logit"))

summary(m24.cluster1)

or.svyglm(m24.cluster1)

round(svytable(~education_chef, design = svy.cluster1), digits=0)

round(svytable(~education_chef+igg_zikv_total, design = svy.cluster1), digits=0)

round(prop.table(svytable(~education_chef+igg_zikv_total, design = svy.cluster1), margin = 1), digits = 3)

m25.cluster1<-svyglm(igg_zikv_total~education_chef, design=svy.cluster1,family=quasibinomial(link = "logit"))

summary(m25.cluster1)

m25.cluster1<-svyglm(igg_zikv_total~factor(education_chef), design=svy.cluster1,family=quasibinomial(link = "logit"))

summary(m25.cluster1)

or.svyglm(m25.cluster1)

round(svytable(~race_chef_2, design = svy.cluster1), digits=0)

round(svytable(~race_chef_2+igg_zikv_total, design = svy.cluster1), digits=0)

round(prop.table(svytable(~race_chef_2+igg_zikv_total, design = svy.cluster1), margin = 1), digits = 3)

m26.cluster1<-svyglm(igg_zikv_total~race_chef_2, design=svy.cluster1,family=quasibinomial(link = "logit"))

summary(m26.cluster1)

m26.cluster1<-svyglm(igg_zikv_total~factor(race_chef_2), design=svy.cluster1,family=quasibinomial(link = "logit"))

summary(m26.cluster1)

or.svyglm(m26.cluster1)

# CLUSTER 2

round(svytable(~numor_house_1, design = svy.cluster2), digits=0)

round(svytable(~numor_house_1+igg_zikv_total, design = svy.cluster2), digits=0)

round(prop.table(svytable(~numor_house_1+igg_zikv_total, design = svy.cluster2), margin = 1), digits = 3)

m18.cluster2<-svyglm(igg_zikv_total~numor_house_1, design=svy.cluster2,family=quasibinomial(link = "logit"))

summary(m18.cluster2)

m18.cluster2<-svyglm(igg_zikv_total~factor(numor_house_1), design=svy.cluster2,family=quasibinomial(link = "logit"))

summary(m18.cluster2)

or.svyglm(m18.cluster2)

round(svytable(~type_house_1, design = svy.cluster2), digits=0)

round(svytable(~type_house_1+igg_zikv_total, design = svy.cluster2), digits=0)

round(prop.table(svytable(~type_house_1+igg_zikv_total, design = svy.cluster2), margin = 1), digits = 3)

m19.cluster2<-svyglm(igg_zikv_total~type_house_1, design=svy.cluster2,family=quasibinomial(link = "logit"))

summary(m19.cluster2)

m19.cluster2<-svyglm(igg_zikv_total~factor(type_house_1), design=svy.cluster2,family=quasibinomial(link = "logit"))

summary(m19.cluster2)

or.svyglm(m19.cluster2)

round(svytable(~bathroom_waste_1, design = svy.cluster2), digits=0)

round(svytable(~bathroom_waste_1+igg_zikv_total, design = svy.cluster2), digits=0)

round(prop.table(svytable(~bathroom_waste_1+igg_zikv_total, design = svy.cluster2), margin = 1), digits = 3)

m20.cluster2<-svyglm(igg_zikv_total~bathroom_waste_1, design=svy.cluster2,family=quasibinomial(link = "logit"))

summary(m20.cluster2)

m20.cluster2<-svyglm(igg_zikv_total~factor(bathroom_waste_1), design=svy.cluster2,family=quasibinomial(link = "logit"))

summary(m20.cluster2)

or.svyglm(m20.cluster2)

round(svytable(~water_supply_1, design = svy.cluster2), digits=0)

round(svytable(~water_supply_1+igg_zikv_total, design = svy.cluster2), digits=0)

round(prop.table(svytable(~water_supply_1+igg_zikv_total, design = svy.cluster2), margin = 1), digits = 3)

m21.cluster2<-svyglm(igg_zikv_total~water_supply_1, design=svy.cluster2,family=quasibinomial(link = "logit"))

summary(m21.cluster2)

m21.cluster2<-svyglm(igg_zikv_total~factor(water_supply_1), design=svy.cluster2,family=quasibinomial(link = "logit"))

summary(m21.cluster2)

or.svyglm(m21.cluster2)

round(svytable(~water_shortage, design = svy.cluster2), digits=0)

round(svytable(~water_shortage+igg_zikv_total, design = svy.cluster2), digits=0)

round(prop.table(svytable(~water_shortage+igg_zikv_total, design = svy.cluster2), margin = 1), digits = 3)

m22.cluster2<-svyglm(igg_zikv_total~water_shortage, design=svy.cluster2,family=quasibinomial(link = "logit"))

summary(m22.cluster2)

m22.cluster2<-svyglm(igg_zikv_total~factor(water_shortage), design=svy.cluster2,family=quasibinomial(link = "logit"))

summary(m22.cluster2)

or.svyglm(m22.cluster2)

round(svytable(~garbage_1, design = svy.cluster2), digits=0)

round(svytable(~garbage_1+igg_zikv_total, design = svy.cluster2), digits=0)

round(prop.table(svytable(~garbage_1+igg_zikv_total, design = svy.cluster2), margin = 1), digits = 3)

m23.cluster2<-svyglm(igg_zikv_total~garbage_1, design=svy.cluster2,family=quasibinomial(link = "logit"))

summary(m23.cluster2)

m23.cluster2<-svyglm(igg_zikv_total~factor(garbage_1), design=svy.cluster2,family=quasibinomial(link = "logit"))

summary(m23.cluster2)

or.svyglm(m23.cluster2)

round(svytable(~income_chef, design = svy.cluster2), digits=0)

round(svytable(~income_chef+igg_zikv_total, design = svy.cluster2), digits=0)

round(prop.table(svytable(~income_chef+igg_zikv_total, design = svy.cluster2), margin = 1), digits = 3)

m24.cluster2<-svyglm(igg_zikv_total~income_chef, design=svy.cluster2,family=quasibinomial(link = "logit"))

summary(m24.cluster2)

m24.cluster2<-svyglm(igg_zikv_total~factor(income_chef), design=svy.cluster2,family=quasibinomial(link = "logit"))

summary(m24.cluster2)

or.svyglm(m24.cluster2)

round(svytable(~education_chef, design = svy.cluster2), digits=0)

round(svytable(~education_chef+igg_zikv_total, design = svy.cluster2), digits=0)

round(prop.table(svytable(~education_chef+igg_zikv_total, design = svy.cluster2), margin = 1), digits = 3)

m25.cluster2<-svyglm(igg_zikv_total~education_chef, design=svy.cluster2,family=quasibinomial(link = "logit"))

summary(m25.cluster2)

m25.cluster2<-svyglm(igg_zikv_total~factor(education_chef), design=svy.cluster2,family=quasibinomial(link = "logit"))

summary(m25.cluster2)

or.svyglm(m25.cluster2)

round(svytable(~race_chef_2, design = svy.cluster2), digits=0)

round(svytable(~race_chef_2+igg_zikv_total, design = svy.cluster2), digits=0)

round(prop.table(svytable(~race_chef_2+igg_zikv_total, design = svy.cluster2), margin = 1), digits = 3)

m26.cluster2<-svyglm(igg_zikv_total~race_chef_2, design=svy.cluster2,family=quasibinomial(link = "logit"))

summary(m26.cluster2)

m26.cluster2<-svyglm(igg_zikv_total~factor(race_chef_2), design=svy.cluster2,family=quasibinomial(link = "logit"))

summary(m26.cluster2)

or.svyglm(m26.cluster2)

# CLUSTER 3

round(svytable(~numor_house_1, design = svy.cluster3), digits=0)

round(svytable(~numor_house_1+igg_zikv_total, design = svy.cluster3), digits=0)

round(prop.table(svytable(~numor_house_1+igg_zikv_total, design = svy.cluster3), margin = 1), digits = 3)

m18.cluster3<-svyglm(igg_zikv_total~numor_house_1, design=svy.cluster3,family=quasibinomial(link = "logit"))

summary(m18.cluster3)

m18.cluster3<-svyglm(igg_zikv_total~factor(numor_house_1), design=svy.cluster3,family=quasibinomial(link = "logit"))

summary(m18.cluster3)

or.svyglm(m18.cluster3)

round(svytable(~type_house_1, design = svy.cluster3), digits=0)

round(svytable(~type_house_1+igg_zikv_total, design = svy.cluster3), digits=0)

round(prop.table(svytable(~type_house_1+igg_zikv_total, design = svy.cluster3), margin = 1), digits = 3)

m19.cluster3<-svyglm(igg_zikv_total~type_house_1, design=svy.cluster3,family=quasibinomial(link = "logit"))

summary(m19.cluster3)

m19.cluster3<-svyglm(igg_zikv_total~factor(type_house_1), design=svy.cluster3,family=quasibinomial(link = "logit"))

summary(m19.cluster3)

or.svyglm(m19.cluster3)

round(svytable(~bathroom_waste_1, design = svy.cluster3), digits=0)

round(svytable(~bathroom_waste_1+igg_zikv_total, design = svy.cluster3), digits=0)

round(prop.table(svytable(~bathroom_waste_1+igg_zikv_total, design = svy.cluster3), margin = 1), digits = 3)

m20.cluster3<-svyglm(igg_zikv_total~bathroom_waste_1, design=svy.cluster3,family=quasibinomial(link = "logit"))

summary(m20.cluster3)

m20.cluster3<-svyglm(igg_zikv_total~factor(bathroom_waste_1), design=svy.cluster3,family=quasibinomial(link = "logit"))

summary(m20.cluster3)

or.svyglm(m20.cluster3)

round(svytable(~water_supply_1, design = svy.cluster3), digits=0)

round(svytable(~water_supply_1+igg_zikv_total, design = svy.cluster3), digits=0)

round(prop.table(svytable(~water_supply_1+igg_zikv_total, design = svy.cluster3), margin = 1), digits = 3)

m21.cluster3<-svyglm(igg_zikv_total~factor(water_supply_1), design=svy.cluster3,family=quasibinomial(link = "logit"))

summary(m21.cluster3)

m21.cluster3<-svyglm(igg_zikv_total~factor(water_supply_1), design=svy.cluster3,family=quasibinomial(link = "logit"))

summary(m21.cluster3)

or.svyglm(m21.cluster3)

round(svytable(~water_shortage, design = svy.cluster3), digits=0)

round(svytable(~water_shortage+igg_zikv_total, design = svy.cluster3), digits=0)

round(prop.table(svytable(~water_shortage+igg_zikv_total, design = svy.cluster3), margin = 1), digits = 3)

m22.cluster3<-svyglm(igg_zikv_total~water_shortage, design=svy.cluster3,family=quasibinomial(link = "logit"))

summary(m22.cluster3)

m22.cluster3<-svyglm(igg_zikv_total~factor(water_shortage), design=svy.cluster3,family=quasibinomial(link = "logit"))

summary(m22.cluster3)

or.svyglm(m22.cluster3)

round(svytable(~garbage_1, design = svy.cluster3), digits=0)

round(svytable(~garbage_1+igg_zikv_total, design = svy.cluster3), digits=0)

round(prop.table(svytable(~garbage_1+igg_zikv_total, design = svy.cluster3), margin = 1), digits = 3)

m23.cluster3<-svyglm(igg_zikv_total~garbage_1, design=svy.cluster3,family=quasibinomial(link = "logit"))

summary(m23.cluster3)

m23.cluster3<-svyglm(igg_zikv_total~factor(garbage_1), design=svy.cluster3,family=quasibinomial(link = "logit"))

summary(m23.cluster3)

or.svyglm(m23.cluster3)

round(svytable(~income_chef, design = svy.cluster3), digits=0)

round(svytable(~income_chef+igg_zikv_total, design = svy.cluster3), digits=0)

round(prop.table(svytable(~income_chef+igg_zikv_total, design = svy.cluster3), margin = 1), digits = 3)

m24.cluster3<-svyglm(igg_zikv_total~income_chef, design=svy.cluster3,family=quasibinomial(link = "logit"))

summary(m24.cluster3)

m24.cluster3<-svyglm(igg_zikv_total~factor(income_chef), design=svy.cluster3,family=quasibinomial(link = "logit"))

summary(m24.cluster3)

or.svyglm(m24.cluster3)

round(svytable(~education_chef, design = svy.cluster3), digits=0)

round(svytable(~education_chef+igg_zikv_total, design = svy.cluster3), digits=0)

round(prop.table(svytable(~education_chef+igg_zikv_total, design = svy.cluster3), margin = 1), digits = 3)

m25.cluster3<-svyglm(igg_zikv_total~education_chef, design=svy.cluster3,family=quasibinomial(link = "logit"))

summary(m25.cluster3)

m25.cluster3<-svyglm(igg_zikv_total~factor(education_chef), design=svy.cluster3,family=quasibinomial(link = "logit"))

summary(m25.cluster3)

or.svyglm(m25.cluster3)

round(svytable(~race_chef_2, design = svy.cluster3), digits=0)

round(svytable(~race_chef_2+igg_zikv_total, design = svy.cluster3), digits=0)

round(prop.table(svytable(~race_chef_2+igg_zikv_total, design = svy.cluster3), margin = 1), digits = 3)

m26.cluster3<-svyglm(igg_zikv_total~race_chef_2, design=svy.cluster3,family=quasibinomial(link = "logit"))

summary(m26.cluster3)

m26.cluster3<-svyglm(igg_zikv_total~factor(race_chef_2), design=svy.cluster3,family=quasibinomial(link = "logit"))

summary(m26.cluster3)

or.svyglm(m26.cluster3)

##############

# TABLE 10 #

##############

# CLUSTER 1

round(svytable(~sex, design = svy.cluster1), digits=0)

round(svytable(~sex+igg_zikv_total, design = svy.cluster1), digits=0)

round(prop.table(svytable(~sex+igg_zikv_total, design = svy.cluster1), margin = 1), digits = 3)

m27.cluster1<-svyglm(igg_zikv_total~sex, design=svy.cluster1,family=quasibinomial(link = "logit"))

summary(m27.cluster1)

m27.cluster1<-svyglm(igg_zikv_total~factor(sex), design=svy.cluster1,family=quasibinomial(link = "logit"))

summary(m27.cluster1)

or.svyglm(m27.cluster1)

round(svytable(~idgrupo4, design = svy.cluster1), digits=0)

round(svytable(~idgrupo4+igg_zikv_total, design = svy.cluster1), digits=0)

round(prop.table(svytable(~idgrupo4+igg_zikv_total, design = svy.cluster1), margin = 1), digits = 3)

m28.cluster1<-svyglm(igg_zikv_total~idgrupo4, design=svy.cluster1,family=quasibinomial(link = "logit"))

summary(m28.cluster1)

m28.cluster1<-svyglm(igg_zikv_total~factor(idgrupo4), design=svy.cluster1,family=quasibinomial(link = "logit"))

summary(m28.cluster1)

or.svyglm(m28.cluster1)

round(svytable(~race_ind_2, design = svy.cluster1), digits=0)

round(svytable(~race_ind_2+igg_zikv_total, design = svy.cluster1), digits=0)

round(prop.table(svytable(~race_ind_2+igg_zikv_total, design = svy.cluster1), margin = 1), digits = 3)

m29.cluster1<-svyglm(igg_zikv_total~race_ind_2, design=svy.cluster1,family=quasibinomial(link = "logit"))

summary(m29.cluster1)

m29.cluster1<-svyglm(igg_zikv_total~factor(race_ind_2), design=svy.cluster1,family=quasibinomial(link = "logit"))

summary(m29.cluster1)

or.svyglm(m29.cluster1)

round(svytable(~education_ind, design = svy.cluster1), digits=0)

round(svytable(~education_ind+igg_zikv_total, design = svy.cluster1), digits=0)

round(prop.table(svytable(~education_ind+igg_zikv_total, design = svy.cluster1), margin = 1), digits = 3)

m30.cluster1<-svyglm(igg_zikv_total~education_ind, design=svy.cluster1,family=quasibinomial(link = "logit"))

summary(m30.cluster1)

m30.cluster1<-svyglm(igg_zikv_total~factor(education_ind), design=svy.cluster1,family=quasibinomial(link = "logit"))

summary(m30.cluster1)

or.svyglm(m30.cluster1)

#round(svytable(~income_ind_2, design = svy.cluster1), digits=0)

#round(svytable(~income_ind_2+igg_zikv_total, design = svy.cluster1), digits=0)

#round(prop.table(svytable(~income_ind_2+igg_zikv_total, design = svy.cluster1), margin = 1), digits = 3)

#m31.cluster1<-svyglm(igg_zikv_total~income_ind_2, design=svy.cluster1,family=quasibinomial(link = "logit"))

#summary(m31.cluster1)

#m31.cluster1<-svyglm(igg_zikv_total~factor(income_ind_2), design=svy.cluster1,family=quasibinomial(link = "logit"))

#summary(m31.cluster1)

#or.svyglm(m31.cluster1)

round(svytable(~mobility, design = svy.cluster1), digits=0)

round(svytable(~mobility+igg_zikv_total, design = svy.cluster1), digits=0)

round(prop.table(svytable(~mobility+igg_zikv_total, design = svy.cluster1), margin = 1), digits = 3)

m32.cluster1<-svyglm(igg_zikv_total~mobility, design=svy.cluster1,family=quasibinomial(link = "logit"))

summary(m32.cluster1)

m32.cluster1<-svyglm(igg_zikv_total~factor(mobility), design=svy.cluster1,family=quasibinomial(link = "logit"))

summary(m32.cluster1)

or.svyglm(m32.cluster1)

round(svytable(~repellent, design = svy.cluster1), digits=0)

round(svytable(~repellent+igg_zikv_total, design = svy.cluster1), digits=0)

round(prop.table(svytable(~repellent+igg_zikv_total, design = svy.cluster1), margin = 1), digits = 3)

m33.cluster1<-svyglm(igg_zikv_total~repellent, design=svy.cluster1,family=quasibinomial(link = "logit"))

summary(m33.cluster1)

m33.cluster1<-svyglm(igg_zikv_total~factor(repellent), design=svy.cluster1,family=quasibinomial(link = "logit"))

summary(m33.cluster1)

or.svyglm(m33.cluster1)

round(svytable(~dengue_exp, design = svy.cluster1), digits=0)

round(svytable(~dengue_exp+igg_zikv_total, design = svy.cluster1), digits=0)

round(prop.table(svytable(~dengue_exp+igg_zikv_total, design = svy.cluster1), margin = 1), digits = 3)

m34.cluster1<-svyglm(igg_zikv_total~dengue_exp, design=svy.cluster1,family=quasibinomial(link = "logit"))

summary(m34.cluster1)

m34.cluster1<-svyglm(igg_zikv_total~factor(dengue_exp), design=svy.cluster1,family=quasibinomial(link = "logit"))

summary(m34.cluster1)

or.svyglm(m34.cluster1)

# CLUSTER 2

round(svytable(~sex, design = svy.cluster2), digits=0)

round(svytable(~sex+igg_zikv_total, design = svy.cluster2), digits=0)

round(prop.table(svytable(~sex+igg_zikv_total, design = svy.cluster2), margin = 1), digits = 3)

m27.cluster2<-svyglm(igg_zikv_total~sex, design=svy.cluster2,family=quasibinomial(link = "logit"))

summary(m27.cluster2)

m27.cluster2<-svyglm(igg_zikv_total~factor(sex), design=svy.cluster2,family=quasibinomial(link = "logit"))

summary(m27.cluster2)

or.svyglm(m27.cluster2)

round(svytable(~idgrupo4, design = svy.cluster2), digits=0)

round(svytable(~idgrupo4+igg_zikv_total, design = svy.cluster2), digits=0)

round(prop.table(svytable(~idgrupo4+igg_zikv_total, design = svy.cluster2), margin = 1), digits = 3)

m28.cluster2<-svyglm(igg_zikv_total~idgrupo4, design=svy.cluster2,family=quasibinomial(link = "logit"))

summary(m28.cluster2)

m28.cluster2<-svyglm(igg_zikv_total~factor(idgrupo4), design=svy.cluster2,family=quasibinomial(link = "logit"))

summary(m28.cluster2)

or.svyglm(m28.cluster2)

round(svytable(~race_ind_2, design = svy.cluster2), digits=0)

round(svytable(~race_ind_2+igg_zikv_total, design = svy.cluster2), digits=0)

round(prop.table(svytable(~race_ind_2+igg_zikv_total, design = svy.cluster2), margin = 1), digits = 3)

m29.cluster2<-svyglm(igg_zikv_total~race_ind_2, design=svy.cluster2,family=quasibinomial(link = "logit"))

summary(m29.cluster2)

m29.cluster2<-svyglm(igg_zikv_total~factor(race_ind_2), design=svy.cluster2,family=quasibinomial(link = "logit"))

summary(m29.cluster2)

or.svyglm(m29.cluster2)

round(svytable(~education_ind, design = svy.cluster2), digits=0)

round(svytable(~education_ind+igg_zikv_total, design = svy.cluster2), digits=0)

round(prop.table(svytable(~education_ind+igg_zikv_total, design = svy.cluster2), margin = 1), digits = 3)

m30.cluster2<-svyglm(igg_zikv_total~education_ind, design=svy.cluster2,family=quasibinomial(link = "logit"))

summary(m30.cluster2)

m30.cluster2<-svyglm(igg_zikv_total~factor(education_ind), design=svy.cluster2,family=quasibinomial(link = "logit"))

summary(m30.cluster2)

or.svyglm(m30.cluster2)

#round(svytable(~income_ind_2, design = svy.cluster2), digits=0)

#round(svytable(~income_ind_2+igg_zikv_total, design = svy.cluster2), digits=0)

#round(prop.table(svytable(~income_ind_2+igg_zikv_total, design = svy.cluster2), margin = 1), digits = 3)

#m31.cluster2<-svyglm(igg_zikv_total~income_ind_2, design=svy.cluster2,family=quasibinomial(link = "logit"))

#summary(m31.cluster2)

#m31.cluster2<-svyglm(igg_zikv_total~factor(income_ind_2), design=svy.cluster2,family=quasibinomial(link = "logit"))

#summary(m31.cluster2)

#or.svyglm(m31.cluster2)

round(svytable(~mobility, design = svy.cluster2), digits=0)

round(svytable(~mobility+igg_zikv_total, design = svy.cluster2), digits=0)

round(prop.table(svytable(~mobility+igg_zikv_total, design = svy.cluster2), margin = 1), digits = 3)

m32.cluster2<-svyglm(igg_zikv_total~mobility, design=svy.cluster2,family=quasibinomial(link = "logit"))

summary(m32.cluster2)

m32.cluster2<-svyglm(igg_zikv_total~factor(mobility), design=svy.cluster2,family=quasibinomial(link = "logit"))

summary(m32.cluster2)

or.svyglm(m32.cluster2)

round(svytable(~repellent, design = svy.cluster2), digits=0)

round(svytable(~repellent+igg_zikv_total, design = svy.cluster2), digits=0)

round(prop.table(svytable(~repellent+igg_zikv_total, design = svy.cluster2), margin = 1), digits = 3)

m33.cluster2<-svyglm(igg_zikv_total~repellent, design=svy.cluster2,family=quasibinomial(link = "logit"))

summary(m33.cluster2)

m33.cluster2<-svyglm(igg_zikv_total~factor(repellent), design=svy.cluster2,family=quasibinomial(link = "logit"))

summary(m33.cluster2)

or.svyglm(m33.cluster2)

round(svytable(~dengue_exp, design = svy.cluster2), digits=0)

round(svytable(~dengue_exp+igg_zikv_total, design = svy.cluster2), digits=0)

round(prop.table(svytable(~dengue_exp+igg_zikv_total, design = svy.cluster2), margin = 1), digits = 3)

m34.cluster2<-svyglm(igg_zikv_total~dengue_exp, design=svy.cluster2,family=quasibinomial(link = "logit"))

summary(m34.cluster2)

m34.cluster2<-svyglm(igg_zikv_total~factor(dengue_exp), design=svy.cluster2,family=quasibinomial(link = "logit"))

summary(m34.cluster2)

or.svyglm(m34.cluster2)

# CLUSTER 3

round(svytable(~sex, design = svy.cluster3), digits=0)

round(svytable(~sex+igg_zikv_total, design = svy.cluster3), digits=0)

round(prop.table(svytable(~sex+igg_zikv_total, design = svy.cluster3), margin = 1), digits = 3)

m27.cluster3<-svyglm(igg_zikv_total~sex, design=svy.cluster3,family=quasibinomial(link = "logit"))

summary(m27.cluster3)

m27.cluster3<-svyglm(igg_zikv_total~factor(sex), design=svy.cluster3,family=quasibinomial(link = "logit"))

summary(m27.cluster3)

or.svyglm(m27.cluster3)

round(svytable(~idgrupo4, design = svy.cluster3), digits=0)

round(svytable(~idgrupo4+igg_zikv_total, design = svy.cluster3), digits=0)

round(prop.table(svytable(~idgrupo4+igg_zikv_total, design = svy.cluster3), margin = 1), digits = 3)

m28.cluster3<-svyglm(igg_zikv_total~idgrupo4, design=svy.cluster3,family=quasibinomial(link = "logit"))

summary(m28.cluster3)

m28.cluster3<-svyglm(igg_zikv_total~factor(idgrupo4), design=svy.cluster3,family=quasibinomial(link = "logit"))

summary(m28.cluster3)

or.svyglm(m28.cluster3)

round(svytable(~race_ind_2, design = svy.cluster3), digits=0)

round(svytable(~race_ind_2+igg_zikv_total, design = svy.cluster3), digits=0)

round(prop.table(svytable(~race_ind_2+igg_zikv_total, design = svy.cluster3), margin = 1), digits = 3)

m29.cluster3<-svyglm(igg_zikv_total~race_ind_2, design=svy.cluster3,family=quasibinomial(link = "logit"))

summary(m29.cluster3)

m29.cluster3<-svyglm(igg_zikv_total~factor(race_ind_2), design=svy.cluster3,family=quasibinomial(link = "logit"))

summary(m29.cluster3)

or.svyglm(m29.cluster3)

round(svytable(~education_ind, design = svy.cluster3), digits=0)

round(svytable(~education_ind+igg_zikv_total, design = svy.cluster3), digits=0)

round(prop.table(svytable(~education_ind+igg_zikv_total, design = svy.cluster3), margin = 1), digits = 3)

m30.cluster3<-svyglm(igg_zikv_total~education_ind, design=svy.cluster3,family=quasibinomial(link = "logit"))

summary(m30.cluster3)

m30.cluster3<-svyglm(igg_zikv_total~factor(education_ind), design=svy.cluster3,family=quasibinomial(link = "logit"))

summary(m30.cluster3)

or.svyglm(m30.cluster3)

#round(svytable(~income_ind_2, design = svy.cluster3), digits=0)

#round(svytable(~income_ind_2+igg_zikv_total, design = svy.cluster3), digits=0)

#round(prop.table(svytable(~income_ind_2+igg_zikv_total, design = svy.cluster3), margin = 1), digits = 3)

#m31.cluster3<-svyglm(igg_zikv_total~income_ind_2, design=svy.cluster3,family=quasibinomial(link = "logit"))

#summary(m31.cluster3)

#m31.cluster3<-svyglm(igg_zikv_total~factor(income_ind_2), design=svy.cluster3,family=quasibinomial(link = "logit"))

#summary(m31.cluster3)

#or.svyglm(m31.cluster3)

round(svytable(~mobility, design = svy.cluster3), digits=0)

round(svytable(~mobility+igg_zikv_total, design = svy.cluster3), digits=0)

round(prop.table(svytable(~mobility+igg_zikv_total, design = svy.cluster3), margin = 1), digits = 3)

m32.cluster3<-svyglm(igg_zikv_total~mobility, design=svy.cluster3,family=quasibinomial(link = "logit"))

summary(m32.cluster3)

m32.cluster3<-svyglm(igg_zikv_total~factor(mobility), design=svy.cluster3,family=quasibinomial(link = "logit"))

summary(m32.cluster3)

or.svyglm(m32.cluster3)

round(svytable(~repellent, design = svy.cluster3), digits=0)

round(svytable(~repellent+igg_zikv_total, design = svy.cluster3), digits=0)

round(prop.table(svytable(~repellent+igg_zikv_total, design = svy.cluster3), margin = 1), digits = 3)

m33.cluster3<-svyglm(igg_zikv_total~repellent, design=svy.cluster3,family=quasibinomial(link = "logit"))

summary(m33.cluster3)

m33.cluster3<-svyglm(igg_zikv_total~factor(repellent), design=svy.cluster3,family=quasibinomial(link = "logit"))

summary(m33.cluster3)

or.svyglm(m33.cluster3)

round(svytable(~dengue_exp, design = svy.cluster3), digits=0)

round(svytable(~dengue_exp+igg_zikv_total, design = svy.cluster3), digits=0)

round(prop.table(svytable(~dengue_exp+igg_zikv_total, design = svy.cluster3), margin = 1), digits = 3)

m34.cluster3<-svyglm(igg_zikv_total~dengue_exp, design=svy.cluster3,family=quasibinomial(link = "logit"))

summary(m34.cluster3)

m34.cluster3<-svyglm(igg_zikv_total~factor(dengue_exp), design=svy.cluster3,family=quasibinomial(link = "logit"))

summary(m34.cluster3)

or.svyglm(m34.cluster3)

##############

# TABLE 11 #

##############

round(svytable(~cluster, design = svy), digits=0)

# ANALISE MULTIVARIADA (INCLUINDO APENAS AS VARIAVEIS COM P<=0,25 NA ANALISE BIVARIADA POR BLOCOS) #

# CLUSTER 1

mf2.b1.cluster1<-stepAIC(svyglm(igg_zikv_total~factor(numor_house_1)+factor(type_house_1)+factor(water_supply_1)+factor(water_shortage)+factor(garbage_1)+factor(income_chef)+factor(education_chef)+factor(race_chef_2), design=svy.cluster1,family=quasibinomial(link = "logit")), direction = c("backward"))

summary(mf2.b1.cluster1)

mf2.b1.cluster1<-stepAIC(svyglm(igg_zikv_total~factor(type_house_1)+factor(garbage_1)+factor(education_chef), design=svy.cluster1,family=quasibinomial(link = "logit")), direction = c("backward"))

summary(mf2.b1.cluster1)

mf2.b1.cluster1<-stepAIC(svyglm(igg_zikv_total~factor(type_house_1)+factor(garbage_1)+factor(education_chef), design=svy.cluster1,family=quasibinomial(link = "logit")), direction = c("backward"))

summary(mf2.b1.cluster1)

mf2.b2.cluster1<-stepAIC(svyglm(igg_zikv_total~factor(idgrupo4)+factor(race_ind_2)+factor(education_ind)+factor(mobility)+factor(dengue_exp), design=svy.cluster1,family=quasibinomial(link = "logit")), direction = c("backward"))

summary(mf2.b2.cluster1)

mf2.b2.cluster1<-stepAIC(svyglm(igg_zikv_total~factor(dengue_exp), design=svy.cluster1,family=quasibinomial(link = "logit")), direction = c("backward"))

summary(mf2.b2.cluster1)

mf2.b.cluster1<-svyglm(igg_zikv_total~factor(type_house_1)+factor(garbage_1)+factor(education_chef)+factor(dengue_exp), design=svy.cluster1,family=quasibinomial(link = "logit"))

or.svyglm(mf2.b.cluster1)

# CLUSTER 2

mf2.b1.cluster2<-stepAIC(svyglm(igg_zikv_total~factor(type_house_1)+factor(water_supply_1)+factor(water_shortage)+factor(income_chef)+factor(education_chef), design=svy.cluster2,family=quasibinomial(link = "logit")),direction = c("backward"))

summary(mf2.b1.cluster2)

mf2.b2.cluster2<-stepAIC(svyglm(igg_zikv_total~factor(idgrupo4)+factor(race_ind_2)+factor(education_ind)+factor(mobility)+factor(dengue_exp), design=svy.cluster2,family=quasibinomial(link = "logit")),direction = c("backward"))

summary(mf2.b2.cluster2)

mf2.b.cluster2<-svyglm(igg_zikv_total~factor(type_house_1)+factor(income_chef)+factor(idgrupo4)+factor(education_ind)+factor(dengue_exp), design=svy.cluster2,family=quasibinomial(link = "logit"))

or.svyglm(mf2.b.cluster2)

# CLUSTER 3

mf2.b1.cluster3<-stepAIC(svyglm(igg_zikv_total~factor(type_house_1)+factor(income_chef)+factor(race_chef_2), design=svy.cluster3,family=quasibinomial(link = "logit")),direction = c("backward"))

summary(mf2.b1.cluster3)

mf2.b2.cluster3<-stepAIC(svyglm(igg_zikv_total~factor(idgrupo4)+factor(race_ind_2)+factor(education_ind)+factor(mobility)+factor(repellent)+factor(dengue_exp), design=svy.cluster3,family=quasibinomial(link = "logit")),direction = c("backward"))

summary(mf2.b2.cluster3)

mf2.b.cluster3<-svyglm(igg_zikv_total~factor(income_chef)+factor(idgrupo4)+factor(dengue_exp), design=svy.cluster3,family=quasibinomial(link = "logit"))

or.svyglm(mf2.b.cluster3)
