# Supplementary material for: Seroprevalence of Dengue, Chikungunya and Zika at the epicenter of the congenital microcephaly epidemic in Northeast Brazil: A population-based survey
Source: PLoS Negl Trop Dis. 2023 Jul 3;17(7):e0011270. doi: 10.1371/journal.pntd.0011270 (PMC10348596; doi:10.1371/journal.pntd.0011270)
Supplement: Attachment — Submitted filename: Braga_et_all_referre_response-EM-05-17-23.docx [file pntd.0011270.s011.docx]

**Editor comments**

As you will see from their reports, the reviewers’ recommendations are mixed. While referees #2 and #3 enthusiastically support publication, referee #1 considers that your study is flawed and rejected its publication in PNTD. After carefully reading your study, I agree with reviewers #2 and #3 who recommend that a revised version of the manuscript can be reconsidered. If you think that you can deal satisfactorily with the criticisms on revision, I would be pleased to see a revised manuscript. For further clarification, I ask that you respond to the following comment of reviewer #1: *"In a dengue endemic population where all four dengue viruses have been transmitted, a survey of antibodies will detect many, many different patterns of prior infections.  Distinguishing these patterns and attributing them to primary or secondary (dengue then Zika or Zika then dengue) will be very difficult and will require much more foundational data than is presented here"*

**Response:**

Dear Editors Dr. Tonelli and Dr. Viennet

Thank you for taking so much attention on the comments of all three reviewers and give us an opportunity to respond. The order of infection is irrelevant for our study and we do not attempt to determine the order of dengue and Zika infections, neither makes any conclusion related to order of infection, and therefore the statement *"In a dengue endemic population where all four dengue viruses have been transmitted, a survey of antibodies will detect many, many different patterns of prior infections.  Distinguishing these patterns and attributing them to primary or secondary (dengue then Zika or Zika then dengue) will be very difficult and will require much more foundational data than is presented here"* is not germane to our article. Nevertheless, we have reported the proportion of dengue serotype specific seroprevalence in Recife, Brazil in pregnant women, children, and general population (1-3) and the proportion of each serotype changes with age due to the sequential introduction of each serotype, DENV1 in the 1980’s, DENV2 in the 1990’s, DENV3 in 2000’s and DENV4 in 2010’s. The ZIKV naïve samples used to measure the ZIKV assay specificity included serum from people from all ages and SES, in acute and convalescent infection. All this information is clear in the article. All the assays that we used were validated and calibrated using well characterized samples from our multiple cohorts that are ongoing for 20 years in this area. The 95% confidence interval of the sensitivity and specificity of the assays were included in the and the seroprevalence analyzes and adjusted accordingly.

We have incorporated all the suggestions of reviewers #2 and #3 and made the correction indicated by reviewer #1 “Line 229. “indetermined” – undetermined”. A revised clean version of the manuscript, incorporating the reviewer’s comments and some grammatical corrections was submitted as well as.

**Reviewer #1:** In a dengue endemic population where all four dengue viruses have been transmitted, a survey of antibodies will detect many, many different patterns of prior infections.  Distinguishing these patterns and attributing them to primary or secondary (dengue then Zika or Zika then dengue) will be very difficult and will require much more foundational data than is presented here. The serological methods used do not describe the attributes of primary or secondary dengue infections or show how they can be identified, nor does it describe how dengue antibodies can be distinguished from antibody responses to Zika virus infections in individuals who either were seronegative or immune to one or more dengue viruses when infected. This cannot be done, as alleged, by use of algorithms.  What is required are robust and reproducible serological methods.

**Response.**

1) *Distinguishing these patterns and attributing them to primary or secondary (dengue then Zika or Zika then dengue) will be very difficult.* *The serological methods used do not describe the attributes of primary or secondary dengue infections or show how they can be identified,*

We have not attempted to distinguish the dengue serotype patterns, neither attributed as primary or secondary infection in this article. The order of infection, dengue or Zika or the dengue serotype is irrelevant for this study. The goal of the study is to determine the proportion of the total population infected by dengue (any serotype), Zika and Chikungunya. We have not stratified the dataset according to all possible combinations of viral infection and this is not the objective of this article.

2) *How dengue antibodies can be distinguished from antibody responses to Zika virus infections in individuals who either were seronegative or immune to one or more dengue viruses when infected. This cannot be done, as alleged, by use of algorithms.  What is required are robust and reproducible serological methods.*

The serological assays used for dengue and Zika have been described in previous studies, which showed that these assays can distinguish dengue from Zika infections and vice-versa. The sensitivity and specificity of these assays are published in the articles cited in our manuscript (4-7). We have not used any algorithm for testing. All samples were tested with all assays and the methods used are robust and reproducible. Moreover, as abundance of caution, the cut-offs of each assay were optimized using a standard panel of 240 samples representative of the local population and include samples from acute and convalescent infections. The dengue assay we developed was also used by Sanofi Pasteur to analyze the level of protection of their vaccine in dengue naïve and dengue primed population and this assay was accepted by the FDA, the cross-reactivity with Zika was very carefully analyzed and demonstrated in the article our aticles. Moreover the 95% confidence intervals of the calculated sensitivity and specificity for each assay were used to further adjust the calculated seroprevalence to account cross-reactivity issues.

**Reviewer #2:** This article is the result of a very interesting and breakthrough study examining the seroprevalence of Dengue, Chikungunya and Zika in the Northeast of Brazil, a very poor region, at the epicenter of the congenital microcephaly epidemic. The study required the design of an appropriate methodological strategy to describe and discuss the consequences of simultaneous exposure and vulnerability to the three arboviral infections in the same population, in different age groups and socio-economic strata. The methodological strategy is clear and precise, supported by a well-designed population-based survey. The objectives are clearly presented and articulated with the testable hypotheses stated. The population is described in a clear and precise way, appropriate for the hypotheses being tested. The statistical analysis used to support conclusions is correct and well-conceived. There are no concerns about ethical or regulatory requirements.

**Response:**

We thank the reviewer #2 for its sensible and accurate review.

**Reviewer #3:** The manuscript Seroprevalence of Dengue, Chikungunya and Zika at the epicenter of the congenital microcephaly epidemic in Northeast Brazil: a population-based survey shows relevant aspects of the transmission dynamic from a hyperendemic arbovirus from Brazil, where the main urban arbovirus, dengue, Zika, and Chikungunya, has coincided. As part of the methodology, the authors chose a seroprevalence study to identify the principal risk factors associated with arbovirus infection by adopting a multistage random stratification in two steps. First selecting, the units of study (named census tract CT), and in the second stage, the households that included all residents' target age of the study. With this methodology, the authors have gotten a sample size that allowed to address with enough power the hypothesis of the study. Also, the authors observed all ethical aspects of the research protocol.

**Response**

We thank the reviewer #3 for its sensible and accurate review.

**Reviewer #1:** This is a flawed attempt to describe recent dengue 1 – 4, chikungunya and Zika virus epidemiology in residents of Recife, Brazil, by measuring prevalence of antibodies generated by endemic DENVs and virgin-soil outbreaks of chikungunya and Zika viruses. How to distinguish dengue from Zika antibodies and vice versa in humans is a well-known unsolved serological problem.  This is the crucial challenge of this manuscript. It is a challenge the authors have failed. Dengue viruses circulating in Recife for several decades have produced the age-specific serological data displayed in Figure 4 A. The similar seroprevalence of age specific antibodies shown in Figures 4A and 4B are remarkable. Both describe age specific antibody distribution characteristic of virus endemicity.   The increased prevalence of “Zika antibodies” in older aged individuals shown in Figure 4 B can only be explained by Zika antigenic cross-reactions with antibodies in sera likely resulting from two or more dengue infections. The Zika age-specific antibody prevalence curve should look like the antibody prevalence curve in Figure 3 C, antibodies following a single introduction into the population of chikungunya virus.  Despite possible low level Zika virus infection in 2018 – 19, there is no evidence of vigorous Zika endemicity after 2016. The Zika epidemiology described in Brazil, therefore, is a classical single source epidemic.

**Response:**

1) *This is a flawed attempt to describe recent dengue 1 – 4, chikungunya and Zika virus epidemiology in residents of Recife, Brazil, by measuring prevalence of antibodies generated by endemic DENVs and virgin-soil outbreaks of chikungunya and Zika viruses.* *How to distinguish dengue from Zika antibodies and vice versa in humans is a well-known unsolved serological problem.*

We respectfully disagree with the reviewer for the reasons mentioned in a previous response. The reviewer #1 comment is very vague and does not explain what is the flaw of the assay is flawed. The level of cross-reactivity between dengue and Zika of the assays were carefully detemined and taken in consideration in the analyzes. There are several properly validated and published assays that can distinguish between dengue (4-19) and Zika infections, some of those with EUA authorization by European regulatory agencies, by ANVISA, the Brazilian regulatory agency, and others. Including ones that are authorized for use in triage of previous dengue infection to receive the Sanofi Pasteur Dengvaxia immunization that is available in Brazil, although not included in the national immunization program. We agree that the level of cross-reactivity during acute infections of Zika or dengue makes the diagnostic more difficult, but this is not the case of this study, all samples are from asymptomatic participants at the time of collection. The anti-Zika NS1 IgG3 assay are markers for recent infections (past 4 to 6 months) have been demonstrated for Dengue, Zika and HIV (5, 20-24). However, these marker are not indication of acute infection.

2) *Dengue viruses circulating in Recife for several decades have produced the age-specific serological data displayed in Figure 4 A. The similar seroprevalence of age specific antibodies shown in Figures 4A and 4B are remarkable. Both describe age specific antibody distribution characteristic of virus endemicity.   The increased prevalence of “Zika antibodies” in older aged individuals shown in Figure 4 B can only be explained by Zika antigenic cross-reactions with antibodies in sera likely resulting from two or more dengue infections. The Zika age-specific antibody prevalence curve should look like the antibody prevalence curve in Figure 3 C, antibodies following a single introduction into the population of chikungunya virus.  Despite possible low level Zika virus infection in 2018 – 19, there is no evidence of vigorous Zika endemicity after 2016. The Zika epidemiology described in Brazil, therefore, is a classical single source epidemic.*

**Response**

We respectfully disagree with the reviewer #1. DENV1 was introduced in Pernambuco in 1980’s, DENV2 in the 1990’s. DENV3 in 2000, and DENV4 in the 2010. All four dengue serotypes have only been present together in the state of Pernambuco and Recife for a little more than one decade and have not reached a steady-state transmission of all four serotypes. It is expected to take at least more ten to fifteen years (25). The Dengue seroprevalence by age is very similar in low and intermediary social strata and while the high social strata has much lower seroprevalence and never really achieves the same levels of the low and medium SES. The Zika seroprevalence by age is lower than the dengue in all strata. At younger age, <10 years old, the Zika seroprevalence starts at approximately 25% while dengue starts at 50%. By age 25, the dengue seroprevalence reaches approximately 80%, while Zika seroprevalence in low and medium SES reaches 50%, a level that dengue reached at 5 years of age, and the maximum seroprevalence that the high SES reaches in approximately 40% at age of 65. In addition, Zika seroprevalence of low and medium social strata grows by age but much slower than the dengue seroprevalence. The difference between the attack rates of Zika and Dengue in the high social strata is very large. In summary, this data does not support the reviewer hypothesis. Zika virus as well as Chikungunya continue to circulate in Recife and Pernambuco. According to the state of Pernambuco arboviral surveillance there were 4.570 Zika, 34.369 of Chikungunya and 56.902 dengue case cases notified in 2022. It is also important to note that the proportion of asymptomatic Zika infection is much greater than dengue and hence much more sub-notified. ZIKV and CHIKV continue to be notified every year since 2016. The reviewer maybe confusing Zika infection with microcephaly cases.

**Specific comments:**

*Line 115 -120. The authors cite erroneous dates of virus introduction. Data provided by PAHO document chikungunya virus introduction into the Americas and widespread infection beginning in 2014. CHIK infections continued at a high rate through 2016 and at a low rate until the present time.* *Zika virus was introduced in 2015, spreading with high incidence in 2016 but sharply declining in 2017. The authors describe this sequence exactly in reverse. The confusion between dates of entry of Zika and chikungunya viruses into Brazil confounds this study and requires repair.*

**Response**

We respectfully disagree with the reviewer. The introduction of Chikungunya in the Americas was not in Brazil, and the first cases of Chikungunya in Brazil, was a year later and was not in Recife. The epidemic of Chikungunya arrived in Recife after the Zika epidemic as reported previously (26).

Line 127.  “Susceptibility*”*

**Response**

the issue was not found.

Line 229. “indetermined” – undetermined.

**Response**

Corrected

**Reviewer #2:** The results of this population-based survey are relevant and an important contribution to critical and still open questions in the literature, unveiling the dramatic consequences of simultaneous exposure of the study population to the three arboviral diseases. These results raise intriguing issues highlighted by the authors, related to the different profiles of the epidemics and the differences in exposure and vulnerability among age groups, sex and socio-economic strata. These results confirmed continued DENV transmission and intense ZIKV and CHIKV transmission during the 2015/2016 epidemics followed by ongoing low-level transmission. Issues related to herd immunity and antibody detection thresholds were examined and the results indicate a concerning scenario: a significant proportion of the population is likely still susceptible to be infected by ZIKV and CHIKV.  Other intriguing issues stressed by the authors refer to the reasons underlying cease of the ZIKV epidemic in 2017/18, still an open question. The analysis is well presented and clearly matches the analysis plan. The results are clearly and completely presented. The Figures and Tables are clear and well designed.

**Response**

We agree with reviewer #2 that it still unclear the reason for the end of Zika epidemic. But we postulate that some facts that might have played a role: 1) the arrival of CHIVK epidemic (26) and 2) partial protection mediated by anti-dengue antibodies might have played a role (4). Although it was not the objective of this study to determine the factor mediating.

**Reviewer #3:** Yes. The results were coherent with the analysis plan, and the reader can easily understand the contents in the tables and figures.

**Response**

We thank the reviewer #3 for its sensible and accurate review.

**Reviewer #1:** This attempt to describe the serological status of the population of Recife, Brazil is not accurate as it is based upon flawed methods.

**Response**

We respectfully disagree with reviewer #1 for the reasons mentioned above.

**Reviewer #2:** In their conclusion, the authors underline the high vulnerability of the study population to these arboviral infections in the Northeast of Brazil and provide evidence of the persistence of the co-circulation of ZIKV and CHIKV in this highly urbanized setting, three years after the peak of ZIKV epidemic, alerting to the risk of future outbreaks. Considering the large proportion of susceptible population for ZIKV and CHIKV, they stress the urgent need for Aedes surveillance and control and development and efficacy trials of vaccines against these arboviruses.  The conclusions are well elaborated and supported by the data presented. The authors discuss and clearly explain how the data presented and the results of the study can be helpful to advance our understanding of the topic. Limitations of the analysis are clearly described, indicating the need for further research in this area.

**Response**

We thank the reviewer #3 for its sensible and accurate review.

**Reviewer #3:** The data presented confirmed a high level of exposition of the population against the main urban flavivirus infection. It further shows the level of heterogeneity of the transmission in the city's different economic and social strata. Bisanzio D et al, 2018 (Spatio-temporal coherence of dengue, chikungunya and Zika outbreaks in Merida, Mexico. **PLoS Neglected Tropical Diseases 12(3): e0006298.** [**https://doi.org/**](https://doi.org/) **10.1371/journal.pntd.0006298)** also demonstrated this heterogeneity in the arbovirus dynamics transmission. Applying different methodologies, the authors also identified areas with additional risk for dengue infection named hotspots. In a longitudinal cohort study the status for DENV infection in 505 children was compared between those residing inside and outside the hotspot areas. Living inside the hotspot area was associated with a significantly higher infection probability than living outside (odds ratio, 1.71 [95%CI, 1.08–2.20]; p < 0.05). Similarly, in Recife ZIKV infection, the estimated force of infection was 2.4 times 449 greater in the low socioeconomic strata when compared to the high strata. This aspect is highly relevant because it can guide local strategies in terms of policies for the prevention and control of these diseases. Moreover, focusing efforts in high transmission areas has the additional benefit of increased efficiency by better allocating limited personnel and financial resources.

**Response**

We agree the reviewer #3 and thank for the information, we will discuss results in the context of the reference mentioned above and cite. The data from Bizansio et all study is undoubtedly relevant as they show differential risk of arboviral infection in urban spaces and point to the need to reinforce control measures in areas with a higher risk of transmission. Unlike Bizansio's study, which analyzed geocoded data of symptomatic cases notified by the passive surveillance system of the Mexican Ministry of Health’s, our study (a cross-sectional survey) population was selected through a cluster sampling process in multiple stages. This sample design is ideal for Spatio-temporal analysis, however we are investigating the possibilities to combine the data from our two seroprevalence surveys and notification data to identify hotspots.

**Reviewer #3:** Regarding chikungunya, the authors might explore further aspects of the inapparent infection. **Bustos Carrillo F et al. 2019 (Epidemiological evidence for lineage-specific differences in the risk of inapparent chikungunya virus infection. J Virol 93:e01622-18.** [**https://doi.org/10.1128/JVI**](https://doi.org/10.1128/JVI) **.01622-18)** provide epidemiological evidence that chikungunya epidemics are strongly influenced by CHIKV lineage, particularly with respect to the proportion of inapparent CHIKV infections at the population level. Suggest that the authors identify in the article the predominant CHICKV strain during the epidemics in Recife and compare the results of inapparent infections with the literature.

**Response**

We thank the reviewer to bringing this point to attention and the suggestion. We will include the CHIKV strain type and discuss in the context of the reference suggested. We have sequenced CHIKV viral RNA from serum samples of 132 patients. We have not performed detailed genomic analyzes but all strains are from the East/Central/South Africa (ECSA) group, that has shown 3 to 28% of inapparent infections and recent work in Nicaragua showed a 19% of inapparent infections associated with this strain (27). The preliminary results were presented in Seattle at the ASTMH meeting Abstract Book. GENETIC DETERMINANTS OF A CHIKUNGUYNA VIRUS OUTBREAK IN NORTHEAST BRAZIL, Chilinh Nguyen, Gregory D. Ebel, Ernesto TA Marques, Cynthia Braga, Thomas Jaenisch, Brian D. Foy, Tereza Magalhaes. Poster 0136, p43.

Response:
--------------------

Use this section for editorial suggestions as well as relatively minor modifications of existing data that would enhance clarity. If the only modifications needed are minor and/or editorial, you may wish to recommend “Minor Revision” or “Accept”.

Reviewer #1: needs more science.

Reviewer needs more attention.

Reviewer #2: (No Response)

Reviewer #3: No suggestions

--------------------

<b>Summary and General Comments</b></br></br>

Use this section to provide overall comments, discuss strengths/weaknesses of the study, novelty, significance, general execution, and scholarship. You may also include additional comments for the author, including concerns about dual publication, research ethics, or publication ethics. If requesting major revision, please articulate the new experiments that are needed.

Reviewer #1: This is a flawed study.

Reviewer makes this affirmation without any basis.

Reviewer #2: (No Response)

Reviewer #3: No comment

**References**

1.	Castanha PMS, Montarroyos UR, Silveira SMM, Albuquerque GDM, Mello MJG, Lopes KGS, et al. Incidence and risk factors for Dengue virus (DENV) infection in the first 2 years of life in a Brazilian prospective birth cohort. Epidemiol Infect. 2017;145(14):2971-9.

2.	Castanha PM, Braga C, Cordeiro MT, Souza AI, Silva CD, Jr., Martelli CM, et al. Placental Transfer of Dengue Virus (DENV)-Specific Antibodies and Kinetics of DENV Infection-Enhancing Activity in Brazilian Infants. J Infect Dis. 2016;214(2):265-72.

3.	Castanha PM, Cordeiro MT, Martelli CM, Souza WV, Marques ET, Jr., Braga C. Force of infection of dengue serotypes in a population-based study in the northeast of Brazil. Epidemiol Infect. 2013;141(5):1080-8.

4.	Rodriguez-Barraquer I, Costa F, Nascimento EJM, Nery N, Castanha PMS, Sacramento GA, et al. Impact of preexisting dengue immunity on Zika virus emergence in a dengue endemic region. Science. 2019;363(6427):607-10.

5.	Nascimento EJM, Huleatt JW, Cordeiro MT, Castanha PMS, George JK, Grebe E, et al. Development of antibody biomarkers of long term and recent dengue virus infections. J Virol Methods. 2018;257:62-8.

6.	Nascimento EJM, George JK, Velasco M, Bonaparte MI, Zheng L, DiazGranados CA, et al. Development of an anti-dengue NS1 IgG ELISA to evaluate exposure to dengue virus. J Virol Methods. 2018;257:48-57.

7.	Morales I, Rosenberger KD, Magalhaes T, Morais CNL, Braga C, Marques ETA, et al. Diagnostic performance of anti-Zika virus IgM, IgAM and IgG ELISAs during co-circulation of Zika, dengue, and chikungunya viruses in Brazil and Venezuela. PLoS Negl Trop Dis. 2021;15(4):e0009336.

8.	Alexander LW, Ben-Shachar R, Katzelnick LC, Kuan G, Balmaseda A, Harris E, et al. Boosting can explain patterns of fluctuations of ratios of inapparent to symptomatic dengue virus infections. Proc Natl Acad Sci U S A. 2021;118(14).

9.	Echegaray F, Laing P, Hernandez S, Marquez S, Harris A, Laing I, et al. Adapting Rapid Diagnostic Tests to Detect Historical Dengue Virus Infections. Front Immunol. 2021;12:703887.

10.	Katzelnick LC, Zambrana JV, Elizondo D, Collado D, Garcia N, Arguello S, et al. Dengue and Zika virus infections in children elicit cross-reactive protective and enhancing antibodies that persist long term. Sci Transl Med. 2021;13(614):eabg9478.

11.	Katzelnick LC, Bos S, Harris E. Protective and enhancing interactions among dengue viruses 1-4 and Zika virus. Curr Opin Virol. 2020;43:59-70.

12.	Katzelnick LC, Narvaez C, Arguello S, Lopez Mercado B, Collado D, Ampie O, et al. Zika virus infection enhances future risk of severe dengue disease. Science. 2020;369(6507):1123-8.

13.	Andrade P, Gimblet-Ochieng C, Modirian F, Collins M, Cárdenas M, Katzelnick LC, et al. Impact of pre-existing dengue immunity on human antibody and memory B cell responses to Zika. Nat Commun. 2019;10(1):938.

14.	Bustos Carrillo FA, Mercado BL, Monterrey JC, Collado D, Saborio S, Miranda T, et al. Epidemics of chikungunya, Zika, and COVID-19 reveal bias in case-based mapping. medRxiv. 2022.

15.	Liao T, Wang X, Donolato M, Harris E, Cruz MM, Balmaseda A, et al. Evaluation of ViroTrack Sero Zika IgG/IgM, a New Rapid and Quantitative Zika Serological Diagnostic Assay. Diagnostics (Basel). 2020;10(6).

16.	Balmaseda A, Zambrana JV, Collado D, García N, Saborío S, Elizondo D, et al. Comparison of Four Serological Methods and Two Reverse Transcription-PCR Assays for Diagnosis and Surveillance of Zika Virus Infection. J Clin Microbiol. 2018;56(3).

17.	Goncalves A, Peeling RW, Chu MC, Gubler DJ, de Silva AM, Harris E, et al. Innovative and New Approaches to Laboratory Diagnosis of Zika and Dengue: A Meeting Report. J Infect Dis. 2018;217(7):1060-8.

18.	Premkumar L, Collins M, Graham S, Liou GA, Lopez CA, Jadi R, et al. Development of Envelope Protein Antigens To Serologically Differentiate Zika Virus Infection from Dengue Virus Infection. J Clin Microbiol. 2018;56(3).

19.	Chotiwan N, Brewster CD, Magalhaes T, Weger-Lucarelli J, Duggal NK, Rückert C, et al. Rapid and specific detection of Asian- and African-lineage Zika viruses. Sci Transl Med. 2017;9(388).

20.	Ximenes RAA, Miranda-Filho DB, Brickley EB, Montarroyos UR, Martelli CMT, Araújo TVB, et al. Zika virus infection in pregnancy: Establishing a case definition for clinical research on pregnant women with rash in an active transmission setting. PLoS Negl Trop Dis. 2019;13(10):e0007763.

21.	Castanha PMS, Souza WV, Braga C, Araujo TVB, Ximenes RAA, Albuquerque M, et al. Perinatal analyses of Zika- and dengue virus-specific neutralizing antibodies: A microcephaly case-control study in an area of high dengue endemicity in Brazil. PLoS Negl Trop Dis. 2019;13(3):e0007246.

22.	Rodriguez-Barraquer I, Costa F, Nascimento EJM, Nery NJ, Castanha PMS, Sacramento GA, et al. Impact of preexisting dengue immunity on Zika virus emergence in a dengue endemic region. Science. 2019;363(6427):607-10.

23.	Viana IFT, Coêlho DF, Palma ML, Nascimento EJM, Gu G, Lima LFO, et al. Detection of IgG3 antibodies specific to the human immunodeficiency virus type 1 (HIV-1) p24 protein as marker for recently acquired infection. Epidemiol Infect. 2018;146(10):1293-300.

24.	Viana IFT, Coelho DF, Palma ML, Nascimento EJM, Gu G, Lima LFO, et al. Detection of IgG3 antibodies specific to the human immunodeficiency virus type 1 (HIV-1) p24 protein as marker for recently acquired infection. Epidemiol Infect. 2018;146(10):1293-300.

25.	Rodriguez-Barraquer I, Cordeiro MT, Braga C, de Souza WV, Marques ET, Cummings DA. From re-emergence to hyperendemicity: the natural history of the dengue epidemic in Brazil. PLoS Negl Trop Dis. 2011;5(1):e935.

26.	Magalhaes T, Braga C, Cordeiro MT, Oliveira ALS, Castanha PMS, Maciel APR, et al. Zika virus displacement by a chikungunya outbreak in Recife, Brazil. PLoS Negl Trop Dis. 2017;11(11):e0006055.

27.	Bustos Carrillo F, Collado D, Sanchez N, Ojeda S, Lopez Mercado B, Burger-Calderon R, et al. Epidemiological Evidence for Lineage-Specific Differences in the Risk of Inapparent Chikungunya Virus Infection. J Virol. 2019;93(4).
